# Global, Regional, and National Estimates of Nutritional Deficiency Burden among Reproductive Women from 2010 to 2019

**DOI:** 10.3390/nu14040832

**Published:** 2022-02-16

**Authors:** Shengchao Jiang, Jingjing Liu, Xinye Qi, Rizhen Wang, Xing Wang, Kexin Wang, Qiao Xu, Peiwen Chen, Nan Meng, Qunhong Wu, Linghan Shan

**Affiliations:** 1 Department of Health Policy, Health Management College, Harbin Medical University, Harbin 150081, China; jshengchao1@163.com (S.J.); 15001290091@163.com (J.L.); qixinye1992@163.com (X.Q.); wrz_99766@163.com (R.W.); wangkexin1013@126.com (K.W.); xuqiaodeyouxiang@163.com (Q.X.); cpw279@126.com (P.C.); 18845144958@163.com (N.M.); 2 Department of Social Medicine, School of Public Health, Harbin Medical University Harbin, Harbin 150081, China; 3 The Fourth Affiliated Hospital, School of Medicine, Zhejiang University, Hangzhou 310014, China; wx1454164617@163.com

**Keywords:** reproductive women, nutritional deficiencies, disability-adjusted life years

## Abstract

Women of reproductive age (15–49 years) are often considered a vulnerable population affected by nutritional deficiencies, impairing their health and that of their offspring. We briefly introduced (a) the incidence and disability-adjusted life years (DALYs) trends from 2010 to 2019 and (b) the correlation between sex differences and income levels and nutritional deficiencies of reproductive women firstly. Notably, the burden of overall nutritional deficiencies among reproductive women remained generally stable from 2010 to 2019, whereas the iodine and vitamin A deficiencies as a subcategory were associated with increased incidence rates and DALYs, respectively. A significant increasing trend occurred in South Asia, Southeast Asia, and Turkey for incidence, and Western Sub-Saharan Africa and Zimbabwe had a strong increase for DALYs. Further analysis of the correlation between nutritional deficiency incidence and economic capacity showed that they were not correlated with the income of women themselves, as was the result of income difference with men. The results of this study will help to identify gaps in nutritional deficiency burden among reproductive women and facilitate the development of regional or national responses. Compared with economic capital, macroscopic political guarantees and social and cultural capital are important measures to remedy the nutritional deficiencies of reproductive women.

## 1. Introduction

Nutritional deficiencies are generally considered to include two major forms—protein–energy malnutrition and micronutrition deficiencies—although there is no universal agreement on the definition and clinical assessment of these forms [1], and these deficiencies still remain an important public health problem for women of reproductive age (15–49 years). Limited epidemiological evidence suggests that nearly one-third of reproductive women worldwide have experienced anemia and iodine deficiency in recent years [2,3]. In a study of nutritional deficiencies among reproductive women in Africa, it was found that the prevalence rates of folic acid, zinc, iodine and vitamin A deficiencies were 46%, 34%, 22–55%, and 4–22%, respectively [4]. Nutritional deficiencies in reproductive women bring challenges to achieving the goal of the United Nations Sustainable Development: ‘address the nutritional needs of teenage girls and pregnant and lactating women by 2030’.

Nutritional deficiencies during the reproductive age negatively affect women’s maternal metabolism and tissue proliferation, and fetal growth and development [5]. The World Health Organization (WHO) recommends a series of measures against nutritional deficiencies among reproductive women through health education, food fortification, and nutrient supplementation [2,6]. Correspondingly, different regions and countries have begun efforts to improve nutrition concerns and reduce nutritional deficiency risk among reproductive women. Determining the levels and trends in the burden of nutritional deficiencies among reproductive women is required to appropriately guide efforts to improve nutritional deficiencies at regional and national levels. However, there are limited published reports on nutritional deficiencies in women of reproductive age (single nutritional deficiency subcategories of the disease spectrum and incomplete reporting data) or a lack of analysis on temporal changes [3,6].

Therefore, we aimed to provide a comprehensive estimate of the burden for nutritional deficiencies (including main subcategories) of reproductive women at the global, income, regional, and national levels from 2010 to 2019 through a secondary analysis of the Global Burden of Diseases, Injuries, and Risk Factors Study 2019 (GBD 2019). The specific contents of the study included the following: (1) the estimated incidence and disability-adjusted life years (DALYs) among women of reproductive age, (2) age-standardized incidence and DALYs rates of nutritional deficiencies among women of reproductive age in 2019, (3) trend of age-standardized incidence and DALYs rates from 2010 to 2019, and (4) we also explored the correlation between nutritional deficiency incidence among women of reproductive age and gross national income per capita of women themselves and correlation with income difference with men.

## 2. Method

### 2.1. Overview

The GBD 2019 study used all available latest sources of epidemiological survey data and optimized standardized methods for comparative assessment of health loss and associated risk factors for 282 causes of death, 354 causes of years lived with disability (YLDs), and 359 causes of DALYs in 205 countries and territories from 2010 to 2019. Details of the GBD 2019 method have been published elsewhere [4]. The original data were estimated by the GBD for nutritional deficiencies from censuses, household surveys, civil registration and vital statistics, disease registries, health service use, air pollution monitors, satellite images, and disease notifications.

Nutritional deficiencies were identified based on the 10th revision of the International Classification of Diseases and Injuries (ICD-10), coded as D50–D53.9, E00–E02, E40–E46.9, E50–E61.9, E63–E64.9, and Z13.2–Z13.3. Its main subcategories included protein–energy malnutrition (coded as E40–E46.9, E64.0), iodine deficiencies (coded as E00–E02), vitamin A deficiencies (coded as E50–E50.9, E64.1), dietary iron deficiencies (coded as D50–D50.9), and other nutritional deficiencies (coded as D51–D53.9, E51–E61.9, E63–E64, E64.2–E64.9).

### 2.2. Measures

Two parameters associated with nutritional deficiencies were measured: incidence and DALYs. The incidence refers to the number of new cases of a given cause during a given period in a specified population. DALYs were derived by summing years of life lost (YLLs) and YLDs, thereby combining premature death and health-related suffering to describe the total number of years of healthy life lost due to various reasons.

The age-standardized rate (ASR) refers to the method of the population according to the same standard age composition, which aims to eliminate the influence of different population age compositions and ensure the comparability of statistical indicators. The age-standardized incidence rate represents the number of new cases per 100,000 persons, and the age-standardized DALYs rate represents the YLDs and YLLs per 100,000 persons after age standardization.

### 2.3. Gross National Income per Capita Set

Economic level is considered an important factor affecting women’s power, purchasing power, nutrition choices, and access to nutritional supplements [7,8,9]. In this study, we explored the correlation between the incidence of nutritional deficiencies among reproductive women and gross national income per capita in women and the correlation with the gross national income per capita gap between women and men. The gross national income per capita data for women and men were acquired from the United Nations Development Program (http://hdr.undp.org/en/data, accessed on 20 August 2021).

### 2.4. Statistical Analyses

We used a global standard (WHO 2000–2025) to calculate ASRs according to the following equation [10]:

$$\mathrm{ASR}=\frac{\sum_{i=1}^{A}a_{\mathrm{iwi}}}{\sum_{i=1}^{A}\mathrm{wi}},\;$$

where ${a\;}_{i}$ and $w_{i}$ represent the age-specific rates and the number of persons (or weight) in the same age subgroup of the selected reference standard population (where i denotes the ith age class), respectively.

The estimated annual percentage change (EAPC), which is a good indicator of the ASR trend [11], was calculated using the following formula:

y=a+bx+∈ and 

EAPC = 100 × (exp (β) − 1),

where y = ln(ASR), x is the calendar year, and β is the estimated value of the slope, b. The above EAPC formula was then applied to calculate the 95% confidence interval (CI), and the standard error was obtained from the fitted regression line. If the estimation of the EAPC and the lower boundary of its 95% CI were both >0, the ASR was considered to have an increasing trend. In contrast, if the estimation of the EAPC and upper boundary of its 95% CI were both <0, the ASR was considered to have a downward trend. Otherwise, the ASR was considered stable over time.

We analyzed all incidence and DALYs data using SAS 9.4 statistical software (SAS Institute Inc., Cary, NC, USA) and R version 3.3.

## 3. Results

### 3.1. The Burden of Nutritional Deficiencies among Reproductive Women at Global Level

**Figure 1:**
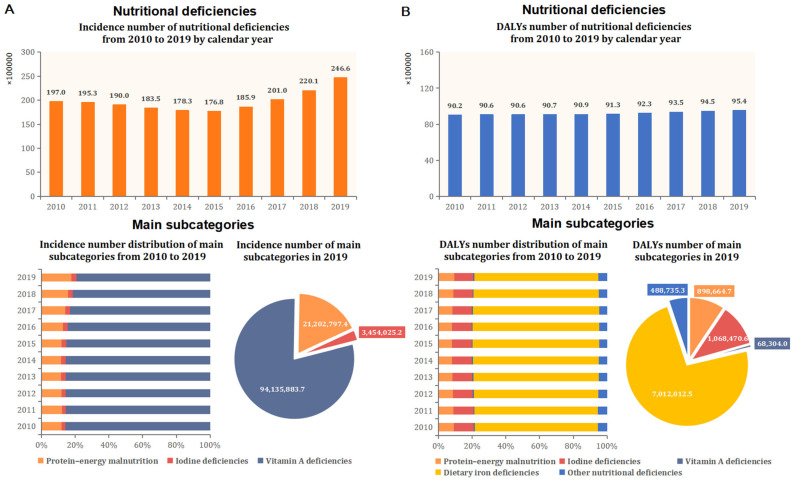
Global incidence and DALYs number attributed to nutritional deficiencies and main subcategories by calendar year from 2010 to 2019. (**A**) Incidence; (**B**) DALYs. DALYs = disability-adjusted life years.

**Figure 2:**
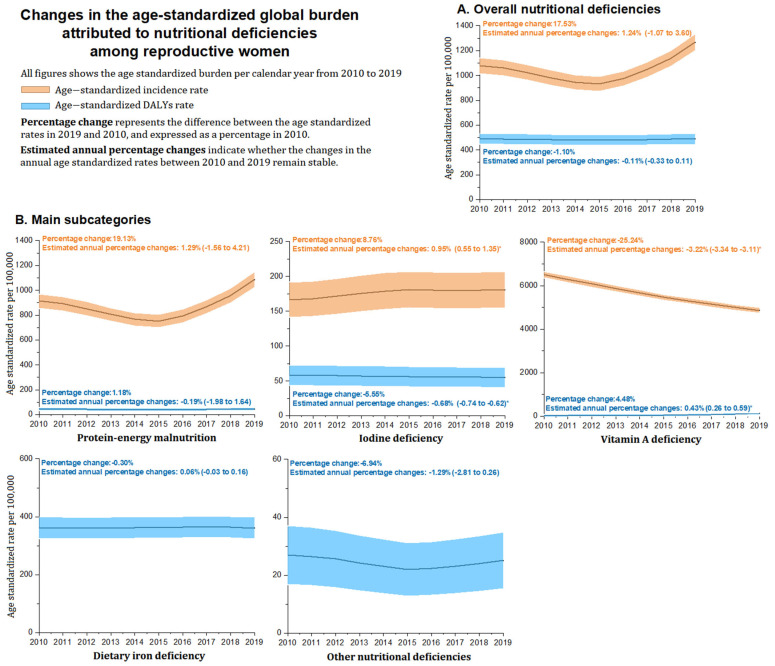
The global trend of age-standardized incidence rate and age-standardized DALYs rate (per 100,000) attributed to nutritional deficiencies and main subcategories from 2010 to 2019. (**A**) Overall nutritional deficiencies; (**B**) Main subcategories. DALYs = disability-adjusted life years. Note: (*) Indicates statistically significant trend (*p* < 0.05).

Table 1 and Figure 1 and Figure 2 provide the global estimated number, ASR of incidence, and DALYs for overall nutritional deficiencies among reproductive women in 2019 and their EAPC from 2010 to 2019. Globally, the number of incidence cases of overall nutritional deficiencies was 24.66 million among women of reproductive age in 2019, with a 25.18% increase in absolute number from 2010 to 2019 (Table 1 and Figure 1). The age-standardized incidence rate for nutritional deficiencies was 1268.51 per 100,000 population and remained stable from 2010 to 2019. In all subcategories of nutritional deficiencies, vitamin A deficiency had the highest age-standardized incidence rate (4864.81 per 100,000), followed by protein–energy malnutrition (1087.65 per 100,000) and iodine deficiency (180.87 per 100,000). From 2010 to 2019, the only increase in the age-standardized incidence rate was observed for iodine deficiency (EAPC, 0.95 [0.55 to 1.35]), whereas the only decreased subcategory was observed for vitamin A deficiency (EAPC, −3.22 [−3.34 to −3.11]) (Table 1 and Figure 2).

The number of DALYs cases of nutritional deficiencies was 9.54 million in 2019, with a 4.68% increase in the past 10 years (Table 1 and Figure 1). The age-standardized DALYs rate of nutritional deficiencies was 490.71 per 100,000 in 2019 and remained stable from 2010 to 2019. In subcategory distribution, dietary iron deficiency contributed to the highest age-standardized DALYs rate (361.15 per 100,000) in all subcategories, followed by iodine deficiency (54.84 per 100,000), protein–energy malnutrition (46.08 per 100,000), other nutritional deficiencies (25.10 per 100,000), and vitamin A deficiency (3.53 per 100,000). From 2010 to 2019, the age-standardized DALYs rate for iodine deficiency showed the only decreased subcategory (EAPC, −0.68 [−0.74 to −0.62]), whereas vitamin A deficiency increased slightly by 0.43% (EAPC, 0.43 [0.26 to 0.59]). In addition, other subcategories of age-standardized DALYs rate remained stable (Table 1 and Figure 2).

### 3.2. The Burden of Nutritional Deficiencies at Income Level Stratified by the World Bank

**Figure 3:**
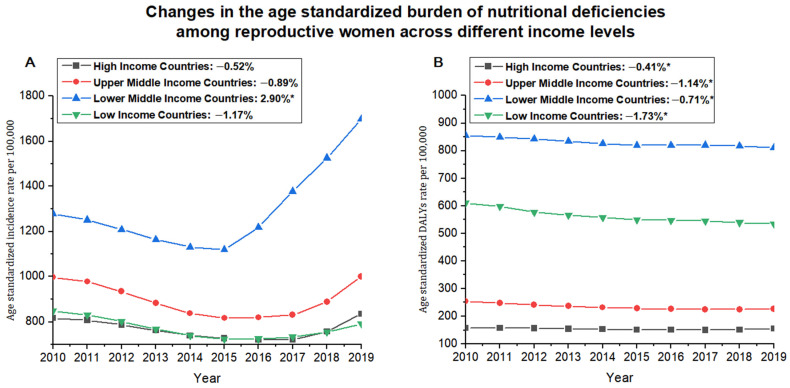
The trend of age-standardized incidence rate and age-standardized DALYs rate (per 100,000) attributed to nutritional deficiencies across different income level countries from 2010 to 2019. (**A**) Age-standardized incidence rate; (**B**) Age-standardized DALYs rate. DALYs = disability-adjusted life years. Note: (*) Indicates statistically significant trend (*p* < 0.05).

At the World Bank income level, the lower middle-income level had the highest age-standardized incidence rate for nutritional deficiencies (1698.33 per 100,000) in 2019, whereas the low-income level was observed the lowest estimate (791.08 per 100,000). Except for the increased trend that occurred in the lower middle-income level (EAPC, 2.90 [0.07 to 5.82]), the age-standardized incidence rate for nutritional deficiencies in the other three income groups generally kept stable from 2010 to 2019 (Table 1 and Figure 3). For the main subcategories of nutritional deficiencies, the age-standardized incidence rate of vitamin A deficiency kept the highest subcategories in four income groups with low-income level ranked first (15,119.48 per 100,000). From 2010 to 2019, all subcategories in the four income groups almost declined or remained stable, whereas only increased iodine deficiency occurred in the lower middle-income level (EAPC, 1.40 [0.67 to 2.13]) (Table 1).

The lower middle-income level had the highest age-standardized DALYs rate for overall nutritional deficiencies (811.38 per 100,000) in 2019, whereas the high-income level was observed at the lowest estimate (154.88 per 100,000). Over the past decade, four income levels all showed downward trends, especially in low-income levels, with the most significant decrease (EAPC, −1.38 [−1.73 to −1.02]) (Table 1 and Figure 3). Dietary iron deficiency contributed the heaviest DALYs among other subcategories in four income groups, and lower middle-income level showed the worst DALYs attributed to dietary iron deficiency (623.41 per 100,000) in 2019. From 2010 to 2019, all subcategories in the four income groups almost declined or remained stable, whereas the vitamin A deficiency was increased in the high-income (EAPC, 3.05 [2.67 to 3.44]) and upper middle-income (EAPC, 0.84 [0.60 to 1.09]) levels and dietary iron deficiency was increased in low-income (EAPC, 0.32 [0.24 to 0.41]) (Table 1).

### 3.3. The Burden of Nutritional Deficiencies at Regional Level

**Figure 4:**
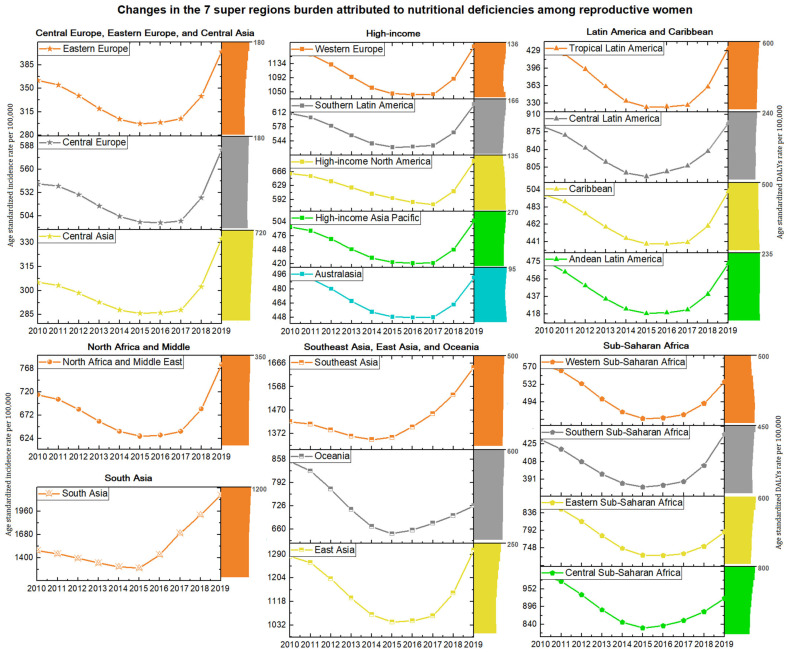
The regional incidence number, and trend of age-standardized incidence rate and age-standardized DALYs rate (per 100,000) attributed to nutritional deficiencies from 2010 to 2019. DALYs = disability-adjusted life years.

In 2019, South Asia (2149.96 per 100,000) ranked first in age-standardized incidence rate for overall nutritional deficiencies, whereas Central Asia was the lowest (332.28 per 100,000) (Table 1 and Figure 4). During the study period, the age-standardized incidence rates in South Asia (EAPC, 3.98 [0.49 to 7.59]) and Southeast Asia (EAPC, 1.37 [0.07 to 2.68]) increased; however, the decline was observed in only two regions: Eastern Sub-Saharan Africa (EAPC, −1.43 [−2.58 to −0.26]) and Oceania (EAPC, −2.13 [−3.96 to −0.27]) (Table 1 and Figure 4). Eighteen regions had the highest age-standardized incidence rate estimate for vitamin A deficiency compared to other subcategories, among which Eastern Sub-Saharan Africa ranked first (18,955.34 per 100,000) in 2019. Except for protein–energy malnutrition in Southeast Asia (EAPC, 1.61 [0.26 to 2.98]) and iodine deficiency in South Asia (EAPC, 1.7 [0.82 to 2.58]) with an increasing trend, the incidence rates of other subcategories remained stable or declined during the study period (Table 1).

The highest age-standardized DALYs rate for overall nutritional deficiencies was observed in South Asia (1102.44 per 100,000); in contrast, the lowest estimates were observed in Australasia (94.46 per 100,000) in 2019. The strongly increased trend occurred in Western Sub-Saharan Africa (EAPC, 1.71 [1.24 to 2.18]) and high-income North America (EAPC, 1.03 [0.80 to 1.26]) and Caribbean (EAPC, 0.44 [0.32 to 0.55]) from 2010 to 2019. Other regions remained stable or declined, and the fastest decline was observed in Andean Latin America (EAPC, −2.49 [−3.37 to −1.59]) (Table 1 and Figure 4). Except for Central Sub-Saharan Africa, which showed the highest age-standardized DALYs rate for iodine deficiency among all subcategories, the other 13 regions all had the highest DALYs rate for dietary iron deficiency, and South Asia (849.05 per 100,000) ranked first in 2019. Notably, in the past decade, the age-standardized DALYs rate of vitamin A deficiency in nine regions still increased, among which high-income North America (EAPC, 3.02 [2.34 to 3.70]) and high-income Asia Pacific (EAPC, 3.02 [2.34 to 3.70]) showed the most significant increase (Table 1).

### 3.4. The Burden of Nutritional Deficiencies at Country Level

**Figure 5:**
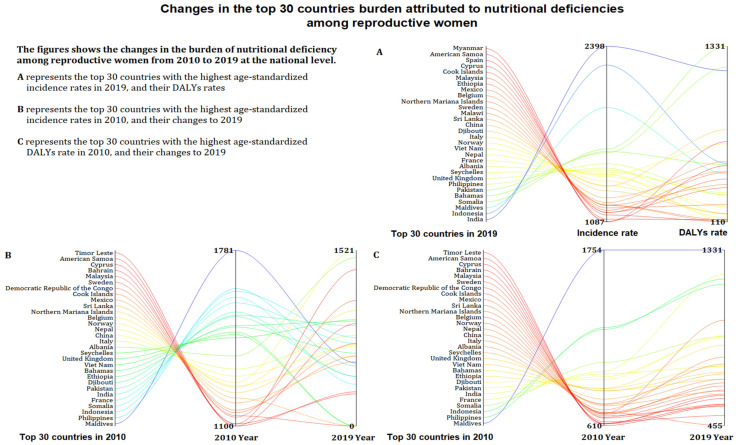
The age-standardized incidence rate and age-standardized DALYs rate (per 100,000) in 2019, and their change from 2010 to 2019 in the top 30 countries attributed to nutritional deficiencies among reproductive women. DALYs = disability-adjusted life years. (**A**) Top 30 countries with the highest age-standardized incidence rates and their age-standardized DALYs rate in 2019. (**B**) The top 30 countries with the highest age-standardized incidence rates in 2010, and their age-standardized incidence rates in 2019. (**C**) The top 30 countries with the highest age-standardized DALYs rates in 2010, and their age-standardized DALYs rates in 2019.

At the country level, India (2398.26 per 100,000) ranked first in the age-standardized incidence rate for overall nutritional deficiencies, whereas Belarus (278.44 per 100,000) was the lowest (Figure 5 and Supplementary Table S1). From 2010 to 2019, 92 countries showed an increased incidence rate, and the fastest estimate occurred in Turkey (EAPC, 5.1 [4.08 to 6.14]). Moreover, 172 countries had the highest age-standardized incidence rate for vitamin A deficiency, and Somalia (51,411.19 per 100,000) showed the heaviest estimate in 2019. The age-standardized incidence rate for protein–energy malnutrition in 102 countries and iodine deficiency in 17 countries increased. Except Zimbabwe (EAPC, 1.31 [0.98 to 1.65]) still on an upward trend, vitamin A deficiency incidence in other countries all decreased (Supplementary Table S1).

The highest age-standardized DALYs rate for overall nutritional deficiencies in 2019 was observed in Somalia (1330.95 per 100,000), whereas the lowest was observed in Greece (73.52 per 100,000) (Figure 5 and Supplementary Table S1). The age-standardized DALYs rate for overall nutritional deficiencies in 20 countries increased, in which Zimbabwe (EAPC, 4.44 [3.64 to 5.25]) showed the fastest increase. Dietary iron deficiency contributed to the highest age-standardized DALYs rate in 182 countries among other subcategories, and the highest DALYs burden was observed in Yemen (1051.85 per 100,000). The age-standardized DALYs rate for protein energy malnutrition in 59 countries, iodine deficiency in 18 countries, and dietary iron deficiency in 21 countries still increased (Supplementary Table S1).

### 3.5. Association between Nutritional Deficiency Incidence among Women of Reproductive Age with Gross National Income per Capita

**Figure 6:**
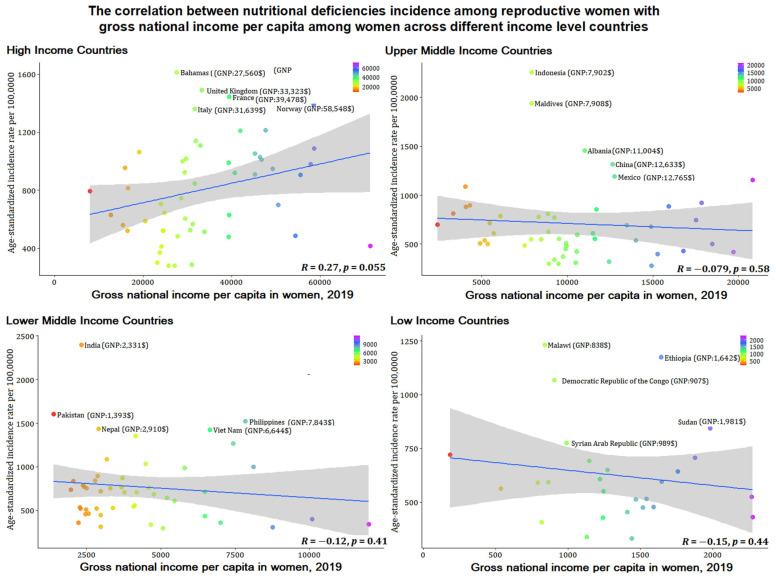
The correlation between age-standardized incidence rates per 100,000 for nutritional deficiencies among reproductive women with gross national income per capita in women in 2019 across different income level countries.

**Figure 7:**
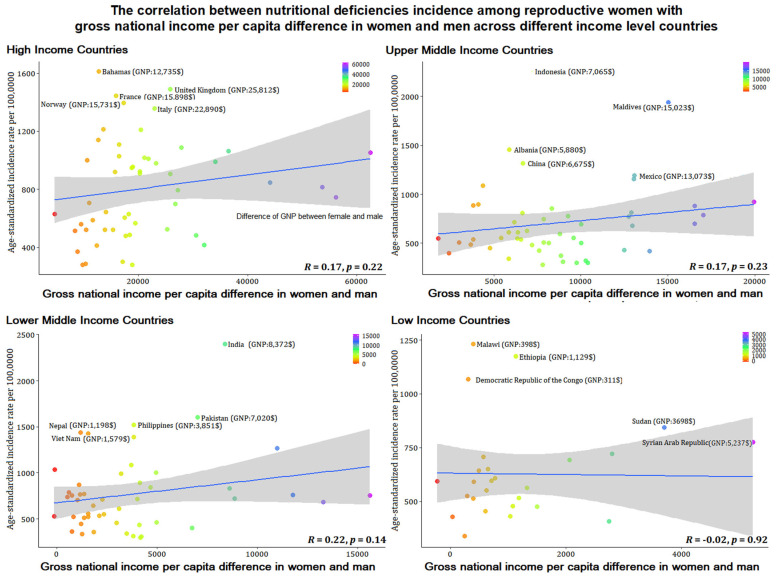
The correlation between age-standardized incidence rates per 100,000 for nutritional deficiencies among reproductive women with gross national income per capita difference in women and men across different income level countries.

Figure 6 and Figure 7 show the correlation between the age-standardized incidence rate of nutritional deficiencies among reproductive women and gross national income per capita of women themselves and the correlation with gross national income per capita difference across women and men. The results showed that the incidence of nutritional deficiencies among reproductive women was not correlated with income (*p* > 0.05).

## 4. Discussion

We comprehensively and systematically assessed the burden of nutritional deficiencies among reproductive women at the global, regional, and national levels. Globally, the disease burden of age-standardized incidence rate and age-standardized DALYs rate for overall nutritional deficiencies remained stable from 2010 to 2019. In the main subcategories of nutritional deficiencies, the age-standardized incidence rate for iodine deficiency increased significantly, and the age-standardized DALYs rate caused by vitamin A deficiency represented the only increased subcategory.

The time-stable trends of nutritional deficiencies burden that we observed for women of reproductive age need to be interpreted with caution, which suggests that the prevention of nutritional deficiencies in women who are not yet deficient in the reproductive period should be paid more attention. The first ‘World Declaration and Plan of Action on Nutrition’ had made significant progress in improving women’s nutrition and health. However, insufficient dietary diversity and limited food choices, early marriage and childbearing, maternal nutrition, postpartum diet, disease, and childcare still affect the nutritional status of women during the reproductive period. In addition, the targeted supplementary plan for women of reproductive age was slightly inadequate. The WHO report showed that of the more than 150 countries that implement vitamin and supplement programs, only 39% target women of reproductive age [2]. Compared with the more than 80% of countries adopting targeted programs for children’s nutritional deficiencies, less than half of the countries report supplementary programs for women of reproductive age. It is worth noting that childbirth care and family planning are the most unequally distributed services among the reproductive health services offered to women of different socioeconomic statuses, ethnicities, and ages. In the United Nations Sustainable Development Goals, there were only a few indicators (such as SDG 3.7.1) that focused on reproductive health and rights to achieve gender equality and empower all women and girls.

Notably, although the vitamin A deficiency incidence rate has declined significantly in the past decade, it still remained the heaviest subcategory of nutritional deficiency worldwide in 2019. Supplementation, fortification, and dietary diversification are considered to be the three main ways to improve vitamin A status. However, in the general practice of controlling vitamin A deficiency, there are still problems while achieving success: policies in many countries have reduced maternal vitamin A deficiency, but coverage rate tends to be low, there is inadequate governmental support and supervision of vitamin A supplementation of reproductive women [12,13], and postharvest handling and cooking practices of food cause the loss of vitamin A [14]. In addition, the traditional dietary patterns, cooking methods and external food environment, differences in the frequency of intake of vegetables and fruits among different ethnic groups, and cultural adaptation of immigrant individuals affect vitamin A intake in different countries and regions [15,16,17,18].

Notably, the global increased trend of incidence attributable to iodine deficiency among reproductive from 2010 to 2019. The increase may be caused by declining iodine intake as a result of reductions in salt consumption and increased consumption of processed foods and condiments made without iodized salt. The WHO, United Nations International Children’s Emergency Fund, and International Council for Control of Iodine Deficiency Disorders recommend that all edible salt for human and animal consumption, including food processing, be iodized [19]. However, a review of the use of iodized salt by the food processing industry [20] found that only approximately one-third to one-half of the salt used in food processing was iodized. In addition, the high temperature during cooking and processing can cause iodine loss in the salt.

An interesting phenomenon was found in the classification by different income level that economic ability was not consistent with the nutritional deficiencies epidemic and DALYs among reproductive women, and we verified that women’s economic capabilities and their economic differences with men were not correlated with women’s nutritional deficiencies. Our further correlation analysis of female economic ability also verified this conclusion. The results emphasize the explanations of other factors other than economic factors for nutritional deficiencies among reproductive women. Although lower middle-income countries have serious nutritional deficiencies, a minority of countries have formulated corresponding nutrition policies, but the proportion of low-income countries that have formulated policies on malnutrition is higher than that of lower middle-income countries [21,22]. Many gaps remain in nutrition policy implementation in lower middle-income countries: policy formulation cannot be integrated with national development agendas; slow policy implementation and uneven development across regions; lack of financial support and multi-sectoral joint action, testing, inspection, and insufficient coverage of legislation and enforcement [21,23]. In addition, broader cultural and social factors continue to be a common cause of threatening nutritional deficiencies among reproductive women in lower middle-income countries. such as women’s dietary decisions in the household, education, early marriage and childbearing, etc. [24,25,26,27].

An obvious turning point in the temporal trend of nutritional deficiency incidence among reproductive women occurred in 2015, especially at the lower middle-income level, which is mainly attributable to natural disasters and conflicts [28,29]. Despite the achievement of the Millennium Development Goals from 2000 to 2015, which has made significant progress in improving women’s nutrition, global climate change after 2015 has undermined these efforts [28]. Severe climate disasters, such as the strongest El Niño phenomenon that hit Kenya, pose a great threat to local food security [30]. In addition, countries such as Nigeria, Egypt, and Tunisia received the impact of the refugee wave in 2015, food insufficiency during the migration process, and the imperfect policies and measures of various immigration countries have seriously damaged the human rights of women of childbearing age, which may also be one of the reasons for the aggravation of malnutrition [31,32].

Regionally, the age-standardized incidence rate for overall nutritional deficiencies in South Asia increased significantly, ranking first in all regions in 2019. The high incidence burden in South Asia can be attributed to the following reasons. Women are unable to make better health choices and nutritional decisions due to gender inequality, the low status of women, early marriage, unequal prenatal care [33], low education rates, low purchasing power [24], and unbalanced food supply. The high fertility rate, selective abortion, geographical concentration of the actual nutritional needs, low availability, abuse, low contraceptive use, and unmet needs for family planning are also causes of micronutrition deficiencies [34,35,36]. Heavy labor has led to excessive energy consumption. Early marriage and premature delivery can lead to pregnancy with inadequate nutritional stores and competition for dietary energy and nutrition between the fetus and pregnant mother. Simultaneously, early marriage and early childbirth hinder young women’s right to education and women’s ability to fully participate in family, social, cultural, and civic activities and prevent women from supplementing their nutritional knowledge and power, resulting in adverse effects. As a result, prioritizing investment in nutrition should be the most cost-effective intervention priority in the area and pay more attention to the use of broader ‘nutrition-sensitive’ measures that target the social determinants of health, including agricultural interventions and poverty alleviation, food security, sanitation campaigns, and women empowerment [37].

The age-standardized DALYs rate for overall nutritional deficiencies in Western Sub-Saharan Africa and high-income North America and Caribbean increased from 2010 to 2019. Poverty, climate change, habitat destruction, human immunodeficiency virus infection [20,38], parasite infection, and urbanization changes affect the food security of women of reproductive age in Western Sub-Saharan Africa. High fertility rate, low fruit and vegetable intake, high availability of cheap energy, and prevalence of bariatric surgery may contribute to the high incidence rate of nutritional deficiencies among women. Unclear classification cannot provide reliable and up-to-date data, and a limited degree of monitoring and evaluation of health information systems may weaken efforts to reduce the health damage caused by nutritional deficiencies [39].

Nationally, India exhibited the highest age-standardized incidence rate in 2019. Longitudinal data from a survey in India showed that dietary diversity was lower in girls than boys at most ages, and women have the greatest disadvantage in adolescence [40,41]. Although India’s Public Distribution System is targeted to benefit households below the poverty line, the consumption of expensive items, such as dairy, fruits, nuts, and meat, was limited to higher socioeconomic groups [42,43]. Strict religious beliefs, fasting, early marriage and childbirth, poor postpartum care, and restrictions on safe abortions all exacerbated the nutritional deficiencies of Indian women [44,45]. Over the past decade, Turkey has exhibited the steepest increase in the age-standardized incidence rate of overall nutritional deficiencies. Turkey has been undergoing socio-demographic, cultural, and economic transformations, which has led to changes in eating habits, age at first pregnancy, number of pregnancies and nutritional intake of among reproductive women. In addition, the lack of knowledge regarding nutrition in Turkish women of reproductive age also affects their food choices [46,47].

Somalia exhibited the highest age-standardized DALYs rate in 2019. The effect of extreme weather events is particularly pronounced in landlocked developing countries, small island developing states [48], and low-income food-deficit countries [49], accelerating maternal malnutrition [50]. Due to years of war and widespread drought [51,52], Somalia is facing a serious food crisis [53]. Pregnant and breastfeeding women in Somalia are considered high-priority humanitarian aid targets in emergencies due to increased nutritional needs [54], but they still cannot meet their nutritional needs. It is necessary to further explore the partnership between the government and private sector service providers to provide free basic reproductive health and nutrition services throughout Somalia and to improve technological innovation to increase the coverage of universal health insurance. It also requires humanitarian associations to continue their concerted efforts to meet the health needs of women and children [55]. Zimbabwe exhibited the steepest increase in age-standardized DALYs rate of overall nutritional deficiencies from 2010 to 2019. Poverty, repeated droughts, food shortages, reduced output of crops such as corn, low vegetable consumption, and a national human rights crisis caused by the government due to economic imbalances and inflation have exacerbated the main causes of food insecurity and nutritional deficiencies in women of reproductive age [56,57,58].

Several measures are recommended to improve nutritional deficiencies among reproductive women. First, an important role for country-level action is to strengthen technical support and food safety regulation to promote food fortification and behavior change among women of reproductive age [59,60]. Stand-alone nutrition strategies implemented within and outside the health sector, such as malaria prevention, water sanitation and hygiene promotion, and preconception care, provide important nutritional benefits [61]. In addition to policy calls, legislation has been passed to protect women’s nutrition and health during the reproductive years [62]. Second, gender equality and women’s engagement should be promoted in multisectoral programs to empower them to reach their full potential in their families, communities, and healthcare efforts [63]. Third, incorporating dietary composition and energy intake as part of women of reproductive age’s food-based dietary guidelines may be a comprehensive strategy for achieving sustainable diets [64]. Nutrition education for women should be strengthened, nutrition videos should be provided to women in poor areas, and health service coverage should be improved [42,65]. Moreover, prenatal and midwifery care for women in their reproductive years should be enhanced, and the identification of risk factors and the quality of care and health should be improved [60,66]. The Sustainable Development Goals Global Monitoring Framework increases investment in access to universal reproductive health services.

This study is the first to provide a comprehensive overview and exploration of the burden of nutritional deficiencies among reproductive women. We determined the different income level, regional and national-level burden of nutritional deficiency among reproductive women from 2010 to 2019 globally, with special attention paid to the related temporal changes and the correlation between femal economic levels and the nutritional deficiencies burden. The results of this study will help to identify gaps in nutritional deficiency burden among reproductive women and facilitate the development of regional or national responses. Compared with economic capital, macroscopic political guarantee and social and cultural capital are important measures to solve the nutritional deficiencies of reproductive women. This study has some limitations. It was a secondary data analysis of the GBD study. In this study, as with issues existing for many diseases from the GBD study, the accuracy of the results for nutritional deficiencies largely depends on the quality and quantity of input data to the models. There are no comparable studies; thus, the results of this study cannot be externally verified. In poorer regions and countries, the incidence rates of nutritional deficiencies may be underestimated due to the lack of unified assessment and monitoring tools and health personnel.

In conclusion, the burden of nutritional deficiencies in women of reproductive age remained generally stable from 2010 to 2019; however, the subcategories iodine and vitamin A deficiencies had increased incidence rates and DALYs, respectively. Age-standardized incidence rate occurring in the lower middle-income level regions showed the strongly increasing trend, such as South Asia, Southeast Asia, and Turkey, and a significantly increasing trend of age-standardized DALYs rate was observed in Western Sub-Saharan Africa and Zimbabwe. The results of this study will help to identify gaps in nutritional deficiency burden among reproductive women and facilitate the development of regional or national responses. Compared with economic capital, macroscopic political guarantees and social and cultural capital are important measures to remedy the nutritional deficiencies of reproductive women.

## 5. Conclusions

The burden of nutritional deficiencies in women of reproductive age remained generally stable from 2010 to 2019; however, the subcategories iodine and vitamin A deficiencies had increased incidence rates and DALYs, respectively. Age-standardized incidence rate occurring in the lower middle-income level regions showed the strongly increasing trend, such as South Asia, Southeast Asia, and Turkey, and a significantly increasing trend of age-standardized DALYs rate was observed in Western Sub-Saharan Africa and Zimbabwe. The results of this study will help to identify gaps in nutritional deficiency burden among reproductive women and facilitate the development of regional or national responses. Compared with economic capital, macroscopic political guarantees and social and cultural capital are important measures to remedy the nutritional deficiencies of reproductive women.

## Figures and Tables

**Table 1:** The number, age-standardized rates of incidence and DALYs in 2019 and their EAPC from 2010 to 2019 by global, SDI, World Bank Income levels, regional and national level among reproductive ages of women (15–49 years). DALYs = disability-adjusted life-years. EAPC = Estimated annual percentage changes.

	Incidence			DALYs		
	Number (Thousands)	Rate (per 100,000)	EAPC 2010–2019	Case (Thousands)	Rate (per 100,000)	EAPC 2010–2019
Global						
Nutritional deficiencies	24,656.82 (30,754.62 to 19,976.06)	1268.51 (1198.71 to 1338.32)	1.24 (−1.07 to 3.60)	9536.19 (13,424.27 to 6532.39)	490.71 (447.29 to 534.12)	−0.11 (−0.33 to 0.11)
Protein–energy malnutrition	21,202.80 (27,087.27 to 16,665.53)	1087.65 (1023.01 to 1152.29)	1.29 (−1.56 to 4.21)	898.66 (1182.66 to 667.39)	46.08 (32.77 to 59.38)	−0.19 (−1.98 to 1.64)
Iodine deficiency	3454.03 (4545.91 to 2545.38)	180.87 (154.51 to 207.22)	0.95 (0.55 to 1.35)	1068.47 (1929.70 to 586.68)	54.84 (40.33 to 69.35)	−0.68 (−0.74 to −0.62)
Vitamin A deficiency	94,135.88 (100,651.67 to 88,424.45)	4864.81 (4728.11 to 5001.52)	−3.22 (−3.34 to −3.11)	68.30 (106.04 to 40.22)	3.53 (3.46 to 3.60)	0.43 (0.26 to 0.59)
Dietary iron deficiency	-	-	−0.80 (−3.05 to 1.50)	7012.01 (10,129.94 to 4707.72)	361.15 (323.91 to 398.40)	0.06 (−0.03 to 0.16)
Other nutritional deficiency	-	-	−0.80 (−3.05 to 1.50)	488.74 (627.48 to 369.33)	25.10 (15.28 to 34.92)	−1.29 (−2.81 to 0.26)
World Bank High Income						
Nutritional deficiencies	2263.79 (2853.65 to 1780.03)	835.82 (779.16 to 892.49)	−0.52 (−1.90 to 0.88)	431.21 (616.72 to 289.18)	154.88 (130.49 to 179.27)	−0.41 (−0.73 to −0.09)
Protein–energy malnutrition	2163.73 (2761.94 to 1692.89)	795.06 (739.79 to 850.32)	−0.54 (−2.00 to 0.94)	82.96 (120.66 to 53.87)	30.21 (19.44 to 40.98)	−0.63 (−1.97 to 0.73)
Iodine deficiency	100.06 (132.36 to 74.71)	40.77 (28.25 to 53.28)	−0.16 (−0.21 to −0.11)	27.00 (53.64 to 12.26)	9.77 (9.46 to 10.09)	−0.52 (−0.59 to −0.45)
Vitamin A deficiency	2994.48 (3266.56 to 2759.72)	1136.24 (1070.17 to 1202.30)	−2.16 (−2.20 to −2.13)	0.13 (0.22 to 0.07)	0.05 (0.03 to 0.08)	3.05 (2.67 to 3.44)
Dietary iron deficiency	-	-	−0.80 (−3.05 to 1.50)	285.38 (428.75 to 180.16)	101.46 (81.72 to 121.21)	−0.22 (−0.28 to −0.15)
Other nutritional deficiency	-	-	−0.80 (−3.05 to 1.50)	35.74 (52.89 to 22.63)	13.39 (13.02 to 13.76)	−1.49 (−2.94 to −0.03)
World Bank Upper Middle Income						
Nutritional deficiencies	6783.23 (8585.43 to 5314.12)	999.49 (937.52 to 1061.45)	−0.89 (−2.92 to 1.18)	1554.03 (2260.34 to 1037.57)	226.42 (196.93 to 255.91)	−1.34 (−1.76 to −0.91)
Protein–energy malnutrition	6278.41 (8082.86 to 4807.14)	916.42 (857.09 to 975.76)	−0.80 (−3.05 to 1.50)	179.28 (251.54 to 124.70)	26.21 (16.18 to 36.25)	−0.91 (−2.84 to 1.06)
Iodine deficiency	504.82 (652.14 to 385.51)	83.06 (65.20 to 100.93)	−1.80 (−1.98 to −1.63)	159.08 (307.06 to 77.44)	22.86 (13.49 to 32.24)	−1.20 (−1.40 to −1.00)
Vitamin A deficiency	20,279.85 (22,665.53 to 18,184.43)	3101.05 (2991.91 to 3210.20)	−3.09 (−3.27 to −2.90)	12.08 (19.15 to 7.08)	1.88 (1.79 to 1.97)	0.84 (0.60 to 1.09)
Dietary iron deficiency	-	-	−0.80 (−3.05 to 1.50)	1132.50 (1663.56 to 731.91)	164.93 (139.76 to 190.10)	−1.43 (−1.66 to −1.20)
Other nutritional deficiency	-	-	−0.80 (−3.05 to 1.50)	71.08 (103.72 to 46.30)	10.54 (10.33 to 10.75)	−1.57 (−3.63 to 0.53)
World Bank Lower Middle Income						
Nutritional deficiencies	14,202.61 (17,770.82 to 11,525.36)	1698.33 (1617.56 to 1779.10)	2.90 (0.07 to 5.82)	6686.66 (9407.55 to 4588.16)	811.38 (755.55 to 867.21)	−0.57 (−0.71 to −0.43)
Protein–energy malnutrition	11,883.90 (15,337.95 to 9314.26)	1435.84 (1361.57 to 1510.11)	3.23 (−0.48 to 7.07)	457.10 (617.31 to 327.33)	55.56 (40.95 to 70.17)	0.41 (−1.90 to 2.77)
Iodine deficiency	2318.72 (3045.27 to 1696.87)	262.49 (230.74 to 294.25)	1.40 (0.67 to 2.13)	704.25 (1241.44 to 402.46)	84.99 (66.92 to 103.06)	−1.08 (−1.18 to −0.99)
Vitamin A deficiency	44,376.43 (50,881.95 to 39,128.90)	5254.74 (5112.66 to 5396.81)	−4.78 (−4.99 to −4.58)	47.71 (74.00 to 28.41)	5.68 (5.66 to 5.71)	−0.53 (−0.66 to −0.39)
Dietary iron deficiency	-	-	−0.80 (−3.05 to 1.50)	5130.27 (7309.07 to 3461.18)	623.41 (574.47 to 672.35)	−0.49 (−0.61 to −0.36)
Other nutritional deficiency	-	-	−0.80 (−3.05 to 1.50)	347.33 (446.68 to 266.45)	41.73 (29.07 to 54.39)	−1.90 (−3.35 to −0.42)
World Bank Low Income						
Nutritional deficiencies	1397.15 (1639.80 to 1171.84)	791.08 (735.95 to 846.20)	−1.17 (−2.35 to 0.03)	859.55 (1190.92 to 614.12)	534.93 (489.60 to 580.26)	−1.38 (−1.73 to −1.02)
Protein–energy malnutrition	867.44 (1043.20 to 717.15)	531.45 (486.27 to 576.64)	−0.62 (−2.26 to 1.05)	178.74 (233.10 to 137.90)	112.89 (92.07 to 133.72)	−3.17 (−4.00 to −2.34)
Iodine deficiency	529.71 (713.73 to 389.66)	259.62 (228.04 to 291.20)	−2.16 (−2.52 to −1.80)	177.87 (321.68 to 95.01)	105.27 (85.16 to 125.38)	−3.26 (−3.78 to −2.74)
Vitamin A deficiency	26,416.54 (28,010.81 to 24,803.18)	15,119.48 (14,878.47 to 15,360.48)	−3.30 (−3.40 to −3.20)	8.34 (13.00 to 4.94)	4.59 (4.33 to 4.86)	−0.84 (−1.02 to −0.67)
Dietary iron deficiency	-	-	−0.80 (−3.05 to 1.50)	460.18 (670.73 to 304.06)	290.86 (257.44 to 324.29)	0.32 (0.24 to 0.41)
Other nutritional deficiency	-	-	−0.80 (−3.05 to 1.50)	34.42 (44.81 to 25.35)	21.32 (12.27 to 30.37)	−2.38 (−2.98 to −1.78)
Central Europe, eastern Europe, and central Asia						
Central Asia						
Nutritional deficiencies	80.69 (108.01 to 59.42)	332.28 (296.56 to 368.01)	0.30 (−0.91 to 1.53)	154.18 (224.19 to 100.12)	634.65 (585.28 to 684.03)	−1.25 (−1.30 to −1.19)
Protein–energy malnutrition	74.59 (101.85 to 53.89)	306.45 (272.14 to 340.76)	0.46 (−0.85 to 1.78)	3.48 (5.17 to 2.28)	14.28 (13.08 to 15.58)	−0.49 (−1.68 to 0.70)
Iodine deficiency	6.10 (8.95 to 4.21)	25.83 (15.87 to 35.79)	−1.29 (−1.54 to −1.04)	4.39 (7.61 to 2.43)	18.07 (16.71 to 19.53)	−2.18 (−2.51 to −1.85)
Vitamin A deficiency	688.16 (775.57 to 611.62)	2825.83 (2721.64 to 2930.02)	−2.75 (−2.86 to −2.65)	0.59 (0.94 to 0.32)	2.43 (1.97 to 3.01)	0.56 (0.47 to 0.64)
Dietary iron deficiency	-	-	−0.80 (−3.05 to 1.50)	143.51 (209.36 to 92.21)	590.87 (543.23 to 638.51)	−1.21 (−1.23 to −1.19)
Other nutritional deficiency	-	-	−0.80 (−3.05 to 1.50)	2.21 (3.00 to 1.61)	9.01 (8.07 to 10.06)	−3.05 (−4.33 to −1.77)
Central Europe						
Nutritional deficiencies	150.03 (200.64 to 112.67)	583.11 (535.78 to 630.44)	0.02 (−1.36 to 1.41)	49.52 (73.85 to 31.76)	179.19 (152.95 to 205.43)	−0.63 (−0.78 to −0.47)
Protein–energy malnutrition	146.72 (197.51 to 109.45)	569.32 (522.55 to 616.08)	0.03 (−1.38 to 1.47)	5.00 (7.59 to 3.11)	19.33 (17.95 to 20.81)	−0.02 (−1.35 to 1.32)
Iodine deficiency	3.31 (4.66 to 2.28)	13.79 (12.64 to 15.06)	−0.56 (−0.67 to −0.44)	0.91 (1.80 to 0.39)	3.53 (2.97 to 4.19)	−0.63 (−0.73 to −0.52)
Vitamin A deficiency	2577.55 (2851.04 to 2350.28)	10,002.70 (9806.67 to 10,198.72)	−1.91 (−1.97 to −1.85)	-	-	0.56 (0.47 to 0.64)
Dietary iron deficiency	-	-	−0.80 (−3.05 to 1.50)	41.41 (64.40 to 26.07)	147.56 (123.75 to 171.37)	−0.68 (−0.74 to −0.62)
Other nutritional deficiency	-	-	−0.80 (−3.05 to 1.50)	2.20 (3.38 to 1.34)	8.78 (7.86 to 9.79)	−1.07 (−2.59 to 0.47)
Eastern Europe						
Nutritional deficiencies	194.87 (264.78 to 140.21)	404.50 (365.08 to 443.92)	−0.06 (−2.73 to 2.69)	86.87 (130.22 to 53.93)	161.46 (136.56 to 186.37)	−1.99 (−2.64 to −1.34)
Protein–energy malnutrition	189.64 (259.79 to 134.24)	392.73 (353.89 to 431.57)	−0.03 (−2.81 to 2.81)	9.58 (14.60 to 6.11)	19.61 (18.59 to 20.68)	0.02 (−2.28 to 2.37)
Iodine deficiency	5.23 (7.41 to 3.62)	11.77 (10.99 to 12.60)	−0.76 (−0.81 to −0.70)	4.95 (8.06 to 2.75)	10.30 (9.57 to 11.08)	−0.45 (−0.63 to −0.26)
Vitamin A deficiency	373.48 (445.21 to 310.66)	727.12 (674.27 to 779.98)	−1.89 (−2.03 to −1.76)	-	-	0.78 (0.07 to 1.49)
Dietary iron deficiency	-	-	−0.80 (−3.05 to 1.50)	67.32 (107.50 to 38.93)	121.21 (99.63 to 142.79)	−2.47 (−2.85 to −2.08)
Other nutritional deficiency	-	-	−0.80 (−3.05 to 1.50)	5.01 (7.27 to 3.34)	10.34 (9.61 to 11.12)	−0.82 (−2.79 to 1.20)
High income						
Australasia						
Nutritional deficiencies	34.14 (45.20 to 25.91)	492.67 (449.17 to 536.18)	−0.59 (−1.61 to 0.43)	6.57 (10.19 to 3.87)	94.46 (75.41 to 113.51)	0.02 (−0.49 to 0.52)
Protein–energy malnutrition	33.15 (44.26 to 24.86)	477.87 (435.02 to 520.71)	−0.61 (−1.65 to 0.44)	1.23 (1.89 to 0.76)	17.64 (15.19 to 20.49)	−0.60 (−1.59 to 0.41)
Iodine deficiency	0.99 (1.38 to 0.68)	14.81 (12.57 to 17.44)	−0.15 (−0.22 to −0.07)	0.22 (0.44 to 0.10)	3.22 (2.27 to 4.58)	−0.18 (−0.23 to −0.12)
Vitamin A deficiency	12.96 (15.77 to 10.51)	188.75 (161.82 to 215.67)	−2.20 (−2.40 to −2.00)	-	-	−0.32 (−0.68 to 0.05)
Dietary iron deficiency	-	-	−0.80 (−3.05 to 1.50)	4.57 (7.95 to 2.42)	65.59 (49.72 to 81.47)	0.37 (−0.06 to 0.80)
Other nutritional deficiency	-	-	−0.80 (−3.05 to 1.50)	0.55 (0.85 to 0.34)	8.00 (6.41 to 9.99)	−1.44 (−2.49 to −0.39)
High-income Asia Pacific						
Nutritional deficiencies	202.71 (266.27 to 154.92)	506.16 (462.07 to 550.26)	−0.66 (−2.43 to 1.13)	104.42 (156.45 to 64.36)	250.66 (219.63 to 281.69)	−0.90 (−1.19 to −0.60)
Protein–energy malnutrition	196.04 (258.08 to 148.04)	488.48 (445.16 to 531.80)	−0.68 (−2.52 to 1.18)	6.35 (9.27 to 4.29)	15.47 (14.47 to 16.54)	−0.89 (−2.50 to 0.75)
Iodine deficiency	6.68 (9.31 to 4.68)	17.68 (16.61 to 18.83)	−0.22 (−0.32 to −0.13)	1.60 (3.08 to 0.70)	3.97 (3.48 to 4.53)	−0.33 (−0.43 to −0.22)
Vitamin A deficiency	278.03 (339.23 to 226.57)	760.47 (706.42 to 814.52)	−1.71 (−1.76 to −1.66)	-	-	3.02 (2.34 to 3.70)
Dietary iron deficiency	-	-	−0.80 (−3.05 to 1.50)	93.90 (142.08 to 56.06)	224.61 (195.24 to 253.99)	−0.89 (−1.07 to −0.71)
Other nutritional deficiency	-	-	−0.80 (−3.05 to 1.50)	2.58 (3.89 to 1.63)	6.61 (5.96 to 7.32)	−1.62 (−3.42 to 0.22)
High-income North America						
Nutritional deficiencies	582.77 (750.89 to 452.14)	694.02 (642.39 to 745.66)	−0.43 (−1.94 to 1.10)	114.81 (176.21 to 70.60)	134.45 (111.72 to 157.18)	1.03 (0.80 to 1.26)
Protein–energy malnutrition	569.34 (737.83 to 438.49)	677.60 (626.58 to 728.62)	−0.44 (−1.99 to 1.13)	16.22 (23.84 to 10.76)	19.07 (18.30 to 19.88)	−0.18 (−1.37 to 1.02)
Iodine deficiency	13.43 (18.91 to 9.36)	16.42 (16.15 to 16.70)	−0.19 (−0.29 to −0.09)	3.03 (5.88 to 1.35)	3.61 (3.29 to 3.97)	−0.09 (−0.13 to −0.05)
Vitamin A deficiency	500.71 (625.99 to 386.74)	614.78 (566.18 to 663.37)	−1.47 (−1.68 to −1.27)	-	-	3.02 (2.34 to 3.70)
Dietary iron deficiency	-	-	−0.80 (−3.05 to 1.50)	89.11 (144.92 to 50.50)	104.01 (84.02 to 124.00)	1.46 (0.96 to 1.96)
Other nutritional deficiency	-	-	−0.80 (−3.05 to 1.50)	6.45 (9.85 to 3.93)	7.75 (7.27 to 8.27)	−1.21 (−2.73 to 0.34)
Southern Latin America						
Nutritional deficiencies	109.41 (140.33 to 84.84)	632.36 (583.07 to 681.64)	−0.43 (−2.11 to 1.28)	25.68 (40.45 to 15.30)	147.70 (123.88 to 171.52)	−1.22 (−1.29 to −1.14)
Protein–energy malnutrition	108.09 (138.83 to 83.35)	624.58 (575.60 to 673.57)	−0.43 (−2.13 to 1.30)	3.04 (3.84 to 2.38)	17.55 (15.98 to 19.29)	−1.54 (−2.45 to −0.61)
Iodine deficiency	1.32 (1.85 to 0.89)	7.77 (6.75 to 8.95)	−0.47 (−0.68 to −0.26)	0.36 (0.69 to 0.15)	2.07 (1.58 to 2.73)	−0.48 (−0.65 to −0.30)
Vitamin A deficiency	854.74 (1045.00 to 693.74)	5001.32 (4862.71 to 5139.93)	−2.22 (−2.33 to −2.11)	-	-	0.60 (0.35 to 0.84)
Dietary iron deficiency	-	-	−0.80 (−3.05 to 1.50)	21.39 (35.91 to 11.74)	122.94 (101.21 to 144.67)	−1.18 (−1.27 to −1.09)
Other nutritional deficiency	-	-	−0.80 (−3.05 to 1.50)	0.89 (1.25 to 0.58)	5.13 (4.31 to 6.11)	−1.31 (−2.16 to −0.46)
Western Europe						
Nutritional deficiencies	1128.70 (1412.23 to 907.35)	1185.47 (1117.98 to 1252.95)	−0.56 (−1.84 to 0.73)	128.52 (181.50 to 85.05)	131.51 (109.03 to 153.98)	−0.74 (−1.52 to 0.05)
Protein–energy malnutrition	1055.25 (1338.89 to 830.61)	1097.27 (1032.35 to 1162.20)	−0.61 (−2.00 to 0.80)	48.51 (71.72 to 30.34)	50.26 (36.36 to 64.15)	−0.53 (−1.91 to 0.86)
Iodine deficiency	73.45 (96.94 to 53.94)	88.19 (69.79 to 106.60)	−0.06 (−0.17 to 0.04)	20.62 (41.12 to 9.44)	21.04 (12.05 to 30.03)	−0.25 (−0.36 to −0.14)
Vitamin A deficiency	444.64 (492.77 to 400.30)	495.08 (451.47 to 538.69)	−1.53 (−1.57 to −1.48)	-	-	−0.83 (−1.27 to −0.39)
Dietary iron deficiency	-	-	−0.80 (−3.05 to 1.50)	37.80 (59.84 to 22.59)	37.19 (25.23 to 49.14)	−0.89 (−1.08 to −0.69)
Other nutritional deficiency	-	-	−0.80 (−3.05 to 1.50)	21.58 (32.01 to 13.66)	23.03 (13.62 to 32.43)	−1.40 (−2.82 to 0.03)
Latin America and Caribbean						
Andean Latin America						
Nutritional deficiencies	77.85 (91.20 to 65.67)	472.84 (430.22 to 515.46)	−0.52 (−1.79 to 0.77)	37.75 (52.82 to 26.61)	230.90 (201.12 to 260.68)	−2.49 (−3.37 to −1.59)
Protein–energy malnutrition	76.92 (90.25 to 64.55)	467.29 (424.92 to 509.66)	−0.52 (−1.80 to 0.78)	5.52 (7.06 to 4.23)	33.63 (22.27 to 45.00)	−3.63 (−4.92 to −2.32)
Iodine deficiency	0.92 (1.31 to 0.60)	5.54 (4.68 to 6.57)	−0.80 (−1.09 to −0.51)	0.23 (0.44 to 0.11)	1.39 (0.99 to 1.95)	−1.03 (−1.25 to −0.80)
Vitamin A deficiency	931.86 (1081.04 to 799.25)	5601.79 (5455.09 to 5748.48)	−2.91 (−3.23 to −2.59)	0.57 (0.96 to 0.31)	3.44 (2.77 to 4.26)	−0.32 (−0.68 to 0.05)
Dietary iron deficiency	-	-	−0.80 (−3.05 to 1.50)	29.60 (43.74 to 19.16)	181.40 (155.00 to 207.80)	−2.27 (−3.07 to −1.46)
Other nutritional deficiency	-	-	−0.80 (−3.05 to 1.50)	1.82 (2.34 to 1.41)	11.03 (9.78 to 12.44)	−3.19 (−4.42 to −1.94)
Caribbean						
Nutritional deficiencies	60.67 (75.01 to 48.96)	502.10 (458.18 to 546.02)	−0.54 (−1.90 to 0.84)	58.37 (81.42 to 39.98)	484.77 (441.62 to 527.93)	0.44 (0.32 to 0.55)
Protein–energy malnutrition	57.49 (71.38 to 46.00)	475.58 (432.83 to 518.32)	−0.50 (−1.93 to 0.96)	6.15 (8.08 to 4.73)	50.97 (36.97 to 64.96)	0.45 (0.18 to 0.73)
Iodine deficiency	3.19 (4.95 to 2.01)	26.52 (16.43 to 36.62)	−1.33 (−1.46 to −1.20)	1.95 (3.22 to 1.07)	16.20 (13.69 to 19.18)	−1.44 (−1.81 to −1.06)
Vitamin A deficiency	693.74 (815.07 to 591.35)	5753.87 (5605.19 to 5902.54)	−1.33 (−1.38 to −1.29)	0.27 (0.44 to 0.15)	2.24 (2.18 to 2.31)	0.45 (0.20 to 0.70)
Dietary iron deficiency	-	-	−0.80 (−3.05 to 1.50)	48.57 (70.86 to 31.93)	403.63 (364.26 to 443.01)	0.56 (0.46 to 0.67)
Other nutritional deficiency	-	-	−0.80 (−3.05 to 1.50)	1.42 (1.89 to 1.02)	11.73 (11.38 to 12.09)	−1.15 (−1.98 to −0.31)
Central Latin America						
Nutritional deficiencies	601.18 (755.83 to 476.51)	892.47 (833.92 to 951.02)	−0.29 (−1.50 to 0.93)	135.41 (184.08 to 98.03)	201.45 (173.63 to 229.26)	−0.64 (−0.84 to −0.44)
Protein–energy malnutrition	581.61 (737.17 to 457.42)	863.50 (805.90 to 921.09)	−0.29 (−1.54 to 0.97)	38.00 (46.57 to 30.72)	56.45 (41.72 to 71.18)	−1.50 (−2.42 to −0.58)
Iodine deficiency	19.57 (27.82 to 13.58)	28.98 (18.42 to 39.53)	−0.26 (−0.38 to −0.14)	11.63 (20.61 to 6.12)	17.23 (16.42 to 18.07)	2.95 (2.04 to 3.86)
Vitamin A deficiency	2780.59 (3229.48 to 2401.76)	4118.73 (3992.94 to 4244.51)	−2.15 (−2.21 to −2.09)	2.83 (4.57 to 1.61)	4.19 (3.80 to 4.62)	0.62 (0.24 to 1.01)
Dietary iron deficiency	-	-	−0.80 (−3.05 to 1.50)	72.75 (107.56 to 47.15)	108.48 (88.07 to 128.90)	−0.55 (−0.82 to −0.28)
Other nutritional deficiency	-	-	−0.80 (−3.05 to 1.50)	10.19 (13.98 to 6.99)	15.10 (14.35 to 15.89)	−1.83 (−2.78 to −0.88)
Tropical Latin America						
Nutritional deficiencies	263.21 (339.32 to 204.06)	432.69 (391.92 to 473.46)	−1.47 (−4.46 to 1.60)	321.33 (478.00 to 204.87)	530.60 (485.46 to 575.75)	−0.88 (−0.99 to −0.76)
Protein–energy malnutrition	258.81 (334.93 to 200.68)	425.31 (384.89 to 465.73)	−1.50 (−4.54 to 1.64)	17.27 (21.71 to 13.89)	28.17 (17.76 to 38.57)	−2.42 (−4.19 to −0.62)
Iodine deficiency	4.40 (6.20 to 2.96)	7.38 (6.83 to 7.98)	−0.22 (−0.29 to −0.15)	0.99 (1.96 to 0.42)	1.62 (1.38 to 1.92)	−0.06 (−0.20 to 0.08)
Vitamin A deficiency	5366.23 (6711.12 to 4222.32)	8920.74 (8735.62 to 9105.86)	−2.52 (−2.75 to −2.30)	1.79 (2.95 to 1.00)	3.06 (2.71 to 3.45)	−0.83 (−1.27 to −0.39)
Dietary iron deficiency	-	-	−0.80 (−3.05 to 1.50)	296.31 (449.62 to 183.74)	489.56 (446.19 to 532.92)	−0.76 (−0.86 to −0.66)
Other nutritional deficiency	-	-	−0.80 (−3.05 to 1.50)	4.98 (7.05 to 3.36)	8.19 (7.61 to 8.82)	−2.91 (−5.31 to −0.45)
North Africa and Middle East						
Nutritional deficiencies	1230.54 (1558.04 to 982.69)	776.75 (722.12 to 831.38)	0.04 (−1.78 to 1.89)	463.71 (672.61 to 314.55)	294.94 (261.28 to 328.60)	−0.60 (−0.72 to −0.47)
Protein–energy malnutrition	1168.57 (1496.94 to 917.16)	737.69 (684.46 to 790.93)	0.06 (−1.85 to 2.01)	44.98 (60.36 to 32.55)	28.46 (18.00 to 38.92)	−1.72 (−3.07 to −0.34)
Iodine deficiency	61.96 (87.60 to 45.45)	39.06 (26.81 to 51.31)	−0.40 (−0.66 to −0.15)	51.52 (81.47 to 30.45)	32.45 (21.29 to 43.62)	−1.21 (−1.82 to −0.59)
Vitamin A deficiency	5555.79 (6089.40 to 5074.35)	3495.45 (3379.57 to 3611.33)	−3.53 (−3.69 to −3.38)	3.40 (5.45 to 1.94)	2.13 (1.95 to 2.33)	0.41 (0.21 to 0.61)
Dietary iron deficiency	-	-	−0.80 (−3.05 to 1.50)	348.16 (520.74 to 224.66)	222.06 (192.85 to 251.26)	−0.35 (−0.52 to −0.18)
Other nutritional deficiency	-	-	−0.80 (−3.05 to 1.50)	15.66 (22.49 to 10.35)	9.84 (9.44 to 10.25)	−1.15 (−2.86 to 0.59)
South Asia						
Nutritional deficiencies	10,360.11 (12,971.59 to 8360.87)	2149.96 (2059.08 to 2240.84)	3.98 (0.49 to 7.59)	5201.15 (7339.60 to 3559.88)	1102.44 (1037.36 to 1167.52)	−0.81 (−1.04 to −0.57)
Protein–energy malnutrition	8339.27 (10,932.74 to 6442.87)	1752.37 (1670.32 to 1834.42)	4.63 (−0.34 to 9.84)	287.66 (404.47 to 193.24)	60.73 (45.46 to 76.01)	1.80 (−1.77 to 5.50)
Iodine deficiency	2020.84 (2669.04 to 1472.70)	397.59 (358.51 to 436.67)	1.70 (0.82 to 2.58)	596.18 (1055.62 to 336.34)	125.76 (103.78 to 147.74)	−1.38 (−1.43 to −1.32)
Vitamin A deficiency	24,740.08 (30,932.53 to 19,771.02)	5132.57 (4992.15 to 5272.99)	−5.51 (−5.83 to −5.19)	30.04 (46.51 to 17.80)	6.28 (6.25 to 6.31)	−0.62 (−0.88 to −0.37)
Dietary iron deficiency	-	-	−0.80 (−3.05 to 1.50)	3998.18 (5680.15 to 2692.66)	849.05 (791.94 to 906.16)	−0.78 (−0.87 to −0.69)
Other nutritional deficiency	-	-	−0.80 (−3.05 to 1.50)	289.10 (369.87 to 216.35)	60.62 (45.36 to 75.88)	−2.18 (−3.77 to −0.57)
Southeast Asia, east Asia, and Oceania						
East Asia						
Nutritional deficiencies	4758.43 (6070.60 to 3684.63)	1305.54 (1234.72 to 1376.35)	−0.81 (−3.01 to 1.44)	672.38 (1009.75 to 427.61)	175.79 (149.80 to 201.77)	−2.21 (−2.89 to −1.53)
Protein–energy malnutrition	4333.72 (5627.35 to 3254.45)	1166.60 (1099.65 to 1233.54)	−0.78 (−3.29 to 1.80)	88.64 (137.84 to 53.79)	23.77 (14.21 to 33.32)	−0.93 (−3.48 to 1.67)
Iodine deficiency	424.71 (551.62 to 319.01)	138.94 (115.84 to 162.04)	−1.12 (−1.34 to −0.89)	120.07 (238.30 to 54.74)	31.05 (20.13 to 41.97)	−1.16 (−1.36 to −0.96)
Vitamin A deficiency	6862.84 (8902.78 to 5298.75)	1972.29 (1885.24 to 2059.33)	−4.80 (−5.34 to −4.26)	5.10 (8.40 to 2.99)	1.43 (1.33 to 1.54)	0.78 (0.07 to 1.49)
Dietary iron deficiency	-	-	−0.80 (−3.05 to 1.50)	418.15 (632.14 to 257.45)	108.42 (88.01 to 128.83)	−2.81 (−3.21 to −2.42)
Other nutritional deficiency	-	-	−0.80 (−3.05 to 1.50)	40.42 (62.13 to 24.83)	11.12 (10.83 to 11.41)	−1.69 (−4.20 to 0.89)
Oceania						
Nutritional deficiencies	23.63 (28.43 to 19.33)	726.27 (673.45 to 779.09)	−2.13 (−3.96 to −0.27)	17.48 (25.67 to 11.37)	539.32 (493.80 to 584.84)	−0.64 (−0.78 to −0.49)
Protein–energy malnutrition	23.41 (28.21 to 19.10)	719.75 (667.17 to 772.33)	−2.13 (−3.97 to −0.26)	1.12 (1.59 to 0.78)	35.72 (24.00 to 47.43)	−2.65 (−3.28 to −2.01)
Iodine deficiency	0.22 (0.31 to 0.15)	6.52 (4.61 to 9.23)	−2.57 (−3.03 to −2.09)	0.10 (0.18 to 0.05)	3.13 (1.90 to 5.17)	−5.80 (−6.73 to −4.88)
Vitamin A deficiency	308.74 (378.54 to 254.10)	9047.62 (8861.18 to 9234.05)	−2.21 (−2.31 to −2.12)	0.06 (0.11 to 0.03)	1.92 (1.01 to 3.64)	−0.58 (−0.71 to −0.45)
Dietary iron deficiency	-	-	−0.80 (−3.05 to 1.50)	15.95 (24.09 to 10.00)	491.22 (447.78 to 534.66)	−0.41 (−0.49 to −0.32)
Other nutritional deficiency	-	-	−0.80 (−3.05 to 1.50)	0.24 (0.35 to 0.16)	7.34 (5.29 to 10.18)	−2.52 (−4.15 to −0.86)
Southeast Asia						
Nutritional deficiencies	2969.02 (3649.39 to 2412.23)	1647.75 (1568.19 to 1727.31)	1.37 (0.07 to 2.68)	727.62 (1036.84 to 495.32)	403.48 (364.11 to 442.85)	−0.49 (−0.55 to −0.43)
Protein–energy malnutrition	2821.83 (3490.72 to 2267.59)	1564.13 (1486.61 to 1641.65)	1.61 (0.26 to 2.98)	104.56 (136.06 to 76.77)	57.83 (42.93 to 72.74)	−0.05 (−0.67 to 0.57)
Iodine deficiency	147.19 (191.84 to 108.75)	83.62 (65.69 to 101.54)	−2.31 (−2.63 to −1.99)	40.69 (70.34 to 22.58)	22.56 (13.25 to 31.87)	1.69 (−0.05 to 3.46)
Vitamin A deficiency	6697.42 (7864.25 to 5729.26)	3767.90 (3647.59 to 3888.21)	−4.25 (−4.47 to −4.03)	9.07 (14.44 to 5.32)	5.07 (4.81 to 5.36)	−0.68 (−0.84 to −0.53)
Dietary iron deficiency	-	-	−0.80 (−3.05 to 1.50)	533.11 (787.71 to 341.09)	295.66 (261.96 to 329.36)	−0.80 (−0.92 to −0.68)
Other nutritional deficiency	-	-	−0.80 (−3.05 to 1.50)	40.19 (54.94 to 27.92)	22.35 (13.08 to 31.62)	0.75 (0.01 to 1.50)
Sub-Saharan Africa						
Central sub-Saharan Africa						
Nutritional deficiencies	308.43 (392.50 to 242.03)	922.87 (863.33 to 982.41)	−1.28 (−2.71 to 0.16)	186.04 (291.30 to 118.94)	629.61 (580.43 to 678.79)	−2.21 (−2.56 to −1.86)
Protein–energy malnutrition	145.80 (177.11 to 118.58)	492.28 (448.79 to 535.77)	−1.58 (−3.81 to 0.69)	25.84 (40.73 to 16.60)	92.30 (73.47 to 111.13)	−5.23 (−5.60 to −4.85)
Iodine deficiency	162.63 (242.02 to 103.08)	430.59 (389.92 to 471.26)	−0.96 (−1.53 to −0.38)	81.93 (155.87 to 40.62)	263.81 (231.97 to 295.64)	−2.61 (−3.15 to −2.07)
Vitamin A deficiency	4192.16 (5086.42 to 3447.77)	12,995.76 (12,772.33 to 13,219.20)	−5.80 (−6.11 to −5.48)	2.03 (3.25 to 1.17)	6.41 (5.72 to 7.19)	−1.21 (−1.52 to −0.91)
Dietary iron deficiency	-	-	−0.80 (−3.05 to 1.50)	71.20 (106.91 to 45.31)	249.28 (218.33 to 280.22)	−0.09 (−0.12 to −0.05)
Other nutritional deficiency	-	-	−0.80 (−3.05 to 1.50)	5.04 (7.30 to 2.99)	17.82 (16.64 to 19.07)	−4.90 (−5.69 to −4.11)
Eastern sub-Saharan Africa						
Nutritional deficiencies	828.88 (973.70 to 694.57)	787.71 (732.70 to 842.72)	−1.43 (−2.58 to −0.26)	455.95 (614.50 to 333.16)	492.43 (448.94 to 535.93)	−1.93 (−2.29 to −1.56)
Protein–energy malnutrition	479.99 (583.03 to 389.55)	500.10 (456.27 to 543.93)	−0.51 (−2.23 to 1.23)	121.00 (151.97 to 95.12)	131.34 (108.88 to 153.81)	−4.06 (−4.91 to −3.20)
Iodine deficiency	348.89 (459.68 to 256.63)	287.61 (254.37 to 320.85)	−2.78 (−3.12 to −2.43)	87.94 (160.10 to 47.24)	90.35 (71.72 to 108.98)	−4.19 (−4.73 to −3.65)
Vitamin A deficiency	19,219.85 (20,886.08 to 17,757.87)	18,955.34 (18,685.49 to 19,225.19)	−3.19 (−3.24 to −3.15)	5.25 (8.08 to 3.12)	4.87 (4.53 to 5.23)	−1.17 (−1.38 to −0.95)
Dietary iron deficiency	-	-	−0.80 (−3.05 to 1.50)	221.78 (321.81 to 148.16)	244.31 (213.67 to 274.94)	0.71 (0.57 to 0.85)
Other nutritional deficiency	-	-	−0.80 (−3.05 to 1.50)	19.99 (27.72 to 14.77)	21.57 (12.47 to 30.67)	−3.48 (−4.08 to −2.86)
Southern sub-Saharan Africa						
Nutritional deficiencies	91.96 (111.16 to 74.78)	434.25 (393.41 to 475.10)	−0.30 (−1.50 to 0.92)	80.71 (111.69 to 56.51)	397.90 (358.80 to 436.99)	−1.00 (−1.45 to −0.54)
Protein–energy malnutrition	76.32 (94.35 to 60.33)	362.16 (324.86 to 399.46)	−0.38 (−1.84 to 1.11)	11.98 (15.35 to 9.41)	58.09 (43.15 to 73.03)	−3.90 (−5.56 to −2.20)
Iodine deficiency	15.64 (21.59 to 10.68)	72.09 (55.45 to 88.74)	0.03 (−0.18 to 0.24)	3.89 (7.61 to 1.82)	18.28 (16.84 to 19.85)	−0.48 (−0.61 to −0.34)
Vitamin A deficiency	1278.53 (1479.71 to 1089.51)	5942.55 (5791.46 to 6093.64)	−2.52 (−2.60 to −2.44)	1.00 (1.56 to 0.58)	4.71 (4.01 to 5.54)	−0.18 (−0.71 to 0.35)
Dietary iron deficiency	-	-	−0.80 (−3.05 to 1.50)	62.24 (89.63 to 40.29)	309.25 (274.78 to 343.71)	−0.39 (−0.58 to −0.20)
Other nutritional deficiency	-	-	−0.80 (−3.05 to 1.50)	1.60 (2.15 to 1.14)	7.57 (6.66 to 8.60)	−2.20 (−3.64 to −0.74)
Western sub-Saharan Africa						
Nutritional deficiencies	599.61 (734.87 to 486.08)	537.83 (492.37 to 583.28)	−1.52 (−3.41 to 0.41)	507.73 (719.23 to 344.56)	467.36 (424.98 to 509.73)	1.71 (1.24 to 2.18)
Protein–energy malnutrition	466.24 (587.04 to 368.98)	431.29 (390.58 to 471.99)	−1.63 (−4.01 to 0.81)	52.54 (70.52 to 37.95)	50.36 (36.45 to 64.27)	−0.58 (−1.54 to 0.39)
Iodine deficiency	133.36 (185.55 to 91.08)	106.54 (86.31 to 126.77)	−1.15 (−1.28 to −1.03)	35.27 (63.50 to 18.92)	32.64 (21.44 to 43.84)	−1.79 (−2.02 to −1.56)
Vitamin A deficiency	9777.79 (10,821.33 to 8785.81)	8416.14 (8236.33 to 8595.95)	−4.26 (−4.35 to −4.17)	6.30 (9.86 to 3.64)	5.20 (4.87 to 5.56)	−0.44 (−0.75 to −0.12)
Dietary iron deficiency	-	-	−0.80 (−3.05 to 1.50)	397.00 (578.98 to 260.21)	364.08 (326.69 to 401.48)	2.66 (1.88 to 3.43)
Other nutritional deficiency	-	-	−0.80 (−3.05 to 1.50)	16.62 (22.46 to 12.07)	15.07 (14.49 to 15.67)	−1.45 (−2.21 to −0.69)

Note: The “-” and “0.00 (0.00 to 0.00)” in the supplemental file indicate “no data” and “data available but no deficiency occurred”, respectively.

## Data Availability

The data underlying this article are available on the Global Health Data Exchange at http://ghdx.healthdata.org/ihmedata (accessed on 16 December 2020).

## Supplementary Materials

The following supporting information can be downloaded at: https://www.mdpi.com/article/10.3390/nu14040832/s1, Table S1: Age-standardized rates in 2019, and their EAPC from 2010 to 2019 of incidence and DALYs in global 204 countries for reproductive ages of women (15–49 years). DALYs = disability-adjusted life-years. EAPC = Estimated annual percentage changes.
