# Supplementary material for: Global, Regional, and National Estimates of Nutritional Deficiency Burden among Reproductive Women from 2010 to 2019
Source: Nutrients. 2022 Feb 16;14(4):832. doi: 10.3390/nu14040832 (PMC8877546; doi:10.3390/nu14040832)
Supplement: Supplementary file 1 [file nutrients-14-00832-s001.zip › nutrients-1552233-supplementary.pdf]

Supplementary Materials

Table S1. Age-standardized rates in 2019, and their EAPC from 2010 to 2019 of incidence and DALYs in global 204 countries for reproductive ages of women (15-49 years). DALYs=disability-adjusted life-years. EAPC=Estimated annual percentage changes.

	Incidence		DALYs	
	Rate (per 100,000)	EAPC 2010-2019	Rate (per 100,000)	EAPC 2010-2019
**Afghanistan**				
Nutritional deficiencies	406.24(366.73to445.74)	-1.33(-1.88to-0.77)	344.82(308.42to381.22)	-4.05(-4.35to-3.75)
Protein-energy malnutrition	352.84(316.03to389.66)	-1.25(-1.82to-0.68)	114.90(93.89to135.91)	-5.79(-6.37to-5.21)
Iodine deficiency	53.39(39.07to67.72)	-1.78(-2.22to-1.33)	76.50(59.36to93.64)	-2.19(-2.63to-1.75)
Vitamin A deficiency	14448.36(14212.77to14683.95)	-3.17(-3.55to-2.79)	3.60(2.70to4.78)	-0.72(-0.86to-0.57)
Dietary iron deficiency	-	-	139.99(116.80to163.18)	-3.18(-3.32to-3.03)
Other nutritional deficiency	-	-	9.84(8.28to11.70)	0.68(-0.88to2.26)
**Albania**				
Nutritional deficiencies	1458.41(1383.56to1533.26)	3.28(2.80to3.77)	312.89(278.22to347.56)	-1.31(-1.39to-1.24)
Protein-energy malnutrition	1444.86(1370.36to1519.36)	3.36(2.86to3.87)	50.35(36.44to64.26)	3.45(3.03to3.88)
Iodine deficiency	13.55(7.77to23.63)	-2.34(-2.77to-1.92)	3.41(1.13to10.34)	-11.57(-14.97to-8.05)
Vitamin A deficiency	15727.92(15482.11to15973.72)	-3.90(-4.19to-3.60)	0.00(0.00to0.00)	-
Dietary iron deficiency	-	-	235.46(205.39to265.54)	-2.37(-2.42to-2.32)
Other nutritional deficiency	-	-	23.66(14.13to33.20)	6.94(4.53to9.40)
**Algeria**				
Nutritional deficiencies	753.41(699.61to807.20)	0.85(0.66to1.03)	255.32(224.00to286.64)	-1.81(-1.90to-1.72)
Protein-energy malnutrition	722.53(669.85to775.22)	0.99(0.80to1.17)	27.68(17.36to37.99)	-1.64(-1.82to-1.46)
Iodine deficiency	30.87(19.98to41.77)	-1.76(-1.89to-1.62)	18.85(16.84to21.09)	-3.57(-3.85to-3.29)
Vitamin A deficiency	1911.48(1825.79to1997.17)	-4.33(-4.49to-4.17)	3.90(3.04to5.00)	-0.65(-0.74to-0.56)
Dietary iron deficiency	-	-	192.56(165.36to219.76)	-1.91(-2.01to-1.80)
Other nutritional deficiency	-	-	12.34(10.73to14.18)	3.50(1.64to5.41)
**American Samoa**				
Nutritional deficiencies	1090.61(1025.88to1155.34)	-0.55(-0.95to-0.15)	501.58(457.68to545.47)	-0.16(-0.51to0.20)
Protein-energy malnutrition	1086.67(1022.06to1151.28)	-0.55(-0.95to-0.15)	42.24(29.50to54.98)	-2.27(-2.51to-2.03)
Iodine deficiency	3.94(0.00to4217.54)	-1.23(-1.34to-1.11)	0.70(0.00to11355843.60)	-1.37(-1.48to-1.26)
Vitamin A deficiency	3728.49(3608.81to3848.17)	-2.36(-3.07to-1.65)	0.00(0.00to0.00)	-
Dietary iron deficiency	-	-	446.38(404.97to487.79)	-0.04(-0.38to0.31)
Other nutritional deficiency	-	-	12.26(0.23to640.49)	3.01(0.68to5.40)

**Andorra**				
Nutritional deficiencies	887.11(828.74to945.49)	0.64(0.32to0.97)	97.97(78.57to117.37)	0.29(-0.14to0.73)
Protein-energy malnutrition	825.60(769.29to881.92)	0.68(0.34to1.03)	36.76(24.88to48.65)	0.85(0.51to1.19)
Iodine deficiency	61.51(46.14to76.88)	0.05(0.01to0.08)	13.91(0.59to327.53)	0.02(0.01to0.03)
Vitamin A deficiency	329.94(294.34to365.55)	-1.05(-1.14to-0.97)	0.00(0.00to0.00)	-
Dietary iron deficiency	-	-	30.35(19.55to41.14)	-1.78(-1.93to-1.62)
Other nutritional deficiency	-	-	16.95(0.97to296.52)	4.20(1.90to6.54)
**Angola**				
Nutritional deficiencies	644.77(595.00to694.53)	-2.77(-2.96to-2.57)	454.85(413.05to496.65)	-4.27(-4.67to-3.86)
Protein-energy malnutrition	332.38(296.64to368.11)	-0.53(-0.78to-0.28)	89.70(71.14to108.26)	-5.17(-5.80to-4.53)
Iodine deficiency	312.39(277.75to347.03)	-4.50(-5.08to-3.92)	152.65(128.44to176.87)	-5.86(-6.62to-5.09)
Vitamin A deficiency	7852.50(7678.81to8026.18)	-3.49(-4.04to-2.93)	7.54(6.05to9.40)	-1.84(-1.95to-1.72)
Dietary iron deficiency	-	-	190.04(163.02to217.06)	-1.06(-1.13to-0.98)
Other nutritional deficiency	-	-	14.92(12.76to17.45)	-6.44(-6.98to-5.90)
**Antigua and Barbuda**				
Nutritional deficiencies	701.58(649.66to753.49)	-0.95(-1.02to-0.88)	388.53(349.90to427.16)	-1.30(-1.41to-1.20)
Protein-energy malnutrition	690.36(638.86to741.86)	-0.90(-0.97to-0.83)	34.72(23.18to46.27)	-2.44(-2.62to-2.26)
Iodine deficiency	11.22(0.49to256.77)	-2.99(-3.37to-2.62)	5.15(0.05to523.26)	-3.02(-3.23to-2.80)
Vitamin A deficiency	2236.43(2143.74to2329.12)	-3.40(-3.54to-3.26)	2.92(0.01to1350.65)	0.40(0.28to0.52)
Dietary iron deficiency	-	-	336.22(300.28to372.16)	-1.24(-1.33to-1.15)
Other nutritional deficiency	-	-	9.52(0.32to284.78)	2.27(0.26to4.32)
**Argentina**				
Nutritional deficiencies	679.95(628.84to731.05)	0.05(-0.05to0.14)	174.54(148.64to200.43)	-0.63(-0.70to-0.57)
Protein-energy malnutrition	672.19(621.37to723.00)	0.05(-0.04to0.15)	15.03(13.29to17.00)	-2.69(-3.34to-2.05)
Iodine deficiency	7.76(6.54to9.21)	-0.32(-0.35to-0.30)	2.07(1.48to2.88)	-0.38(-0.49to-0.27)
Vitamin A deficiency	5903.77(5753.17to6054.37)	-0.59(-1.26to0.09)	0.00(0.00to0.00)	-
Dietary iron deficiency	-	-	153.93(129.61to178.25)	-0.42(-0.47to-0.36)
Other nutritional deficiency	-	-	3.50(2.72to4.52)	-0.42(-1.35to0.51)
**Armenia**				
Nutritional deficiencies	370.40(332.68to408.12)	-2.47(-2.81to-2.13)	214.31(185.62to243.00)	-3.55(-3.96to-3.14)
Protein-energy malnutrition	331.19(295.52to366.86)	-1.52(-1.62to-1.42)	10.10(5.57to18.33)	-13.17(-15.30to-10.99)
Iodine deficiency	39.21(26.94to51.48)	-7.91(-10.05to-5.72)	28.70(18.20to39.20)	-7.51(-9.72to-5.23)
Vitamin A deficiency	550.95(504.95to596.96)	-4.47(-6.10to-2.81)	0.00(0.00to0.00)	-
Dietary iron deficiency	-	-	169.16(143.66to194.65)	-0.21(-0.83to0.40)
Other nutritional deficiency	-	-	6.36(3.00to13.47)	-18.82(-21.27to-

				16.29)
**Australia**				
Nutritional deficiencies	479.47(436.55to522.39)	1.28(1.09to1.47)	96.46(77.21to115.71)	-0.95(-1.18to-0.72)
Protein-energy malnutrition	465.03(422.76to507.29)	1.33(1.14to1.52)	17.26(14.64to20.34)	1.15(0.93to1.36)
Iodine deficiency	14.44(12.07to17.29)	-0.29(-0.35to-0.24)	3.13(2.13to4.61)	-0.42(-0.46to-0.38)
Vitamin A deficiency	105.83(85.67to126.00)	-0.56(-0.89to-0.22)	0.00(0.00to0.00)	-
Dietary iron deficiency	-	-	68.30(52.10to84.49)	-1.93(-2.18to-1.67)
Other nutritional deficiency	-	-	7.77(6.08to9.92)	4.48(2.56to6.44)
**Austria**				
Nutritional deficiencies	988.42(926.80to1050.04)	0.60(0.46to0.73)	104.14(84.14to124.14)	0.70(0.39to1.01)
Protein-energy malnutrition	924.95(865.34to984.56)	0.63(0.49to0.78)	41.05(28.49to53.61)	0.87(0.72to1.02)
Iodine deficiency	63.47(47.86to79.09)	0.08(0.05to0.11)	14.44(10.61to19.65)	0.04(0.00to0.09)
Vitamin A deficiency	446.01(404.62to487.40)	-1.54(-1.68to-1.40)	0.00(0.00to0.00)	-
Dietary iron deficiency	-	-	29.64(18.97to40.31)	-0.85(-1.61to-0.09)
Other nutritional deficiency	-	-	19.01(14.53to24.87)	4.33(2.28to6.43)
**Azerbaijan**				
Nutritional deficiencies	298.21(264.37to332.06)	-0.25(-0.49to0.00)	509.36(465.12to553.59)	-1.33(-1.95to-0.70)
Protein-energy malnutrition	282.44(249.50to315.37)	0.02(-0.26to0.29)	15.02(11.65to19.37)	-3.21(-3.41to-3.00)
Iodine deficiency	15.78(12.31to20.21)	-3.48(-3.79to-3.18)	5.10(3.29to7.88)	-11.89(-14.90to-8.77)
Vitamin A deficiency	2178.12(2086.65to2269.59)	-4.46(-6.12to-2.77)	0.00(0.00to0.00)	-
Dietary iron deficiency	-	-	480.73(437.76to523.71)	-0.97(-1.59to-0.34)
Other nutritional deficiency	-	-	8.51(6.07to11.92)	-2.81(-3.78to-1.83)
**Bahamas**				
Nutritional deficiencies	1613.09(1534.37to1691.81)	1.77(1.44to2.10)	507.40(463.25to551.55)	-0.30(-0.41to-0.20)
Protein-energy malnutrition	1602.82(1524.35to1681.29)	1.81(1.47to2.14)	58.72(43.70to73.74)	0.10(-0.25to0.46)
Iodine deficiency	10.27(2.12to49.70)	-2.25(-2.32to-2.18)	4.58(0.43to48.56)	-2.37(-2.66to-2.07)
Vitamin A deficiency	1678.90(1598.59to1759.21)	-2.46(-2.61to-2.30)	2.99(0.16to55.56)	0.37(0.24to0.50)
Dietary iron deficiency	-	-	415.62(375.66to455.58)	-0.57(-0.66to-0.48)
Other nutritional deficiency	-	-	25.50(15.60to35.40)	4.19(2.33to6.09)
**Bahrain**				
Nutritional deficiencies	1064.56(1000.61to1128.51)	1.03(0.84to1.21)	305.12(270.88to339.36)	-2.70(-3.45to-1.94)
Protein-energy malnutrition	1040.96(977.73to1104.20)	1.08(0.89to1.27)	5.31(1.53to18.42)	-4.52(-5.18to-3.86)
Iodine deficiency	23.59(14.07to33.11)	-1.13(-1.23to-1.04)	6.21(1.97to19.62)	-1.40(-1.45to-1.35)
Vitamin A deficiency	1441.95(1367.53to1516.38)	-4.13(-4.41to-3.85)	0.00(0.00to0.00)	-
Dietary iron deficiency	-	-	291.10(257.66to324.54)	-2.71(-3.50to-1.92)

Other nutritional deficiency	-	-	2.49(0.41to15.31)	2.76(1.36to4.18)
**Bangladesh**				
Nutritional deficiencies	892.20(833.65to950.74)	-1.24(-2.76to0.30)	627.52(578.42to676.61)	-1.87(-2.34to-1.38)
Protein-energy malnutrition	670.06(619.33to720.80)	-2.72(-4.20to-1.21)	48.05(34.46to61.64)	-8.27(-8.87to-7.67)
Iodine deficiency	222.13(192.92to251.34)	5.69(3.23to8.20)	65.48(49.62to81.34)	1.64(0.48to2.80)
Vitamin A deficiency	4661.44(4527.63to4795.26)	-2.69(-3.45to-1.92)	2.82(2.44to3.26)	-0.93(-1.06to-0.80)
Dietary iron deficiency	-	-	450.58(408.98to492.19)	-0.68(-1.23to-0.13)
Other nutritional deficiency	-	-	60.59(45.33to75.84)	-3.69(-4.56to-2.82)
**Barbados**				
Nutritional deficiencies	631.60(582.34to680.85)	-0.23(-0.42to-0.04)	230.99(201.20to260.78)	-0.54(-0.67to-0.40)
Protein-energy malnutrition	606.83(558.54to655.11)	-0.20(-0.41to0.01)	19.61(4.89to78.57)	-2.19(-2.55to-1.83)
Iodine deficiency	24.77(15.02to34.53)	-0.93(-1.06to-0.79)	11.96(2.02to70.73)	-0.80(-0.90to-0.70)
Vitamin A deficiency	2305.91(2211.80to2400.03)	-2.34(-2.71to-1.97)	1.84(0.02to171.27)	0.39(0.20to0.58)
Dietary iron deficiency	-	-	186.08(159.34to212.82)	-0.45(-0.60to-0.29)
Other nutritional deficiency	-	-	11.50(1.88to70.44)	1.29(-0.19to2.80)
**Belarus**				
Nutritional deficiencies	278.44(245.73to311.15)	-0.62(-1.10to-0.14)	149.04(125.11to172.97)	-1.87(-2.37to-1.36)
Protein-energy malnutrition	270.20(237.98to302.42)	-0.60(-1.13to-0.07)	21.54(12.44to30.63)	-1.05(-1.67to-0.42)
Iodine deficiency	8.24(5.58to12.17)	-1.11(-1.66to-0.55)	5.44(3.36to8.79)	-1.44(-2.04to-0.84)
Vitamin A deficiency	1862.89(1778.29to1947.49)	-4.32(-4.60to-4.04)	0.00(0.00to0.00)	-
Dietary iron deficiency	-	-	110.93(90.29to131.58)	-2.09(-2.70to-1.47)
Other nutritional deficiency	-	-	11.13(7.96to15.57)	0.05(-0.99to1.11)
**Belgium**				
Nutritional deficiencies	1210.60(1142.41to1278.80)	1.17(0.88to1.46)	109.95(89.40to130.50)	0.98(0.59to1.36)
Protein-energy malnutrition	1149.04(1082.60to1215.48)	1.24(0.93to1.55)	50.57(36.63to64.51)	1.49(1.23to1.76)
Iodine deficiency	61.56(46.18to76.94)	-0.17(-0.20to-0.14)	13.98(10.57to18.47)	-0.23(-0.28to-0.18)
Vitamin A deficiency	412.26(372.47to452.06)	-1.57(-1.64to-1.50)	0.00(0.00to0.00)	-
Dietary iron deficiency	-	-	22.77(13.41to32.12)	-2.24(-2.73to-1.75)
Other nutritional deficiency	-	-	22.64(13.31to31.96)	5.02(2.67to7.42)
**Belize**				
Nutritional deficiencies	504.73(460.70to548.76)	-1.16(-1.43to-0.89)	506.22(462.12to550.32)	-1.25(-1.33to-1.16)
Protein-energy malnutrition	497.76(454.03to541.49)	-1.15(-1.42to-0.87)	37.43(25.44to49.43)	-3.39(-4.02to-2.76)
Iodine deficiency	6.97(1.14to42.77)	-1.95(-1.99to-1.90)	3.19(0.22to46.62)	-2.27(-2.41to-2.13)
Vitamin A deficiency	4753.48(4618.35to4888.61)	-3.89(-4.18to-3.59)	2.80(0.16to49.00)	0.12(-0.02to0.26)
Dietary iron deficiency	-	-	452.35(410.67to494.04)	-1.08(-1.20to-0.96)
Other nutritional deficiency	-	-	10.45(2.38to45.98)	0.25(-0.83to1.33)

**Benin**				
Nutritional deficiencies	519.85(475.16to564.54)	-0.78(-0.95to-0.62)	414.43(374.53to454.33)	-2.30(-3.16to-1.44)
Protein-energy malnutrition	444.35(403.04to485.67)	-0.67(-0.85to-0.49)	54.15(39.73to68.58)	-4.31(-4.76to-3.86)
Iodine deficiency	75.50(58.47to92.53)	-1.40(-1.50to-1.30)	29.71(19.03to40.40)	-1.87(-2.01to-1.72)
Vitamin A deficiency	9860.58(9665.96to10055.21)	-2.23(-2.66to-1.79)	4.22(2.69to6.62)	-1.04(-1.46to-0.61)
Dietary iron deficiency	-	-	311.02(276.45to345.59)	-1.73(-2.83to-0.61)
Other nutritional deficiency	-	-	15.33(12.10to19.41)	-3.80(-4.21to-3.39)
**Bermuda**				
Nutritional deficiencies	689.64(638.17to741.11)	0.11(0.07to0.15)	218.38(189.41to247.34)	-2.49(-2.71to-2.27)
Protein-energy malnutrition	681.84(630.66to733.02)	0.17(0.13to0.21)	20.58(11.69to29.47)	-0.31(-0.36to-0.25)
Iodine deficiency	7.80(0.05to1176.95)	-3.28(-3.44to-3.12)	3.21(0.00to8030.98)	-3.69(-3.78to-3.61)
Vitamin A deficiency	1482.38(1406.92to1557.84)	-3.20(-3.41to-2.99)	0.00(0.00to0.00)	-
Dietary iron deficiency	-	-	184.98(158.32to211.64)	-2.88(-3.15to-2.61)
Other nutritional deficiency	-	-	9.61(0.10to881.76)	2.86(0.95to4.81)
**Bhutan**				
Nutritional deficiencies	1001.18(939.17to1063.20)	1.28(1.20to1.36)	856.71(799.34to914.08)	-1.27(-1.36to-1.19)
Protein-energy malnutrition	942.69(882.51to1002.87)	1.44(1.35to1.52)	35.28(23.63to46.92)	-0.69(-1.26to-0.12)
Iodine deficiency	58.50(43.51to73.49)	-0.50(-0.64to-0.37)	12.64(4.62to34.60)	-6.67(-8.80to-4.49)
Vitamin A deficiency	5381.94(5238.15to5525.73)	-4.15(-4.29to-4.02)	1.73(0.11to26.33)	-0.55(-0.60to-0.51)
Dietary iron deficiency	-	-	782.82(727.99to837.66)	-1.02(-1.11to-0.93)
Other nutritional deficiency	-	-	24.24(14.59to33.89)	-4.36(-5.32to-3.40)
**Bolivia**				
Nutritional deficiencies	434.03(393.20to474.86)	-1.11(-1.33to-0.90)	305.43(271.18to339.68)	-2.83(-3.01to-2.64)
Protein-energy malnutrition	427.98(387.43to468.53)	-1.12(-1.34to-0.90)	48.88(35.18to62.58)	-5.20(-5.72to-4.67)
Iodine deficiency	6.05(5.90to6.20)	-0.71(-0.84to-0.57)	2.71(1.54to4.76)	-1.77(-1.91to-1.62)
Vitamin A deficiency	8278.17(8099.84to8456.50)	-1.94(-2.62to-1.25)	3.34(2.01to5.55)	-0.41(-0.51to-0.31)
Dietary iron deficiency	-	-	235.85(205.75to265.95)	-2.33(-2.69to-1.97)
Other nutritional deficiency	-	-	14.66(12.93to16.62)	-3.05(-3.47to-2.63)
**Bosnia and Herzegovina**				
Nutritional deficiencies	593.53(545.78to641.28)	1.15(0.88to1.42)	238.12(207.87to268.36)	-1.45(-1.59to-1.31)
Protein-energy malnutrition	569.56(522.78to616.33)	1.35(1.10to1.60)	30.03(19.29to40.77)	1.21(0.99to1.42)
Iodine deficiency	23.97(14.38to33.57)	-2.17(-2.75to-1.58)	7.74(3.91to15.32)	-10.97(-14.38to-7.42)
Vitamin A deficiency	12698.73(12477.86to12919.60)	-3.66(-3.99to-3.33)	0.00(0.00to0.00)	-
Dietary iron deficiency	-	-	186.29(159.54to213.04)	-1.48(-1.69to-1.27)
Other nutritional deficiency	-	-	14.06(8.47to23.33)	4.31(2.39to6.27)

**Botswana**				
Nutritional deficiencies	399.64(360.45to438.82)	0.73(0.58to0.88)	307.44(273.08to341.81)	-1.26(-1.72to-0.80)
Protein-energy malnutrition	376.44(338.41to414.47)	0.80(0.64to0.96)	65.79(49.89to81.69)	-4.35(-5.73to-2.95)
Iodine deficiency	23.20(13.76to32.64)	-0.27(-0.31to-0.24)	4.73(1.89to11.82)	-0.64(-0.74to-0.54)
Vitamin A deficiency	6598.86(6439.64to6758.08)	-3.64(-4.18to-3.10)	4.70(1.88to11.78)	-1.18(-1.36to-1.00)
Dietary iron deficiency	-	-	223.81(194.49to253.13)	-0.09(-0.65to0.48)
Other nutritional deficiency	-	-	8.41(4.23to16.71)	-1.24(-2.21to-0.25)
**Brazil**				
Nutritional deficiencies	421.73(381.48to461.99)	0.11(0.08to0.14)	534.00(488.71to579.29)	-1.22(-1.36to-1.08)
Protein-energy malnutrition	414.37(374.47to454.27)	0.12(0.09to0.15)	27.98(17.61to38.35)	-2.99(-3.20to-2.78)
Iodine deficiency	7.36(6.80to7.97)	-0.35(-0.38to-0.32)	1.62(1.37to1.92)	-0.41(-0.51to-0.32)
Vitamin A deficiency	9027.19(8840.97to9213.41)	-2.95(-3.04to-2.87)	3.03(2.68to3.42)	-0.82(-1.08to-0.55)
Dietary iron deficiency	0.00(0.00to0.00)	-	493.14(449.62to536.67)	-1.13(-1.28to-0.98)
Other nutritional deficiency	0.00(0.00to0.00)	-	8.23(7.64to8.87)	0.60(-0.54to1.76)
**Brunei Darussalam**				
Nutritional deficiencies	484.86(441.70to528.02)	-0.28(-0.51to-0.05)	324.48(289.17to359.78)	-1.19(-1.47to-0.91)
Protein-energy malnutrition	468.09(425.69to510.50)	-0.28(-0.51to-0.04)	13.31(3.73to47.48)	-1.17(-1.32to-1.02)
Iodine deficiency	16.77(5.40to52.06)	-0.40(-0.46to-0.34)	3.79(0.35to41.08)	-0.38(-0.44to-0.33)
Vitamin A deficiency	1004.57(942.44to1066.69)	-2.93(-3.10to-2.77)	0.00(0.00to0.00)	-
Dietary iron deficiency	-	-	300.19(266.23to334.14)	-1.21(-1.52to-0.91)
Other nutritional deficiency	-	-	7.18(1.27to40.56)	-0.65(-2.08to0.80)
**Bulgaria**				
Nutritional deficiencies	499.99(456.17to543.82)	1.06(0.68to1.43)	206.00(177.87to234.13)	-0.96(-1.03to-0.89)
Protein-energy malnutrition	487.39(444.12to530.66)	1.16(0.78to1.54)	18.64(13.58to25.58)	1.14(0.63to1.65)
Iodine deficiency	12.60(8.57to18.52)	-1.81(-1.92to-1.69)	3.10(1.43to6.75)	-10.91(-14.04to-7.67)
Vitamin A deficiency	11584.77(11373.81to11795.73)	-2.31(-2.45to-2.17)	0.00(0.00to0.00)	-
Dietary iron deficiency	-	-	174.35(148.47to200.23)	-1.07(-1.27to-0.87)
Other nutritional deficiency	-	-	9.91(6.42to15.30)	4.99(3.31to6.70)
**Burkina Faso**				
Nutritional deficiencies	515.87(471.35to560.39)	-0.93(-1.42to-0.44)	492.67(449.16to536.17)	-0.67(-1.63to0.31)
Protein-energy malnutrition	441.35(400.18to482.53)	-0.59(-1.30to0.11)	76.57(59.42to93.72)	-2.95(-4.85to-1.01)
Iodine deficiency	74.51(57.60to91.43)	-2.55(-3.11to-1.98)	30.29(19.50to41.07)	-2.53(-2.59to-2.48)
Vitamin A deficiency	14822.97(14584.34to15061.60)	-2.16(-2.73to-1.59)	4.06(2.88to5.73)	-0.39(-0.48to-0.31)
Dietary iron deficiency	-	-	351.42(314.68to388.16)	1.49(0.82to2.17)
Other nutritional deficiency	-	-	30.33(19.54to41.13)	-3.33(-4.57to-2.08)

**Burundi**				
Nutritional deficiencies	593.90(546.14to641.67)	-0.18(-0.40to0.04)	401.40(362.14to440.67)	-5.28(-5.65to-4.90)
Protein-energy malnutrition	415.74(375.77to455.70)	0.50(0.28to0.72)	173.35(147.55to199.16)	-7.24(-7.97to-6.50)
Iodine deficiency	178.17(152.01to204.33)	-1.51(-1.76to-1.25)	68.04(51.87to84.21)	-1.27(-1.63to-0.91)
Vitamin A deficiency	18662.12(18394.37to18929.88)	-2.80(-3.21to-2.40)	2.84(1.61to5.03)	-0.98(-1.15to-0.81)
Dietary iron deficiency	-	-	131.64(109.15to154.12)	-0.78(-1.25to-0.32)
Other nutritional deficiency	-	-	25.53(15.63to35.43)	-8.33(-8.72to-7.93)
**Cabo Verde**				
Nutritional deficiencies	608.27(559.93to656.61)	-0.70(-0.77to-0.64)	420.23(380.05to460.41)	-0.58(-0.65to-0.51)
Protein-energy malnutrition	491.64(448.18to535.10)	-0.60(-0.74to-0.46)	26.31(16.26to36.36)	-2.71(-2.87to-2.56)
Iodine deficiency	116.63(95.46to137.79)	-1.04(-1.26to-0.82)	29.72(19.04to40.41)	-2.70(-2.99to-2.41)
Vitamin A deficiency	2909.71(2803.98to3015.43)	-4.51(-4.72to-4.30)	2.86(0.24to34.61)	-0.11(-0.38to0.16)
Dietary iron deficiency	-	-	350.66(313.96to387.36)	-0.25(-0.30to-0.20)
Other nutritional deficiency	-	-	10.67(2.93to38.80)	0.62(-0.73to1.98)
**Cambodia**				
Nutritional deficiencies	868.60(810.84to926.37)	-1.00(-1.49to-0.51)	561.62(515.17to608.07)	-3.25(-3.45to-3.05)
Protein-energy malnutrition	794.31(739.07to849.55)	1.15(0.80to1.49)	41.41(28.80to54.03)	-9.04(-9.32to-8.75)
Iodine deficiency	74.29(57.40to91.19)	-9.79(-10.30to-9.27)	27.31(17.07to37.55)	-17.12(-19.59to-14.58)
Vitamin A deficiency	5213.70(5072.18to5355.23)	-5.50(-6.47to-4.51)	1.00(0.66to1.54)	-2.31(-2.49to-2.14)
Dietary iron deficiency	-	-	480.95(437.97to523.94)	-1.12(-1.36to-0.88)
Other nutritional deficiency	-	-	10.94(8.66to13.82)	1.38(0.14to2.64)
**Cameroon**				
Nutritional deficiencies	444.57(403.25to485.90)	0.14(-0.02to0.31)	367.88(330.28to405.47)	-0.80(-1.78to0.18)
Protein-energy malnutrition	385.74(347.25to424.24)	0.26(0.07to0.45)	49.59(35.79to63.40)	-4.31(-4.57to-4.05)
Iodine deficiency	58.83(43.80to73.87)	-0.58(-0.62to-0.54)	22.91(13.53to32.29)	-0.83(-0.93to-0.74)
Vitamin A deficiency	9577.54(9385.73to9769.36)	-2.88(-3.27to-2.48)	3.07(2.19to4.32)	-0.66(-1.28to-0.04)
Dietary iron deficiency	-	-	277.35(244.71to309.99)	0.88(-0.78to2.57)
Other nutritional deficiency	-	-	14.95(12.81to17.43)	-3.45(-3.89to-3.00)
**Canada**				
Nutritional deficiencies	629.00(579.84to678.15)	0.19(0.12to0.27)	93.87(74.88to112.86)	-1.33(-1.57to-1.10)
Protein-energy malnutrition	613.49(564.94to662.03)	0.20(0.12to0.28)	15.71(13.58to18.18)	-0.04(-0.12to0.03)
Iodine deficiency	15.51(13.39to17.96)	-0.11(-0.18to-0.05)	3.46(2.53to4.71)	-0.29(-0.33to-0.25)
Vitamin A deficiency	932.32(872.48to992.17)	-1.56(-1.79to-1.33)	0.00(0.00to0.00)	-
Dietary iron deficiency	-	-	67.66(51.54to83.78)	-2.13(-2.33to-1.92)
Other nutritional deficiency	-	-	7.05(5.67to8.76)	3.51(1.44to5.62)

**Central African Republic**				
Nutritional deficiencies	591.34(543.68to639.00)	-1.69(-1.78to-1.60)	606.92(558.63to655.21)	-1.08(-1.34to-0.82)
Protein-energy malnutrition	305.59(271.33to339.85)	-0.46(-0.78to-0.15)	207.76(179.51to236.02)	-0.30(-1.28to0.68)
Iodine deficiency	285.75(252.62to318.88)	-2.98(-3.17to-2.79)	150.71(126.65to174.78)	-2.60(-2.72to-2.48)
Vitamin A deficiency	13895.54(13664.49to14126.58)	-0.99(-1.12to-0.86)	7.73(4.67to12.78)	1.08(0.23to1.94)
Dietary iron deficiency	-	-	213.02(184.41to241.62)	-0.74(-1.42to-0.05)
Other nutritional deficiency	-	-	27.70(17.38to38.01)	-1.79(-2.24to-1.33)
**Chad**				
Nutritional deficiencies	550.10(504.13to596.07)	-1.13(-1.28to-0.98)	501.83(457.92to545.74)	-2.23(-2.38to-2.08)
Protein-energy malnutrition	464.46(422.22to506.70)	-0.16(-0.44to0.12)	102.27(82.45to122.09)	-3.58(-4.13to-3.02)
Iodine deficiency	85.64(67.50to103.77)	-4.30(-4.77to-3.82)	33.46(22.13to44.80)	-3.17(-3.27to-3.08)
Vitamin A deficiency	14702.05(14464.40to14939.71)	-1.34(-1.90to-0.77)	6.84(4.94to9.46)	-0.24(-0.51to0.03)
Dietary iron deficiency	-	-	335.73(299.82to371.65)	-1.22(-1.36to-1.09)
Other nutritional deficiency	-	-	23.52(14.02to33.03)	-3.18(-3.69to-2.67)
**Chile**				
Nutritional deficiencies	522.50(477.70to567.31)	-0.17(-0.49to0.15)	78.89(61.48to96.30)	-2.09(-2.60to-1.58)
Protein-energy malnutrition	514.74(470.27to559.21)	-0.16(-0.49to0.17)	22.75(13.40to32.09)	-0.43(-0.78to-0.09)
Iodine deficiency	7.76(5.90to10.21)	-0.75(-0.81to-0.69)	2.08(1.22to3.53)	-1.93(-1.99to-1.86)
Vitamin A deficiency	2957.63(2851.04to3064.22)	-0.58(-1.10to-0.05)	0.00(0.00to0.00)	-
Dietary iron deficiency	-	-	45.55(32.32to58.77)	-3.57(-4.06to-3.07)
Other nutritional deficiency	-	-	8.52(6.56to11.06)	3.14(0.83to5.51)
**China**				
Nutritional deficiencies	1318.79(1247.61to1389.97)	2.64(2.49to2.79)	169.29(143.79to194.79)	-4.25(-4.46to-4.04)
Protein-energy malnutrition	1174.75(1107.58to1241.93)	2.66(2.16to3.16)	23.57(14.05to33.08)	-0.13(-1.50to1.25)
Iodine deficiency	144.04(120.52to167.56)	2.47(0.38to4.61)	32.07(20.97to43.18)	2.11(0.31to3.93)
Vitamin A deficiency	1930.84(1844.72to2016.97)	-5.42(-5.98to-4.85)	1.47(1.37to1.58)	-0.68(-1.35to-0.01)
Dietary iron deficiency	0.00(0.00to0.00)	-	101.10(81.39to120.80)	-6.28(-6.53to-6.03)
Other nutritional deficiency	0.00(0.00to0.00)	-	11.07(10.79to11.37)	5.25(3.62to6.91)
**Colombia**				
Nutritional deficiencies	553.05(506.95to599.14)	0.13(0.10to0.17)	125.13(103.20to147.05)	-4.62(-5.31to-3.94)
Protein-energy malnutrition	530.97(485.81to576.14)	0.18(0.14to0.22)	29.16(18.58to39.74)	-2.76(-3.20to-2.32)
Iodine deficiency	22.07(12.87to31.28)	-0.73(-0.88to-0.59)	14.24(12.76to15.89)	-7.30(-9.45to-5.10)
Vitamin A deficiency	2875.59(2770.48to2980.69)	-3.78(-4.05to-3.52)	3.11(2.48to3.88)	-0.30(-0.36to-0.23)
Dietary iron deficiency	-	-	73.89(57.04to90.74)	-5.16(-6.37to-3.92)
Other nutritional deficiency	-	-	4.74(4.28to5.25)	-0.47(-1.25to0.32)
**Comoros**				

Nutritional deficiencies	522.25(477.46to567.04)	-0.10(-0.26to0.06)	630.43(581.22to679.65)	-4.15(-4.68to-3.61)
Protein-energy malnutrition	490.13(446.74to533.52)	-0.13(-0.30to0.05)	117.20(95.98to138.42)	-6.98(-8.93to-4.98)
Iodine deficiency	32.12(21.01to43.23)	0.29(0.17to0.42)	13.80(5.01to38.03)	0.34(0.17to0.50)
Vitamin A deficiency	13636.08(13407.20to13864.95)	-3.07(-3.24to-2.89)	3.64(0.51to26.19)	-1.18(-1.27to-1.08)
Dietary iron deficiency	-	-	476.34(433.56to519.12)	-2.95(-3.79to-2.09)
Other nutritional deficiency	-	-	19.46(8.29to45.69)	-6.93(-8.55to-5.28)
**Congo**				
Nutritional deficiencies	755.41(701.54to809.28)	-1.09(-1.16to-1.02)	712.68(660.36to765.01)	-1.32(-1.51to-1.13)
Protein-energy malnutrition	332.24(296.51to367.96)	0.67(0.58to0.75)	96.73(77.45to116.01)	-1.85(-2.61to-1.09)
Iodine deficiency	423.17(382.85to463.49)	-2.21(-2.34to-2.08)	223.51(194.21to252.81)	-1.92(-2.10to-1.75)
Vitamin A deficiency	11250.79(11042.89to11458.69)	-1.67(-2.99to-0.34)	7.68(4.65to12.68)	-1.81(-1.97to-1.66)
Dietary iron deficiency	-	-	371.12(333.36to408.88)	-0.31(-0.78to0.16)
Other nutritional deficiency	-	-	13.64(9.37to19.88)	-4.67(-5.65to-3.69)
**Cook Islands**				
Nutritional deficiencies	1157.68(1090.99to1224.37)	0.32(0.22to0.43)	347.99(311.43to384.55)	-0.55(-1.04to-0.07)
Protein-energy malnutrition	1153.46(1086.89to1220.03)	0.33(0.22to0.44)	29.54(18.89to40.20)	0.26(0.20to0.32)
Iodine deficiency	4.22(0.00to746812.89)	-1.53(-1.67to-1.40)	0.75(0.00to1.99E+12)	-1.68(-1.82to-1.54)
Vitamin A deficiency	2530.04(2431.45to2628.62)	-3.21(-3.69to-2.73)	2.40(0.00to21733903.10)	-0.88(-0.97to-0.79)
Dietary iron deficiency	-	-	302.37(268.29to336.46)	-0.76(-1.26to-0.26)
Other nutritional deficiency	-	-	12.92(0.01to12914.33)	4.22(1.91to6.59)
**Costa Rica**				
Nutritional deficiencies	691.27(639.74to742.80)	0.67(0.60to0.74)	163.29(138.25to188.34)	-2.14(-2.43to-1.86)
Protein-energy malnutrition	670.17(619.43to720.91)	0.72(0.65to0.79)	17.57(12.47to24.75)	0.57(0.19to0.95)
Iodine deficiency	21.10(12.09to30.10)	-0.65(-0.74to-0.56)	7.76(4.63to13.00)	-7.68(-10.03to-5.26)
Vitamin A deficiency	2806.23(2702.40to2910.06)	-2.57(-2.90to-2.23)	2.60(1.07to6.34)	-0.14(-0.32to0.04)
Dietary iron deficiency	-	-	127.45(105.32to149.57)	-2.28(-2.50to-2.06)
Other nutritional deficiency	-	-	7.92(4.75to13.20)	3.02(1.10to4.98)
**Croatia**				
Nutritional deficiencies	369.54(331.86to407.22)	-0.09(-0.42to0.24)	125.89(103.90to147.88)	-0.52(-0.65to-0.40)
Protein-energy malnutrition	356.51(319.50to393.52)	-0.03(-0.37to0.30)	12.40(7.60to20.23)	0.10(-0.23to0.43)
Iodine deficiency	13.03(8.09to21.01)	-1.42(-1.69to-1.15)	3.22(1.23to8.41)	-1.61(-2.01to-1.20)
Vitamin A deficiency	10334.66(10135.41to10533.91)	-2.34(-2.46to-2.21)	0.00(0.00to0.00)	-
Dietary iron deficiency	-	-	104.29(84.28to124.31)	-0.72(-0.83to-0.62)
Other nutritional deficiency	-	-	5.98(2.96to12.10)	2.83(1.07to4.62)
**Cuba**				
Nutritional deficiencies	611.08(562.63to659.53)	0.90(0.87to0.92)	328.40(292.88to363.92)	-1.43(-1.74to-1.11)

Protein-energy malnutrition	591.48(543.82to639.15)	1.14(1.10to1.18)	30.87(19.98to41.76)	0.56(0.30to0.82)
Iodine deficiency	19.60(15.56to24.69)	-3.05(-3.54to-2.57)	10.21(7.41to14.05)	-3.54(-3.87to-3.21)
Vitamin A deficiency	2344.85(2249.94to2439.76)	-2.31(-2.85to-1.77)	0.00(0.00to0.00)	-
Dietary iron deficiency	-	-	272.88(240.50to305.25)	-1.67(-2.02to-1.32)
Other nutritional deficiency	-	-	14.44(11.04to18.90)	3.47(1.55to5.44)
**Cyprus**				
Nutritional deficiencies	1138.84(1072.70to1204.99)	0.81(0.63to0.98)	113.00(92.17to133.84)	-1.26(-1.48to-1.05)
Protein-energy malnutrition	1072.71(1008.51to1136.90)	0.86(0.68to1.05)	45.63(32.39to58.87)	1.14(0.95to1.33)
Iodine deficiency	66.14(50.20to82.08)	-0.06(-0.10to-0.01)	15.05(7.33to30.92)	-0.17(-0.21to-0.13)
Vitamin A deficiency	479.53(436.61to522.45)	-3.31(-3.55to-3.06)	0.00(0.00to0.00)	-
Dietary iron deficiency	-	-	31.63(20.61to42.66)	-6.39(-6.64to-6.15)
Other nutritional deficiency	-	-	20.69(11.77to29.60)	4.74(2.65to6.86)
**Czechia**				
Nutritional deficiencies	605.55(557.31to653.78)	3.09(1.63to4.56)	115.00(93.98to136.02)	-0.34(-0.53to-0.16)
Protein-energy malnutrition	595.85(548.01to643.69)	3.22(1.74to4.73)	10.71(7.69to14.93)	4.97(2.93to7.05)
Iodine deficiency	9.70(6.84to13.74)	-1.14(-1.44to-0.83)	2.25(1.09to4.64)	-1.26(-1.67to-0.85)
Vitamin A deficiency	8070.11(7894.04to8246.18)	-1.74(-1.91to-1.58)	0.00(0.00to0.00)	-
Dietary iron deficiency	-	-	98.15(78.73to117.57)	-0.67(-0.76to-0.57)
Other nutritional deficiency	-	-	3.89(2.24to6.74)	5.30(3.82to6.81)
**Côte d'Ivoire**				
Nutritional deficiencies	461.33(419.23to503.43)	-0.19(-0.27to-0.11)	390.07(351.36to428.78)	-1.69(-2.05to-1.34)
Protein-energy malnutrition	362.79(325.46to400.13)	0.02(-0.15to0.18)	38.44(26.29to50.59)	-3.02(-3.14to-2.90)
Iodine deficiency	98.54(79.08to117.99)	-0.78(-0.96to-0.60)	34.37(22.88to45.86)	-1.25(-1.31to-1.19)
Vitamin A deficiency	9490.71(9299.76to9681.65)	-2.50(-2.57to-2.43)	4.33(3.18to5.88)	-1.01(-1.13to-0.89)
Dietary iron deficiency	-	-	301.63(267.59to335.67)	-1.39(-1.91to-0.87)
Other nutritional deficiency	-	-	11.31(9.35to13.68)	-2.49(-2.81to-2.17)
**Democratic People s Republic of Korea**				
Nutritional deficiencies	867.89(810.14to925.63)	-0.44(-1.86to1.00)	474.42(431.73to517.11)	-22.57(-33.14to-10.33)
Protein-energy malnutrition	861.69(804.16to919.23)	-0.43(-1.87to1.03)	16.44(15.55to17.38)	-48.21(-65.02to-23.31)
Iodine deficiency	6.19(4.79to8.00)	-1.66(-1.98to-1.34)	2.69(2.15to3.36)	-14.06(-17.84to-10.10)
Vitamin A deficiency	4972.70(4834.49to5110.91)	-3.36(-3.60to-3.12)	0.40(0.19to0.84)	-0.48(-0.73to-0.24)
Dietary iron deficiency	-	-	449.33(407.78to490.88)	-0.80(-1.53to-0.06)
Other nutritional deficiency	-	-	5.56(5.23to5.92)	1.90(0.78to3.03)

**Democratic Republic of the Congo**				
Nutritional deficiencies	1067.30(1003.27to1131.33)	-1.01(-1.24to-0.78)	686.95(635.58to738.32)	-1.62(-1.71to-1.53)
Protein-energy malnutrition	578.76(531.61to625.91)	0.23(-0.21to0.68)	87.27(68.96to105.58)	-2.40(-2.83to-1.97)
Iodine deficiency	488.54(445.22to531.86)	-2.28(-2.41to-2.15)	319.74(284.69to354.79)	-0.85(-1.08to-0.62)
Vitamin A deficiency	15252.34(15010.28to15494.40)	0.57(-0.17to1.31)	5.74(4.95to6.65)	0.03(-0.17to0.23)
Dietary iron deficiency	-	-	255.37(224.04to286.69)	-2.17(-2.81to-1.52)
Other nutritional deficiency	-	-	18.83(17.36to20.43)	-3.44(-3.85to-3.03)
**Denmark**				
Nutritional deficiencies	946.87(886.55to1007.18)	1.36(0.40to2.32)	104.52(84.48to124.55)	0.05(-0.59to0.69)
Protein-energy malnutrition	898.87(840.11to957.64)	1.43(0.42to2.44)	39.40(27.09to51.70)	1.39(0.34to2.45)
Iodine deficiency	47.99(34.42to61.57)	0.09(-0.01to0.19)	10.45(6.64to16.42)	0.02(-0.02to0.07)
Vitamin A deficiency	341.18(304.97to377.38)	-1.25(-1.30to-1.19)	0.00(0.00to0.00)	-
Dietary iron deficiency	-	-	36.38(24.56to48.20)	-2.53(-2.58to-2.48)
Other nutritional deficiency	-	-	18.29(13.00to25.75)	4.23(1.66to6.86)
**Djibouti**				
Nutritional deficiencies	1354.13(1282.00to1426.25)	-0.73(-1.02to-0.45)	751.84(698.09to805.58)	-2.20(-2.58to-1.83)
Protein-energy malnutrition	549.74(503.78to595.69)	-2.13(-2.66to-1.61)	147.97(124.13to171.82)	-4.73(-6.03to-3.42)
Iodine deficiency	804.39(748.80to859.98)	0.12(-0.64to0.89)	238.90(208.60to269.19)	0.17(-0.66to1.00)
Vitamin A deficiency	11561.49(11350.74to11772.23)	-5.21(-6.01to-4.40)	3.24(0.64to16.55)	-1.26(-1.44to-1.09)
Dietary iron deficiency	-	-	340.89(304.71to377.08)	-1.85(-2.05to-1.66)
Other nutritional deficiency	-	-	20.82(11.88to29.77)	-4.04(-5.12to-2.94)
**Dominica**				
Nutritional deficiencies	657.29(607.04to707.54)	-0.94(-1.11to-0.77)	502.50(458.56to546.44)	-0.41(-0.61to-0.21)
Protein-energy malnutrition	639.85(590.27to689.43)	-0.88(-1.04to-0.72)	34.22(22.75to45.69)	-3.98(-4.20to-3.76)
Iodine deficiency	17.45(6.92to44.00)	-2.66(-3.08to-2.23)	8.54(0.18to403.45)	-2.93(-3.31to-2.55)
Vitamin A deficiency	2452.36(2355.30to2549.43)	-3.49(-4.15to-2.82)	2.88(0.00to2073.20)	0.28(0.16to0.41)
Dietary iron deficiency	-	-	442.93(401.68to484.18)	0.04(-0.23to0.32)
Other nutritional deficiency	-	-	13.93(3.50to55.48)	0.57(-0.87to2.03)
**Dominican Republic**				
Nutritional deficiencies	320.42(285.33to355.50)	-1.16(-1.62to-0.70)	391.08(352.32to429.84)	-0.62(-0.96to-0.28)
Protein-energy malnutrition	299.67(265.74to333.60)	-1.07(-1.60to-0.54)	54.02(39.62to68.43)	-2.08(-2.76to-1.39)
Iodine deficiency	20.75(11.82to29.67)	-2.23(-2.59to-1.87)	9.99(7.37to13.54)	-2.63(-2.80to-2.46)
Vitamin A deficiency	4881.44(4744.50to5018.38)	-6.15(-6.50to-5.80)	2.94(1.67to5.15)	0.10(0.00to0.20)
Dietary iron deficiency	-	-	317.63(282.70to352.56)	-0.38(-0.69to-0.06)
Other nutritional deficiency	-	-	6.50(4.46to9.48)	3.63(1.99to5.30)

**Ecuador**				
Nutritional deficiencies	549.12(503.19to595.04)	-2.59(-2.86to-2.31)	161.70(136.78to186.63)	-3.22(-3.57to-2.87)
Protein-energy malnutrition	543.61(497.91to589.31)	-2.61(-2.89to-2.33)	25.96(15.97to35.94)	-5.58(-7.03to-4.12)
Iodine deficiency	5.51(3.99to7.60)	-0.36(-0.51to-0.21)	1.11(0.54to2.28)	-0.41(-0.60to-0.22)
Vitamin A deficiency	3905.82(3783.32to4028.31)	-2.89(-3.40to-2.38)	3.14(2.05to4.81)	-0.11(-0.25to0.02)
Dietary iron deficiency	-	-	120.19(98.70to141.68)	-3.02(-3.12to-2.92)
Other nutritional deficiency	-	-	11.30(9.03to14.15)	1.12(-0.87to3.16)
**Egypt**				
Nutritional deficiencies	682.47(631.26to733.67)	-0.27(-0.29to-0.25)	246.04(215.30to276.79)	-1.94(-2.06to-1.81)
Protein-energy malnutrition	641.47(591.82to691.11)	0.02(-0.03to0.07)	20.02(11.25to28.80)	-1.14(-1.30to-0.97)
Iodine deficiency	41.00(28.45to53.55)	-3.51(-3.85to-3.16)	29.96(19.23to40.68)	-6.31(-7.02to-5.59)
Vitamin A deficiency	1867.32(1782.62to1952.02)	-1.80(-2.36to-1.24)	1.60(1.24to2.07)	-0.53(-0.77to-0.29)
Dietary iron deficiency	-	-	185.70(158.99to212.41)	-1.27(-1.30to-1.24)
Other nutritional deficiency	-	-	8.76(7.85to9.77)	2.47(0.75to4.23)
**El Salvador**				
Nutritional deficiencies	715.88(663.44to768.33)	1.52(1.17to1.87)	137.21(114.25to160.17)	-0.70(-0.98to-0.43)
Protein-energy malnutrition	692.30(640.73to743.87)	1.63(1.27to1.99)	45.37(32.17to58.57)	-0.45(-1.09to0.20)
Iodine deficiency	23.58(14.07to33.10)	-1.09(-1.21to-0.97)	16.20(11.95to21.96)	-8.23(-10.54to-5.86)
Vitamin A deficiency	5108.18(4968.09to5248.26)	-4.60(-5.19to-4.02)	3.21(1.62to6.36)	-0.44(-0.58to-0.30)
Dietary iron deficiency	-	-	66.13(50.19to82.06)	0.18(-0.07to0.43)
Other nutritional deficiency	-	-	6.31(3.87to10.27)	5.07(2.86to7.32)
**Equatorial Guinea**				
Nutritional deficiencies	481.83(438.81to524.85)	-6.56(-7.78to-5.33)	369.83(332.14to407.52)	-6.73(-8.37to-5.06)
Protein-energy malnutrition	225.69(196.25to255.14)	-2.36(-3.60to-1.10)	39.81(27.45to52.18)	-7.91(-9.04to-6.76)
Iodine deficiency	256.14(224.77to287.50)	-8.98(-10.10to-7.83)	113.92(93.00to134.84)	-11.34(-12.94to-9.72)
Vitamin A deficiency	2645.30(2544.49to2746.10)	-11.42(-11.68to-11.15)	12.25(5.55to27.03)	-5.55(-5.68to-5.41)
Dietary iron deficiency	-	-	195.88(168.45to223.31)	-2.84(-4.58to-1.07)
Other nutritional deficiency	-	-	7.96(2.98to21.25)	-7.74(-9.06to-6.39)
**Eritrea**				
Nutritional deficiencies	430.56(389.89to471.23)	-0.46(-0.60to-0.33)	612.60(564.09to661.11)	-3.00(-3.46to-2.54)
Protein-energy malnutrition	390.38(351.65to429.10)	-0.52(-0.68to-0.37)	205.35(177.26to233.44)	-3.52(-4.20to-2.84)
Iodine deficiency	40.19(27.76to52.61)	0.13(0.04to0.21)	17.59(13.09to23.62)	-1.26(-1.76to-0.76)
Vitamin A deficiency	15997.53(15749.63to16245.43)	-2.42(-2.69to-2.15)	4.10(2.22to7.55)	-1.08(-1.37to-0.80)
Dietary iron deficiency	-	-	355.92(318.94to392.89)	-2.37(-2.65to-2.08)

Other nutritional deficiency	-	-	29.65(18.98to40.32)	-4.47(-5.43to-3.50)
**Estonia**				
Nutritional deficiencies	281.35(248.47to314.23)	1.45(1.29to1.62)	119.37(97.96to140.79)	-3.11(-3.19to-3.03)
Protein-energy malnutrition	275.20(242.68to307.71)	1.53(1.35to1.70)	13.05(5.53to30.80)	-1.33(-1.67to-0.98)
Iodine deficiency	6.15(1.76to21.49)	-1.02(-1.14to-0.90)	3.61(0.71to18.48)	-1.20(-1.25to-1.15)
Vitamin A deficiency	1102.54(1037.46to1167.62)	-3.74(-4.01to-3.48)	0.00(0.00to0.00)	-
Dietary iron deficiency	-	-	96.05(76.84to115.26)	-3.62(-3.66to-3.59)
Other nutritional deficiency	-	-	6.67(2.00to22.17)	4.09(2.38to5.83)
**Eswatini**				
Nutritional deficiencies	356.86(319.83to393.88)	-0.98(-1.32to-0.63)	241.30(210.85to271.74)	-0.62(-1.38to0.14)
Protein-energy malnutrition	295.83(262.12to329.54)	-1.00(-1.44to-0.56)	55.98(41.31to70.64)	-0.83(-2.62to0.99)
Iodine deficiency	61.03(45.72to76.34)	-0.86(-0.99to-0.73)	15.17(7.22to31.85)	-0.66(-0.78to-0.53)
Vitamin A deficiency	6031.37(5879.16to6183.59)	-2.77(-3.04to-2.50)	5.02(1.38to18.24)	-0.79(-0.89to-0.69)
Dietary iron deficiency	-	-	158.51(133.83to183.18)	-0.49(-0.71to-0.28)
Other nutritional deficiency	-	-	6.63(2.16to20.36)	-0.67(-2.31to1.00)
**Ethiopia**				
Nutritional deficiencies	1174.25(1107.09to1241.41)	-0.73(-1.66to0.20)	455.05(413.24to496.86)	-5.78(-6.24to-5.32)
Protein-energy malnutrition	524.87(479.97to569.78)	-1.40(-1.54to-1.27)	92.86(73.98to111.75)	-12.74(-13.19to-12.29)
Iodine deficiency	649.38(599.43to699.32)	-0.29(-1.95to1.39)	192.03(164.87to219.19)	0.39(-0.31to1.10)
Vitamin A deficiency	19004.43(18734.23to19274.63)	-3.18(-3.80to-2.55)	7.09(6.30to7.97)	1.21(0.64to1.78)
Dietary iron deficiency	0.00(0.00to0.00)	-	143.97(120.45to167.49)	-0.29(-0.73to0.15)
Other nutritional deficiency	0.00(0.00to0.00)	-	19.09(17.77to20.51)	-12.65(-12.91to-12.40)
**Fiji**				
Nutritional deficiencies	778.00(723.34to832.67)	-0.79(-0.88to-0.70)	508.47(464.27to552.66)	-0.49(-0.99to0.02)
Protein-energy malnutrition	773.21(718.71to827.71)	-0.78(-0.87to-0.70)	22.30(13.05to31.56)	-2.87(-3.23to-2.50)
Iodine deficiency	4.79(1.01to22.80)	-1.77(-2.16to-1.39)	0.86(0.02to34.23)	-1.88(-2.17to-1.60)
Vitamin A deficiency	2793.05(2689.47to2896.64)	-3.00(-3.83to-2.17)	2.37(0.26to21.78)	-0.61(-0.69to-0.54)
Dietary iron deficiency	-	-	476.14(433.37to518.91)	-0.40(-0.92to0.12)
Other nutritional deficiency	-	-	6.80(1.84to25.19)	2.90(0.76to5.08)
**Finland**				
Nutritional deficiencies	919.16(859.74to978.59)	0.18(-0.03to0.38)	122.87(101.15to144.60)	-1.20(-1.55to-0.84)
Protein-energy malnutrition	855.62(798.28to912.95)	0.21(-0.01to0.43)	38.02(25.93to50.10)	0.46(0.24to0.68)
Iodine deficiency	63.55(47.92to79.17)	-0.26(-0.33to-0.20)	14.40(9.59to21.61)	-0.26(-0.27to-0.24)
Vitamin A deficiency	414.50(374.59to454.40)	-1.79(-1.85to-1.73)	0.00(0.00to0.00)	-
Dietary iron deficiency	-	-	52.61(38.39to66.82)	-3.50(-4.12to-2.87)

Other nutritional deficiency	-	-	17.85(12.40to25.71)	3.60(1.70to5.53)
**France**				
Nutritional deficiencies	1444.07(1369.59to1518.55)	0.32(0.23to0.41)	118.01(96.71to139.30)	0.33(-0.02to0.67)
Protein-energy malnutrition	1380.19(1307.37to1453.00)	0.33(0.24to0.43)	60.65(45.39to75.92)	0.44(0.36to0.51)
Iodine deficiency	63.88(48.22to79.55)	0.05(0.02to0.08)	14.48(12.91to16.23)	0.07(0.06to0.08)
Vitamin A deficiency	127.80(105.64to149.96)	-1.17(-1.21to-1.12)	0.00(0.00to0.00)	-
Dietary iron deficiency	-	-	16.05(14.40to17.89)	-4.54(-5.46to-3.60)
Other nutritional deficiency	-	-	26.82(16.67to36.98)	4.16(1.97to6.38)
**Gabon**				
Nutritional deficiencies	505.68(461.61to549.76)	-3.72(-3.94to-3.50)	721.69(669.03to774.34)	0.20(-0.68to1.08)
Protein-energy malnutrition	361.89(324.61to399.18)	0.05(-0.19to0.28)	46.83(33.41to60.24)	-4.10(-4.99to-3.19)
Iodine deficiency	143.79(120.29to167.30)	-8.21(-8.81to-7.60)	56.11(41.43to70.79)	-9.68(-10.36to-9.00)
Vitamin A deficiency	2781.93(2678.55to2885.31)	-3.15(-3.50to-2.80)	6.59(2.67to16.30)	-1.02(-1.16to-0.89)
Dietary iron deficiency	-	-	602.51(554.40to650.62)	3.18(1.84to4.53)
Other nutritional deficiency	-	-	9.65(4.57to20.39)	-4.43(-5.03to-3.83)
**Gambia**				
Nutritional deficiencies	692.88(641.29to744.47)	-1.18(-1.55to-0.81)	797.06(741.72to852.39)	0.28(0.03to0.52)
Protein-energy malnutrition	446.16(404.76to487.56)	-0.63(-0.76to-0.51)	46.01(32.71to59.30)	-1.50(-2.49to-0.51)
Iodine deficiency	246.72(215.93to277.51)	-1.95(-2.66to-1.23)	80.10(62.56to97.65)	-1.84(-2.52to-1.16)
Vitamin A deficiency	10699.24(10496.51to10901.98)	-2.98(-3.14to-2.82)	3.81(1.29to11.25)	-0.53(-1.08to0.01)
Dietary iron deficiency	-	-	653.58(603.47to703.69)	1.01(0.93to1.10)
Other nutritional deficiency	-	-	13.56(7.64to24.08)	-1.86(-2.89to-0.83)
**Georgia**				
Nutritional deficiencies	297.27(263.48to331.07)	-0.54(-1.21to0.14)	383.91(345.51to422.32)	-0.42(-0.48to-0.35)
Protein-energy malnutrition	276.32(243.74to308.91)	-0.53(-1.25to0.20)	14.66(9.12to23.58)	-0.51(-1.00to-0.02)
Iodine deficiency	20.95(11.98to29.92)	-0.68(-0.81to-0.56)	7.84(4.09to15.01)	-1.12(-1.20to-1.04)
Vitamin A deficiency	2039.99(1951.46to2128.51)	-1.43(-2.11to-0.74)	0.00(0.00to0.00)	-
Dietary iron deficiency	-	-	353.70(316.84to390.56)	-0.44(-0.53to-0.35)
Other nutritional deficiency	-	-	7.72(4.01to14.86)	2.38(0.35to4.45)
**Germany**				
Nutritional deficiencies	908.27(849.20to967.34)	0.12(-1.05to1.29)	126.34(104.31to148.37)	0.67(0.31to1.02)
Protein-energy malnutrition	845.52(788.53to902.52)	0.12(-1.17to1.43)	51.16(37.14to65.18)	0.11(-1.11to1.35)
Iodine deficiency	62.75(47.22to78.27)	0.01(-0.23to0.26)	14.21(12.80to15.79)	0.00(-0.17to0.18)
Vitamin A deficiency	290.55(257.14to323.96)	-1.34(-1.45to-1.23)	0.00(0.00to0.00)	-
Dietary iron deficiency	-	-	37.63(25.60to49.65)	0.30(-0.25to0.85)
Other nutritional deficiency	-	-	23.34(13.87to32.81)	3.36(1.66to5.08)

**Ghana**				
Nutritional deficiencies	547.12(501.27to592.96)	-2.79(-3.03to-2.56)	499.24(455.45to543.04)	-2.32(-3.00to-1.64)
Protein-energy malnutrition	393.23(354.37to432.10)	-2.51(-2.74to-2.27)	56.96(42.17to71.75)	-5.27(-5.83to-4.70)
Iodine deficiency	153.88(129.57to178.19)	-3.29(-3.54to-3.05)	50.39(36.48to64.31)	-3.13(-3.37to-2.90)
Vitamin A deficiency	6112.35(5959.12to6265.59)	-3.78(-4.14to-3.43)	4.10(3.37to4.99)	-0.64(-0.77to-0.51)
Dietary iron deficiency	-	-	369.29(331.63to406.96)	-0.67(-1.64to0.31)
Other nutritional deficiency	-	-	18.49(17.88to19.12)	-4.55(-5.05to-4.04)
**Greece**				
Nutritional deficiencies	413.23(373.39to453.08)	-0.18(-1.06to0.71)	73.52(56.71to90.32)	-0.94(-1.54to-0.34)
Protein-energy malnutrition	346.62(310.13to383.11)	-0.17(-1.17to0.84)	15.58(11.77to20.63)	0.05(-0.85to0.96)
Iodine deficiency	66.61(50.62to82.61)	-0.23(-0.45to-0.01)	15.20(11.44to20.20)	-0.30(-0.45to-0.15)
Vitamin A deficiency	591.26(543.60to638.92)	-1.97(-2.03to-1.90)	0.00(0.00to0.00)	-
Dietary iron deficiency	-	-	35.33(23.68to46.98)	-2.28(-2.70to-1.86)
Other nutritional deficiency	-	-	7.40(4.93to11.12)	3.35(0.75to6.01)
**Greenland**				
Nutritional deficiencies	538.70(493.20to584.19)	0.70(0.02to1.39)	247.08(216.27to277.89)	-0.74(-0.78to-0.70)
Protein-energy malnutrition	523.35(478.51to568.19)	0.72(0.02to1.43)	14.82(0.35to629.01)	0.87(0.40to1.35)
Iodine deficiency	15.34(0.39to610.45)	-0.17(-0.20to-0.14)	3.38(0.00to8633.70)	-0.24(-0.30to-0.19)
Vitamin A deficiency	1170.65(1103.59to1237.71)	-2.51(-2.62to-2.40)	0.00(0.00to0.00)	-
Dietary iron deficiency	-	-	222.83(193.57to252.08)	-0.94(-1.00to-0.89)
Other nutritional deficiency	-	-	6.05(0.02to2133.19)	3.76(1.46to6.12)
**Grenada**				
Nutritional deficiencies	567.25(520.56to613.93)	-0.10(-0.19to-0.01)	385.00(346.54to423.46)	-0.17(-0.25to-0.10)
Protein-energy malnutrition	552.51(506.44to598.59)	0.06(-0.04to0.16)	28.84(18.31to39.36)	-2.15(-2.37to-1.93)
Iodine deficiency	14.73(1.06to204.12)	-3.78(-3.87to-3.68)	7.12(0.16to312.21)	-4.26(-4.40to-4.13)
Vitamin A deficiency	3792.20(3671.50to3912.90)	-2.74(-3.00to-2.49)	2.82(0.01to1149.56)	0.27(0.15to0.38)
Dietary iron deficiency	-	-	335.45(299.55to371.35)	0.10(0.05to0.15)
Other nutritional deficiency	-	-	10.77(0.50to233.00)	1.67(0.16to3.21)
**Guam**				
Nutritional deficiencies	920.69(861.22to980.16)	-2.63(-3.14to-2.10)	407.83(368.24to447.41)	0.85(0.69to1.01)
Protein-energy malnutrition	916.59(857.25to975.93)	-2.63(-3.15to-2.11)	28.48(18.02to38.94)	-2.44(-2.93to-1.95)
Iodine deficiency	4.10(0.07to247.07)	-1.10(-1.20to-1.01)	0.73(0.00to12077.49)	-1.29(-1.37to-1.20)
Vitamin A deficiency	2065.44(1976.37to2154.52)	-2.39(-3.06to-1.72)	0.00(0.00to0.00)	-
Dietary iron deficiency	-	-	368.05(330.45to405.66)	1.14(0.96to1.32)
Other nutritional deficiency	-	-	10.56(0.82to135.71)	1.03(-0.77to2.85)
**Guatemala**				

Nutritional deficiencies	714.86(662.45to767.26)	-3.08(-3.44to-2.71)	305.83(271.55to340.11)	-6.35(-7.14to-5.56)
Protein-energy malnutrition	691.61(640.07to743.16)	-3.13(-3.50to-2.76)	117.90(96.62to139.18)	-8.14(-9.00to-7.28)
Iodine deficiency	23.24(13.79to32.69)	-0.65(-0.69to-0.61)	16.24(13.57to19.44)	-7.96(-10.59to-5.26)
Vitamin A deficiency	5683.75(5535.98to5831.51)	-4.36(-4.84to-3.88)	4.06(2.83to5.82)	-0.06(-0.15to0.03)
Dietary iron deficiency	-	-	145.53(121.88to169.17)	-2.80(-3.61to-1.99)
Other nutritional deficiency	-	-	22.10(12.89to31.32)	-5.83(-9.44to-2.09)
**Guinea**				
Nutritional deficiencies	525.38(480.45to570.31)	-2.34(-2.49to-2.20)	459.88(417.85to501.91)	-1.77(-1.91to-1.64)
Protein-energy malnutrition	344.42(308.04to380.79)	-0.77(-0.82to-0.72)	63.88(48.21to79.54)	-2.36(-2.70to-2.02)
Iodine deficiency	180.96(154.60to207.33)	-4.02(-4.30to-3.75)	62.18(46.73to77.64)	-4.38(-4.61to-4.14)
Vitamin A deficiency	10229.38(10031.15to10427.62)	-1.94(-2.23to-1.65)	4.58(3.34to6.28)	-0.67(-0.79to-0.55)
Dietary iron deficiency	-	-	311.19(276.61to345.76)	-0.64(-0.82to-0.45)
Other nutritional deficiency	-	-	18.05(16.87to19.31)	-1.55(-1.94to-1.16)
**Guinea Bissau**				
Nutritional deficiencies	595.46(547.64to643.29)	-0.76(-0.95to-0.58)	590.83(543.19to638.47)	-0.28(-0.85to0.28)
Protein-energy malnutrition	344.02(307.66to380.37)	-0.05(-0.06to-0.03)	86.76(68.50to105.01)	-3.16(-3.35to-2.96)
Iodine deficiency	251.45(220.37to282.53)	-1.52(-1.92to-1.12)	83.21(65.33to101.09)	-1.52(-1.98to-1.06)
Vitamin A deficiency	11176.78(10969.57to11384.00)	-1.37(-1.64to-1.10)	4.89(1.74to13.73)	-0.95(-1.31to-0.58)
Dietary iron deficiency	-	-	394.00(355.10to432.91)	1.87(0.80to2.95)
Other nutritional deficiency	-	-	21.97(12.78to31.15)	-3.13(-3.45to-2.80)
**Guyana**				
Nutritional deficiencies	500.59(456.73to544.44)	-3.26(-3.62to-2.89)	686.34(634.99to737.69)	-2.35(-2.56to-2.14)
Protein-energy malnutrition	474.95(432.24to517.67)	-3.37(-3.76to-2.97)	80.34(62.77to97.91)	-4.64(-5.34to-3.93)
Iodine deficiency	25.63(15.71to35.56)	-1.39(-1.63to-1.14)	14.20(5.54to36.39)	-1.38(-1.50to-1.26)
Vitamin A deficiency	4901.62(4764.40to5038.84)	-2.01(-2.47to-1.56)	2.89(0.36to23.28)	0.38(0.28to0.48)
Dietary iron deficiency	-	-	568.97(522.21to615.72)	-1.99(-2.11to-1.88)
Other nutritional deficiency	-	-	19.95(9.02to44.14)	-0.76(-3.02to1.54)
**Haiti**				
Nutritional deficiencies	453.75(412.00to495.50)	0.31(0.26to0.37)	762.39(708.27to816.51)	-1.45(-1.60to-1.30)
Protein-energy malnutrition	410.55(370.84to450.26)	0.37(0.25to0.49)	77.44(60.19to94.69)	-2.65(-3.19to-2.11)
Iodine deficiency	43.20(30.32to56.08)	-0.08(-0.49to0.33)	32.61(21.42to43.81)	-0.12(-0.49to0.26)
Vitamin A deficiency	11526.48(11316.05to11736.90)	-2.33(-2.42to-2.25)	3.44(2.15to5.51)	-0.35(-0.62to-0.07)
Dietary iron deficiency	-	-	634.29(584.92to683.65)	-1.34(-1.61to-1.07)
Other nutritional deficiency	-	-	14.61(11.62to18.36)	-0.72(-1.46to0.03)
**Honduras**				
Nutritional deficiencies	707.72(655.58to759.86)	-1.82(-1.91to-1.72)	240.04(209.68to270.41)	-1.94(-2.25to-1.63)

Protein-energy malnutrition	680.97(629.82to732.12)	-1.86(-1.96to-1.75)	54.08(39.67to68.50)	-4.13(-4.38to-3.88)
Iodine deficiency	26.75(16.61to36.89)	-0.87(-0.94to-0.79)	20.84(11.89to29.79)	-8.18(-10.43to-5.88)
Vitamin A deficiency	6010.59(5858.64to6162.55)	-3.23(-3.73to-2.72)	2.72(1.50to4.93)	-0.06(-0.25to0.14)
Dietary iron deficiency	-	-	151.71(127.57to175.86)	0.05(-0.66to0.76)
Other nutritional deficiency	-	-	10.68(7.91to14.42)	-3.52(-4.11to-2.93)
**Hungary**				
Nutritional deficiencies	301.97(267.91to336.03)	0.92(0.86to0.99)	150.20(126.18to174.23)	0.09(-0.30to0.48)
Protein-energy malnutrition	288.85(255.54to322.16)	1.12(1.08to1.17)	10.92(7.78to15.32)	1.77(1.34to2.20)
Iodine deficiency	13.12(9.63to17.87)	-2.21(-2.47to-1.94)	3.25(1.75to6.05)	-2.69(-3.04to-2.35)
Vitamin A deficiency	9560.94(9369.29to9752.59)	-2.14(-2.28to-2.00)	0.00(0.00to0.00)	-
Dietary iron deficiency	-	-	130.80(108.39to153.22)	-0.08(-0.50to0.34)
Other nutritional deficiency	-	-	5.23(3.20to8.53)	4.23(2.63to5.86)
**Iceland**				
Nutritional deficiencies	1027.66(964.82to1090.49)	0.98(0.92to1.04)	83.64(65.71to101.56)	1.32(0.92to1.73)
Protein-energy malnutrition	999.83(937.85to1061.81)	1.02(0.95to1.08)	43.49(30.56to56.41)	1.24(1.18to1.29)
Iodine deficiency	27.83(17.49to38.16)	-0.21(-0.28to-0.14)	5.55(0.47to65.70)	-0.21(-0.31to-0.10)
Vitamin A deficiency	369.62(331.94to407.30)	-2.15(-2.36to-1.94)	0.00(0.00to0.00)	-
Dietary iron deficiency	-	-	14.79(3.25to67.21)	-1.77(-2.34to-1.20)
Other nutritional deficiency	-	-	19.81(5.36to73.28)	4.75(2.56to6.99)
**India**				
Nutritional deficiencies	2398.26(2302.28to2494.25)	-2.02(-2.13to-1.92)	1161.03(1094.25to1227.82)	-0.91(-1.11to-0.72)
Protein-energy malnutrition	2011.79(1923.88to2099.70)	-1.85(-1.96to-1.74)	63.28(47.69to78.87)	-5.03(-6.01to-4.03)
Iodine deficiency	386.47(347.94to425.01)	-2.66(-2.92to-2.40)	126.22(104.20to148.24)	-1.68(-2.13to-1.22)
Vitamin A deficiency	5476.10(5331.06to5621.14)	-3.76(-4.05to-3.47)	6.63(6.41to6.85)	-1.56(-1.66to-1.46)
Dietary iron deficiency	0.00(0.00to0.00)	-	915.77(856.46to975.09)	0.13(0.03to0.22)
Other nutritional deficiency	0.00(0.00to0.00)	-	49.13(35.39to62.87)	-7.80(-9.14to-6.45)
**Indonesia**				
Nutritional deficiencies	2257.87(2164.73to2351.00)	-0.66(-1.50to0.19)	516.37(471.83to560.91)	-2.19(-2.32to-2.05)
Protein-energy malnutrition	2219.62(2127.27to2311.96)	-0.61(-1.49to0.28)	99.97(80.37to119.57)	-2.64(-3.18to-2.09)
Iodine deficiency	38.25(26.13to50.37)	-2.04(-2.42to-1.65)	7.62(7.11to8.18)	-14.25(-17.84to-10.51)
Vitamin A deficiency	5161.04(5020.23to5301.84)	-4.06(-4.22to-3.89)	10.70(10.09to11.36)	-1.74(-2.06to-1.41)
Dietary iron deficiency	0.00(0.00to0.00)	-	358.32(321.22to395.42)	-1.86(-1.98to-1.74)
Other nutritional deficiency	0.00(0.00to0.00)	-	39.75(27.39to52.11)	1.36(0.21to2.53)
**Iran**				
Nutritional deficiencies	880.83(822.66to939.00)	1.51(1.48to1.55)	117.12(95.91to138.33)	-0.90(-1.33to-0.47)

Protein-energy malnutrition	861.29(803.77to918.81)	1.63(1.59to1.66)	21.42(12.35to30.49)	0.05(-0.21to0.32)
Iodine deficiency	19.55(18.10to21.11)	-2.01(-2.09to-1.94)	9.23(8.25to10.32)	-5.52(-5.71to-5.32)
Vitamin A deficiency	980.32(918.95to1041.69)	-6.58(-7.86to-5.27)	0.00(0.00to0.00)	-
Dietary iron deficiency	0.00(0.00to0.00)	-	76.63(59.47to93.78)	-0.75(-1.35to-0.14)
Other nutritional deficiency	0.00(0.00to0.00)	-	9.84(8.83to10.97)	4.31(2.45to6.20)
**Iraq**				
Nutritional deficiencies	700.30(648.43to752.17)	1.91(1.78to2.05)	251.16(220.10to282.22)	-0.98(-1.28to-0.68)
Protein-energy malnutrition	653.45(603.35to703.55)	2.37(2.23to2.51)	21.19(12.17to30.21)	-0.32(-0.83to0.19)
Iodine deficiency	46.85(33.44to60.27)	-1.85(-1.96to-1.73)	35.45(23.78to47.12)	-3.13(-3.30to-2.96)
Vitamin A deficiency	2525.37(2426.87to2623.87)	-4.38(-4.70to-4.05)	3.82(2.99to4.89)	-0.93(-1.05to-0.81)
Dietary iron deficiency	-	-	181.20(154.82to207.59)	-0.62(-0.99to-0.24)
Other nutritional deficiency	-	-	9.50(8.12to11.11)	1.99(0.87to3.12)
**Ireland**				
Nutritional deficiencies	904.65(845.70to963.60)	0.86(0.80to0.93)	99.78(80.21to119.36)	-0.75(-1.12to-0.38)
Protein-energy malnutrition	842.31(785.43to899.20)	0.95(0.88to1.02)	37.64(25.61to49.66)	1.04(0.95to1.13)
Iodine deficiency	62.34(46.86to77.81)	-0.23(-0.26to-0.21)	14.06(9.38to21.07)	-0.27(-0.32to-0.21)
Vitamin A deficiency	360.83(323.60to398.06)	-1.89(-2.17to-1.61)	0.00(0.00to0.00)	-
Dietary iron deficiency	-	-	30.71(19.85to41.57)	-4.14(-4.64to-3.65)
Other nutritional deficiency	-	-	17.38(12.08to25.02)	4.16(2.07to6.30)
**Israel**				
Nutritional deficiencies	1016.86(954.36to1079.36)	0.53(0.42to0.64)	116.22(95.09to137.36)	-0.48(-0.69to-0.27)
Protein-energy malnutrition	951.00(890.56to1011.44)	0.57(0.45to0.69)	41.95(29.26to54.65)	0.77(0.66to0.87)
Iodine deficiency	65.86(49.95to81.77)	0.05(-0.03to0.13)	15.02(11.28to19.99)	0.04(-0.04to0.12)
Vitamin A deficiency	1940.80(1854.45to2027.14)	-1.50(-1.75to-1.25)	0.00(0.00to0.00)	-
Dietary iron deficiency	-	-	40.03(27.63to52.43)	-2.94(-3.20to-2.68)
Other nutritional deficiency	-	-	19.22(14.93to24.76)	4.05(2.01to6.12)
**Italy**				
Nutritional deficiencies	1359.68(1287.41to1431.96)	-0.03(-0.25to0.19)	166.39(141.10to191.67)	-1.52(-2.23to-0.80)
Protein-energy malnutrition	1097.75(1032.81to1162.69)	1.28(1.06to1.49)	48.97(35.26to62.69)	1.56(1.36to1.77)
Iodine deficiency	261.94(230.22to293.66)	-4.25(-5.68to-2.79)	64.45(48.71to80.18)	-4.11(-5.85to-2.35)
Vitamin A deficiency	957.91(897.25to1018.57)	-1.17(-1.29to-1.06)	0.00(0.00to0.00)	-
Dietary iron deficiency	0.00(0.00to0.00)	-	30.15(19.39to40.91)	-3.12(-3.55to-2.69)
Other nutritional deficiency	0.00(0.00to0.00)	-	22.82(13.45to32.18)	4.60(2.43to6.81)
**Jamaica**				
Nutritional deficiencies	484.38(441.24to527.51)	-3.81(-4.34to-3.27)	461.12(419.03to503.21)	-1.38(-2.05to-0.70)
Protein-energy	464.55(422.30to506.79)	-3.89(-4.45to-3.31)	29.79(19.09to40.49)	-6.60(-8.17to-5.01)

malnutrition				
Iodine deficiency	19.83(13.09to30.04)	-2.25(-2.49to-2.02)	10.32(5.80to18.35)	-2.26(-2.38to-2.15)
Vitamin A deficiency	2872.93(2767.87to2977.98)	-3.29(-3.75to-2.82)	2.76(0.91to8.41)	0.47(0.30to0.63)
Dietary iron deficiency	-	-	407.46(367.90to447.03)	-0.74(-1.37to-0.10)
Other nutritional deficiency	-	-	10.79(6.14to18.95)	-2.35(-3.31to-1.38)
**Japan**				
Nutritional deficiencies	523.27(478.44to568.11)	0.60(0.20to1.00)	253.35(222.16to284.55)	-1.40(-1.64to-1.16)
Protein-energy malnutrition	504.98(460.93to549.02)	0.65(0.23to1.06)	18.00(16.65to19.45)	0.60(0.24to0.95)
Iodine deficiency	18.29(16.94to19.76)	-0.50(-0.52to-0.48)	4.10(3.48to4.82)	-0.51(-0.55to-0.48)
Vitamin A deficiency	788.29(733.26to843.32)	-0.69(-0.81to-0.56)	0.00(0.00to0.00)	
Dietary iron deficiency	0.00(0.00to0.00)	-	223.93(194.60to253.26)	-1.67(-1.92to-1.43)
Other nutritional deficiency	0.00(0.00to0.00)	-	7.33(6.49to8.28)	3.46(1.81to5.13)
**Jordan**				
Nutritional deficiencies	813.46(757.56to869.36)	0.52(0.09to0.96)	348.14(311.57to384.71)	-2.53(-2.74to-2.31)
Protein-energy malnutrition	784.89(729.98to839.80)	0.61(0.16to1.05)	11.06(8.30to14.75)	-1.15(-1.67to-0.62)
Iodine deficiency	28.57(18.09to39.05)	-1.44(-1.65to-1.23)	16.98(13.46to21.41)	-2.09(-2.19to-2.00)
Vitamin A deficiency	3855.87(3734.17to3977.58)	-3.78(-4.61to-2.95)	3.06(1.77to5.29)	-0.75(-0.87to-0.63)
Dietary iron deficiency	-	-	311.73(277.12to346.33)	-2.68(-2.96to-2.39)
Other nutritional deficiency	-	-	5.31(3.51to8.05)	1.52(-0.54to3.62)
**Kazakhstan**				
Nutritional deficiencies	430.68(390.00to471.36)	1.48(0.64to2.32)	550.80(504.80to596.79)	-2.08(-2.60to-1.56)
Protein-energy malnutrition	406.84(367.30to446.37)	2.05(1.18to2.93)	23.58(14.06to33.10)	3.33(2.85to3.81)
Iodine deficiency	23.84(14.27to33.41)	-3.71(-4.06to-3.36)	9.67(7.58to12.33)	-9.78(-11.74to-7.78)
Vitamin A deficiency	2874.07(2769.00to2979.15)	-2.88(-3.03to-2.73)	0.00(0.00to0.00)	-
Dietary iron deficiency	-	-	505.68(461.60to549.75)	-2.09(-2.61to-1.58)
Other nutritional deficiency	-	-	11.87(9.53to14.78)	5.16(3.44to6.91)
**Kenya**				
Nutritional deficiencies	766.57(712.31to820.84)	1.24(0.18to2.31)	471.85(429.28to514.43)	0.06(-0.22to0.35)
Protein-energy malnutrition	688.69(637.25to740.13)	1.45(0.24to2.67)	109.10(88.63to129.57)	-2.64(-3.32to-1.96)
Iodine deficiency	77.88(60.59to95.18)	-0.42(-0.53to-0.32)	18.19(16.40to20.19)	0.64(0.15to1.13)
Vitamin A deficiency	27936.94(27609.34to28264.54)	-1.95(-2.08to-1.82)	4.14(3.33to5.15)	-1.68(-1.96to-1.39)
Dietary iron deficiency	0.00(0.00to0.00)	-	323.44(288.19to358.69)	2.12(1.94to2.30)
Other nutritional deficiency	0.00(0.00to0.00)	-	16.98(15.25to18.91)	-2.52(-3.15to-1.88)
**Kiribati**				
Nutritional deficiencies	779.08(724.37to833.79)	-0.93(-1.25to-0.60)	894.09(835.49to952.70)	-0.61(-0.82to-0.40)
Protein-energy malnutrition	772.21(717.74to826.67)	-0.91(-1.25to-0.57)	103.50(83.56to123.44)	-2.87(-3.40to-2.34)

Iodine deficiency	6.87(0.21to228.51)	-1.94(-2.57to-1.31)	3.84(0.04to417.19)	-2.42(-2.93to-1.91)
Vitamin A deficiency	13811.21(13580.87to14041.55)	-0.27(-0.85to0.31)	2.31(0.01to975.54)	-1.01(-1.09to-0.94)
Dietary iron deficiency	-	-	775.61(721.03to830.20)	-0.20(-0.54to0.14)
Other nutritional deficiency	-	-	8.83(0.40to194.28)	0.68(-0.27to1.63)
**Kuwait**				
Nutritional deficiencies	847.83(790.76to904.90)	0.54(0.22to0.86)	348.18(311.61to384.75)	-1.38(-1.61to-1.14)
Protein-energy malnutrition	825.26(768.95to881.57)	0.59(0.26to0.92)	19.37(14.07to26.66)	0.56(0.37to0.75)
Iodine deficiency	22.57(13.26to31.88)	-0.96(-1.06to-0.87)	5.85(3.27to10.47)	-1.36(-1.38to-1.34)
Vitamin A deficiency	768.83(714.48to823.17)	-4.37(-5.20to-3.54)	3.65(1.75to7.62)	-0.17(-0.31to-0.03)
Dietary iron deficiency	-	-	310.18(275.66to344.70)	-1.63(-1.89to-1.37)
Other nutritional deficiency	-	-	9.13(5.73to14.54)	3.65(1.73to5.61)
**Kyrgyzstan**				
Nutritional deficiencies	310.98(276.42to345.55)	1.22(0.98to1.47)	546.32(500.51to592.13)	-2.11(-2.26to-1.96)
Protein-energy malnutrition	289.38(256.04to322.73)	1.34(1.06to1.61)	8.45(5.49to13.01)	-3.10(-4.13to-2.06)
Iodine deficiency	21.60(12.49to30.71)	0.02(-0.08to0.11)	8.21(5.30to12.71)	0.14(0.00to0.28)
Vitamin A deficiency	3571.86(3454.72to3689.00)	-0.87(-1.38to-0.36)	3.96(2.11to7.44)	-0.56(-0.77to-0.34)
Dietary iron deficiency	-	-	522.19(477.40to566.98)	-2.12(-2.26to-1.98)
Other nutritional deficiency	-	-	3.50(1.79to6.85)	-3.23(-5.45to-0.95)
**Lao People s Democratic Republic**				
Nutritional deficiencies	990.49(928.81to1052.18)	0.71(0.63to0.78)	484.13(441.01to527.26)	-1.81(-1.96to-1.67)
Protein-energy malnutrition	948.82(888.45to1009.19)	0.86(0.76to0.95)	47.01(33.57to60.45)	-5.37(-5.59to-5.14)
Iodine deficiency	41.67(29.02to54.33)	-1.42(-1.76to-1.08)	15.53(11.56to20.86)	-16.39(-20.77to-11.77)
Vitamin A deficiency	8530.04(8349.01to8711.06)	-4.06(-4.26to-3.86)	1.05(0.34to3.27)	-0.47(-0.65to-0.30)
Dietary iron deficiency	-	-	408.43(368.82to448.04)	-0.38(-0.91to0.15)
Other nutritional deficiency	-	-	12.12(8.67to16.93)	0.87(-0.30to2.05)
**Latvia**				
Nutritional deficiencies	280.46(247.64to313.29)	0.25(-0.53to1.04)	152.27(128.08to176.45)	-1.45(-1.70to-1.20)
Protein-energy malnutrition	274.06(241.61to306.50)	0.30(-0.52to1.12)	13.12(6.41to26.84)	0.25(-0.49to1.01)
Iodine deficiency	6.41(2.30to17.85)	-1.21(-1.51to-0.92)	3.84(1.02to14.42)	-1.49(-1.72to-1.26)
Vitamin A deficiency	1166.93(1099.98to1233.89)	-4.27(-4.63to-3.90)	0.00(0.00to0.00)	-
Dietary iron deficiency	-	-	124.62(102.74to146.50)	-1.83(-2.05to-1.60)
Other nutritional deficiency	-	-	10.69(4.84to23.64)	3.11(2.06to4.16)
**Lebanon**				
Nutritional deficiencies	786.61(731.64to841.58)	1.52(0.75to2.29)	104.70(84.65to124.76)	-3.28(-3.47to-3.10)
Protein-energy	736.19(683.01to789.37)	1.72(0.90to2.55)	27.26(17.03to37.49)	1.32(0.54to2.10)

malnutrition				
Iodine deficiency	50.42(36.50to64.34)	-0.90(-1.05to-0.75)	22.10(12.89to31.31)	-1.27(-1.34to-1.21)
Vitamin A deficiency	1550.02(1472.85to1627.18)	-4.67(-4.94to-4.39)	0.00(0.00to0.00)	-
Dietary iron deficiency	-	-	43.02(30.16to55.87)	-6.52(-6.61to-6.44)
Other nutritional deficiency	-	-	12.32(8.25to18.41)	4.86(2.18to7.60)
**Lesotho**				
Nutritional deficiencies	510.67(466.38to554.96)	-2.02(-2.75to-1.29)	377.79(339.69to415.88)	0.31(-0.31to0.93)
Protein-energy malnutrition	296.83(263.07to330.60)	0.07(-0.08to0.22)	97.57(78.21to116.93)	3.38(2.09to4.69)
Iodine deficiency	213.84(185.17to242.50)	-4.32(-5.57to-3.05)	75.75(58.69to92.81)	-1.59(-1.73to-1.46)
Vitamin A deficiency	10019.70(9823.51to10215.90)	-1.83(-2.10to-1.56)	5.55(2.23to13.83)	-0.82(-1.00to-0.64)
Dietary iron deficiency	-	-	188.26(161.37to215.16)	-0.46(-1.11to0.19)
Other nutritional deficiency	-	-	10.65(5.51to20.58)	3.65(2.11to5.21)
**Liberia**				
Nutritional deficiencies	428.87(388.28to469.46)	-1.45(-1.55to-1.35)	335.99(300.07to371.92)	-3.94(-4.35to-3.52)
Protein-energy malnutrition	371.91(334.11to409.71)	-1.11(-1.17to-1.04)	78.43(61.07to95.78)	-4.55(-5.07to-4.03)
Iodine deficiency	56.96(42.17to71.75)	-3.34(-3.80to-2.88)	25.22(15.38to35.06)	-5.22(-5.77to-4.66)
Vitamin A deficiency	3571.31(3454.18to3688.44)	-4.19(-4.89to-3.48)	4.05(1.97to8.31)	-1.11(-1.36to-0.86)
Dietary iron deficiency	-	-	204.54(176.51to232.57)	-3.39(-3.76to-3.03)
Other nutritional deficiency	-	-	23.76(14.20to33.31)	-4.79(-5.28to-4.30)
**Libya**				
Nutritional deficiencies	772.83(718.34to827.31)	1.22(1.14to1.31)	320.77(285.67to355.88)	-0.05(-0.36to0.26)
Protein-energy malnutrition	726.42(673.59to779.24)	1.39(1.27to1.51)	20.15(11.35to28.95)	0.71(-0.13to1.55)
Iodine deficiency	46.41(33.06to59.76)	-1.33(-1.86to-0.80)	34.72(23.17to46.27)	-1.74(-2.35to-1.12)
Vitamin A deficiency	1590.39(1512.22to1668.55)	-5.06(-5.36to-4.76)	4.15(2.35to7.35)	-0.61(-1.00to-0.22)
Dietary iron deficiency	-	-	252.90(221.73to284.07)	-0.04(-0.44to0.37)
Other nutritional deficiency	-	-	8.86(5.99to13.10)	3.77(2.13to5.43)
**Lithuania**				
Nutritional deficiencies	288.81(255.50to322.12)	0.32(-0.58to1.23)	164.85(139.68to190.02)	-0.22(-0.56to0.12)
Protein-energy malnutrition	282.71(249.76to315.67)	0.36(-0.57to1.31)	12.94(7.17to23.35)	0.57(-0.25to1.40)
Iodine deficiency	6.10(2.58to14.41)	-1.09(-1.36to-0.82)	3.61(1.18to11.04)	-1.25(-1.47to-1.03)
Vitamin A deficiency	1056.81(993.10to1120.53)	-4.05(-4.25to-3.85)	0.00(0.00to0.00)	-
Dietary iron deficiency	-	-	141.75(118.41to165.08)	-0.38(-0.80to0.04)
Other nutritional deficiency	-	-	6.56(2.86to15.02)	3.32(1.76to4.91)
**Luxembourg**				
Nutritional deficiencies	1087.22(1022.59to1151.85)	1.00(0.96to1.05)	108.49(88.07to128.90)	0.15(-0.13to0.42)
Protein-energy malnutrition	1026.31(963.52to1089.10)	1.06(1.01to1.10)	44.26(31.22to57.30)	1.28(1.23to1.34)

Iodine deficiency	60.91(45.61to76.20)	0.12(0.04to0.20)	13.74(4.37to43.17)	0.06(-0.06to0.18)
Vitamin A deficiency	332.66(296.91to368.41)	-1.13(-1.34to-0.93)	0.00(0.00to0.00)	-
Dietary iron deficiency	-	-	30.44(19.63to41.25)	-3.18(-3.80to-2.57)
Other nutritional deficiency	-	-	20.05(11.27to28.82)	4.86(2.64to7.12)
**Madagascar**				
Nutritional deficiencies	650.70(600.70to700.69)	-2.74(-2.85to-2.63)	752.16(698.41to805.92)	-1.67(-2.02to-1.32)
Protein-energy malnutrition	498.60(454.83to542.36)	-3.25(-3.56to-2.94)	371.20(333.43to408.96)	-1.91(-2.34to-1.49)
Iodine deficiency	152.10(127.93to176.27)	-0.94(-1.53to-0.34)	55.90(41.25to70.56)	0.19(0.15to0.22)
Vitamin A deficiency	15140.00(14898.83to15381.17)	-2.34(-3.04to-1.63)	3.28(2.32to4.63)	-0.83(-0.95to-0.72)
Dietary iron deficiency	-	-	271.22(238.94to303.50)	-0.90(-1.94to0.15)
Other nutritional deficiency	-	-	50.57(36.63to64.50)	-3.60(-4.06to-3.14)
**Malawi**				
Nutritional deficiencies	1231.60(1162.82to1300.38)	1.42(1.06to1.79)	530.45(485.31to575.60)	-3.64(-3.85to-3.44)
Protein-energy malnutrition	1067.82(1003.77to1131.87)	2.26(1.74to2.79)	108.43(88.02to128.84)	-6.77(-7.72to-5.80)
Iodine deficiency	163.78(138.69to188.86)	-2.01(-2.16to-1.87)	60.91(45.61to76.21)	-2.65(-2.80to-2.49)
Vitamin A deficiency	29626.67(29289.31to29964.03)	-2.49(-2.87to-2.10)	3.81(2.60to5.58)	-1.47(-1.58to-1.37)
Dietary iron deficiency	-	-	334.30(298.46to370.13)	-1.88(-2.40to-1.36)
Other nutritional deficiency	-	-	23.00(13.60to32.40)	-6.92(-7.79to-6.05)
**Malaysia**				
Nutritional deficiencies	1158.21(1091.51to1224.91)	-0.77(-1.10to-0.44)	408.19(368.59to447.79)	-2.43(-2.54to-2.32)
Protein-energy malnutrition	1062.51(998.62to1126.40)	-0.22(-0.52to0.08)	23.48(13.98to32.97)	-0.28(-0.43to-0.12)
Iodine deficiency	95.70(76.53to114.88)	-3.49(-4.07to-2.92)	28.01(17.64to38.38)	-6.51(-6.96to-6.07)
Vitamin A deficiency	610.28(561.87to658.70)	-3.98(-4.64to-3.32)	0.00(0.00to0.00)	-
Dietary iron deficiency	-	-	346.36(309.88to382.84)	-2.21(-2.30to-2.12)
Other nutritional deficiency	-	-	10.35(8.68to12.33)	3.37(1.17to5.61)
**Maldives**				
Nutritional deficiencies	1941.27(1854.91to2027.62)	0.77(-0.19to1.74)	443.38(402.11to484.65)	-4.17(-4.47to-3.88)
Protein-energy malnutrition	1916.05(1830.25to2001.84)	0.93(-0.06to1.93)	43.45(30.53to56.37)	-3.17(-4.72to-1.59)
Iodine deficiency	25.22(15.38to35.06)	-4.98(-5.33to-4.63)	9.14(1.84to45.38)	-13.54(-16.22to-10.77)
Vitamin A deficiency	2735.39(2632.88to2837.90)	-5.81(-7.19to-4.41)	0.85(0.00to162.16)	-0.14(-0.29to0.02)
Dietary iron deficiency	-	-	369.23(331.56to406.89)	-4.27(-4.81to-3.72)
Other nutritional deficiency	-	-	20.71(11.79to29.63)	2.25(0.85to3.67)
**Mali**				
Nutritional deficiencies	476.22(433.45to518.99)	-0.79(-0.96to-0.62)	980.59(919.21to1041.96)	-1.93(-2.28to-1.57)
Protein-energy malnutrition	408.97(369.34to448.61)	-0.45(-0.60to-0.29)	351.14(314.41to387.86)	-4.06(-4.89to-3.23)

Iodine deficiency	67.24(51.17to83.32)	-2.40(-2.62to-2.19)	27.84(17.50to38.19)	-2.90(-3.13to-2.67)
Vitamin A deficiency	13853.66(13622.97to14084.36)	-2.28(-2.57to-1.98)	5.09(3.71to7.00)	-0.94(-1.63to-0.25)
Dietary iron deficiency	-	-	523.82(478.96to568.68)	-0.20(-1.07to0.67)
Other nutritional deficiency	-	-	72.69(55.98to89.40)	-3.69(-4.43to-2.95)
**Malta**				
Nutritional deficiencies	925.37(865.75to984.99)	0.42(0.32to0.51)	113.99(93.06to134.91)	0.25(-0.10to0.61)
Protein-energy malnutrition	858.99(801.55to916.44)	0.47(0.36to0.58)	38.14(26.03to50.24)	0.67(0.56to0.79)
Iodine deficiency	66.38(50.41to82.35)	-0.26(-0.38to-0.14)	15.15(3.85to59.54)	-0.35(-0.38to-0.31)
Vitamin A deficiency	575.01(528.01to622.01)	-2.14(-2.18to-2.09)	0.00(0.00to0.00)	-
Dietary iron deficiency	-	-	42.98(30.13to55.83)	-0.83(-1.22to-0.44)
Other nutritional deficiency	-	-	17.72(5.00to62.82)	3.96(1.96to6.00)
**Marshall Islands**				
Nutritional deficiencies	643.68(593.95to693.40)	-0.37(-0.55to-0.18)	497.43(453.71to541.14)	-0.38(-0.68to-0.07)
Protein-energy malnutrition	638.02(588.52to687.53)	-0.34(-0.54to-0.15)	44.89(31.76to58.03)	-2.17(-2.33to-2.01)
Iodine deficiency	5.65(0.02to1571.87)	-2.31(-2.92to-1.69)	2.36(0.00to14377.65)	-3.21(-3.94to-2.48)
Vitamin A deficiency	14210.63(13976.99to14444.28)	-2.88(-3.11to-2.65)	2.07(0.00to22660.12)	-1.38(-1.56to-1.21)
Dietary iron deficiency	-	-	440.71(399.57to481.86)	-0.16(-0.49to0.18)
Other nutritional deficiency	-	-	7.40(0.05to1012.67)	3.04(1.14to4.97)
**Mauritania**				
Nutritional deficiencies	842.04(785.17to898.92)	-1.37(-3.19to0.49)	551.99(505.94to598.04)	-2.56(-2.81to-2.31)
Protein-energy malnutrition	603.99(555.82to652.16)	-1.62(-3.96to0.78)	94.23(75.20to113.25)	-8.35(-10.08to-6.59)
Iodine deficiency	238.05(207.81to268.29)	-0.89(-1.65to-0.13)	74.78(57.83to91.73)	-2.44(-2.78to-2.09)
Vitamin A deficiency	4435.09(4304.56to4565.62)	-2.14(-2.96to-1.32)	4.05(1.83to8.97)	-0.95(-1.09to-0.81)
Dietary iron deficiency	-	-	361.93(324.64to399.22)	-0.39(-0.61to-0.16)
Other nutritional deficiency	-	-	17.00(11.53to25.08)	-6.37(-7.60to-5.13)
**Mauritius**				
Nutritional deficiencies	955.10(894.53to1015.68)	-0.52(-0.82to-0.22)	231.80(201.96to261.64)	-0.85(-0.94to-0.75)
Protein-energy malnutrition	914.50(855.23to973.77)	-0.41(-0.74to-0.08)	21.25(12.22to30.29)	2.69(1.87to3.51)
Iodine deficiency	40.61(28.12to53.10)	-2.32(-2.53to-2.11)	14.28(6.65to30.65)	-10.28(-12.78to-7.71)
Vitamin A deficiency	1331.02(1259.51to1402.52)	-4.82(-6.03to-3.60)	1.11(0.07to17.21)	-0.01(-0.14to0.12)
Dietary iron deficiency	-	-	187.66(160.81to214.51)	-0.65(-0.99to-0.32)
Other nutritional deficiency	-	-	7.50(2.61to21.51)	2.63(1.03to4.25)
**Mexico**				
Nutritional deficiencies	1191.37(1123.72to1259.02)	-0.68(-1.50to0.14)	229.49(199.80to259.19)	-0.91(-1.43to-0.39)
Protein-energy malnutrition	1156.06(1089.42to1222.70)	-0.67(-1.54to0.20)	70.35(53.91to86.79)	-2.17(-3.15to-1.18)

Iodine deficiency	35.31(23.66to46.96)	-1.03(-1.57to-0.50)	19.44(18.24to20.71)	-2.51(-3.32to-1.69)
Vitamin A deficiency	4366.51(4236.99to4496.02)	-1.78(-2.04to-1.52)	5.20(4.60to5.88)	-0.40(-0.55to-0.26)
Dietary iron deficiency	0.00(0.00to0.00)	-	112.14(91.38to132.89)	0.64(0.52to0.77)
Other nutritional deficiency	0.00(0.00to0.00)	-	22.36(13.09to31.63)	-2.30(-3.32to-1.27)
**Micronesia**				
Nutritional deficiencies	697.92(646.14to749.70)	0.32(-0.05to0.70)	561.18(514.75to607.61)	-0.55(-0.62to-0.47)
Protein-energy malnutrition	692.07(640.51to743.63)	0.35(-0.02to0.73)	40.55(28.07to53.04)	-1.62(-1.92to-1.31)
Iodine deficiency	5.85(0.09to370.68)	-2.18(-2.68to-1.68)	2.45(0.00to1489.10)	-3.40(-3.59to-3.21)
Vitamin A deficiency	25867.54(25552.30to26182.77)	-1.66(-1.95to-1.36)	1.95(0.00to2577.92)	-1.73(-1.86to-1.60)
Dietary iron deficiency	-	-	508.25(464.06to552.44)	-0.48(-0.57to-0.39)
Other nutritional deficiency	-	-	7.98(0.23to278.42)	3.73(1.43to6.09)
**Monaco**				
Nutritional deficiencies	879.88(821.74to938.02)	0.43(0.40to0.45)	93.50(74.55to112.46)	0.53(0.05to1.01)
Protein-energy malnutrition	822.03(765.84to878.23)	0.43(0.40to0.46)	36.21(24.42to48.01)	0.66(0.64to0.69)
Iodine deficiency	57.85(42.94to72.76)	0.33(0.25to0.42)	12.94(0.06to2895.31)	0.34(0.23to0.44)
Vitamin A deficiency	275.44(242.91to307.97)	-0.99(-1.08to-0.89)	0.00(0.00to0.00)	-
Dietary iron deficiency	-	-	27.77(17.44to38.09)	-1.21(-1.79to-0.63)
Other nutritional deficiency	-	-	16.58(0.14to1974.31)	4.11(1.96to6.31)
**Mongolia**				
Nutritional deficiencies	307.39(273.03to341.76)	1.04(0.66to1.42)	338.71(302.64to374.78)	-5.42(-5.85to-4.98)
Protein-energy malnutrition	286.11(252.96to319.27)	1.59(1.14to2.04)	11.38(6.83to18.95)	1.08(0.71to1.44)
Iodine deficiency	21.28(12.24to30.32)	-3.16(-3.19to-3.13)	15.58(10.08to24.09)	-5.77(-5.94to-5.60)
Vitamin A deficiency	1013.90(951.49to1076.31)	-3.39(-3.57to-3.21)	3.68(1.50to9.02)	-1.64(-1.79to-1.49)
Dietary iron deficiency	-	-	302.59(268.50to336.68)	-5.64(-6.11to-5.16)
Other nutritional deficiency	-	-	5.48(2.63to11.42)	3.63(1.59to5.72)
**Montenegro**				
Nutritional deficiencies	743.70(690.25to797.15)	2.12(2.05to2.19)	180.55(154.21to206.88)	-0.54(-0.79to-0.28)
Protein-energy malnutrition	730.31(677.34to783.28)	2.22(2.14to2.30)	27.39(17.13to37.65)	2.22(2.18to2.27)
Iodine deficiency	13.39(4.14to43.35)	-1.66(-1.91to-1.42)	3.36(0.32to35.06)	-1.92(-2.26to-1.59)
Vitamin A deficiency	9175.40(8987.65to9363.14)	-3.23(-3.53to-2.94)	0.00(0.00to0.00)	-
Dietary iron deficiency	-	-	136.90(113.97to159.83)	-1.29(-1.56to-1.01)
Other nutritional deficiency	-	-	12.90(3.90to42.69)	5.21(3.20to7.27)
**Morocco**				
Nutritional deficiencies	720.92(668.29to773.54)	0.69(0.60to0.78)	373.95(336.05to411.85)	-0.71(-0.76to-0.66)
Protein-energy malnutrition	668.06(617.40to718.72)	0.97(0.88to1.05)	26.43(16.36to36.51)	-0.81(-1.17to-0.44)
Iodine deficiency	52.86(38.61to67.11)	-1.59(-1.78to-1.41)	45.55(32.32to58.78)	-2.75(-3.02to-2.47)

Vitamin A deficiency	3944.34(3821.24to4067.44)	-3.22(-3.52to-2.92)	3.29(2.46to4.40)	-0.91(-1.02to-0.79)
Dietary iron deficiency	-	-	287.44(254.21to320.67)	-0.37(-0.46to-0.28)
Other nutritional deficiency	-	-	11.23(9.60to13.14)	3.57(1.79to5.39)
**Mozambique**				
Nutritional deficiencies	338.11(302.07to374.15)	-0.87(-0.96to-0.77)	422.87(382.57to463.18)	-0.99(-1.59to-0.38)
Protein-energy malnutrition	263.86(232.02to295.69)	-1.24(-1.69to-0.78)	117.40(96.16to138.64)	-1.96(-3.33to-0.58)
Iodine deficiency	74.26(57.37to91.15)	0.25(-0.78to1.28)	31.86(20.80to42.92)	-1.46(-2.17to-0.74)
Vitamin A deficiency	18080.33(17816.79to18343.88)	-3.91(-4.16to-3.65)	2.09(1.38to3.17)	-1.74(-1.84to-1.64)
Dietary iron deficiency	-	-	249.13(218.19to280.06)	-0.02(-0.25to0.22)
Other nutritional deficiency	-	-	22.39(13.12to31.67)	-2.53(-4.00to-1.05)
**Myanmar**				
Nutritional deficiencies	1087.23(1022.60to1151.86)	0.35(0.06to0.65)	668.31(617.64to718.98)	-1.55(-1.77to-1.33)
Protein-energy malnutrition	1038.30(975.15to1101.46)	0.81(0.49to1.13)	27.98(17.61to38.34)	-4.96(-5.06to-4.85)
Iodine deficiency	48.93(35.22to62.64)	-4.58(-4.85to-4.30)	18.40(16.68to20.30)	-17.49(-21.19to-13.61)
Vitamin A deficiency	3671.56(3552.80to3790.32)	-7.55(-8.60to-6.50)	0.24(0.10to0.57)	-2.35(-2.75to-1.95)
Dietary iron deficiency	-	-	611.43(562.97to659.90)	-0.59(-0.63to-0.55)
Other nutritional deficiency	-	-	10.25(8.99to11.69)	3.20(1.55to4.87)
**Namibia**				
Nutritional deficiencies	546.88(501.04to592.71)	0.32(-0.37to1.02)	226.69(197.18to256.20)	-4.80(-5.16to-4.44)
Protein-energy malnutrition	504.10(460.09to548.10)	0.48(-0.30to1.26)	51.71(37.62to65.81)	-7.04(-7.63to-6.44)
Iodine deficiency	42.78(29.96to55.60)	-1.19(-1.32to-1.07)	15.72(9.45to26.14)	-1.24(-1.39to-1.09)
Vitamin A deficiency	4957.99(4819.98to5095.99)	-1.99(-2.36to-1.61)	4.44(1.70to11.56)	-0.85(-0.95to-0.74)
Dietary iron deficiency	-	-	146.83(123.08to170.58)	-4.24(-4.91to-3.57)
Other nutritional deficiency	-	-	7.99(3.92to16.31)	-3.00(-3.81to-2.18)
**Nauru**				
Nutritional deficiencies	825.70(769.38to882.02)	-0.28(-0.43to-0.12)	505.28(461.22to549.34)	0.38(-0.15to0.90)
Protein-energy malnutrition	821.04(764.88to877.20)	-0.28(-0.45to-0.11)	41.37(28.76to53.98)	-1.31(-1.43to-1.19)
Iodine deficiency	4.66(0.00to6772520.28)	-0.29(-1.25to0.69)	0.83(0.00to3.13E+14)	1.13(-0.73to3.03)
Vitamin A deficiency	5727.26(5578.92to5875.59)	-0.82(-1.34to-0.30)	1.71(0.00to2.62E+10)	-0.46(-0.75to-0.16)
Dietary iron deficiency	-	-	451.91(410.25to493.58)	0.50(-0.06to1.07)
Other nutritional deficiency	-	-	9.46(0.00to200975.95)	3.11(1.18to5.08)
**Nepal**				
Nutritional deficiencies	1437.91(1363.59to1512.23)	0.60(0.46to0.74)	652.97(602.89to703.06)	-1.80(-1.97to-1.62)
Protein-energy malnutrition	1340.26(1268.50to1412.01)	0.33(0.08to0.58)	90.19(71.58to108.80)	-6.16(-6.91to-5.41)
Iodine deficiency	97.65(78.28to117.02)	4.03(2.61to5.48)	23.00(13.60to32.40)	7.24(4.34to10.23)

Vitamin A deficiency	6237.71(6082.91to6392.51)	-4.52(-4.62to-4.43)	2.37(1.66to3.38)	-0.26(-0.30to-0.22)
Dietary iron deficiency	-	-	505.83(461.75to549.91)	-0.79(-0.97to-0.61)
Other nutritional deficiency	-	-	31.58(20.57to42.59)	-3.38(-3.98to-2.77)
**Netherlands**				
Nutritional deficiencies	1009.51(947.24to1071.79)	-0.09(-0.36to0.18)	106.73(86.48to126.98)	-0.55(-0.81to-0.28)
Protein-energy malnutrition	949.06(888.67to1009.44)	-0.10(-0.38to0.19)	41.68(29.03to54.34)	-0.02(-0.27to0.23)
Iodine deficiency	60.46(45.22to75.70)	-0.03(-0.07to0.00)	13.63(10.83to17.17)	-0.02(-0.08to0.04)
Vitamin A deficiency	366.89(329.35to404.44)	-1.13(-1.22to-1.03)	0.00(0.00to0.00)	-
Dietary iron deficiency	-	-	32.06(20.96to43.16)	-3.16(-3.48to-2.85)
Other nutritional deficiency	-	-	19.35(15.94to23.48)	3.67(1.68to5.69)
**New Zealand**				
Nutritional deficiencies	565.87(519.24to612.49)	0.95(0.78to1.11)	82.09(64.33to99.85)	-0.06(-0.32to0.20)
Protein-energy malnutrition	549.04(503.11to594.97)	0.98(0.81to1.15)	19.75(13.69to28.47)	1.09(0.89to1.30)
Iodine deficiency	16.83(11.32to25.01)	-0.10(-0.10to-0.10)	3.72(1.60to8.65)	-0.21(-0.26to-0.17)
Vitamin A deficiency	655.40(605.22to705.57)	-0.31(-0.50to-0.12)	0.00(0.00to0.00)	-
Dietary iron deficiency	0.00(0.00to0.00)	-	49.33(35.57to63.10)	-1.19(-1.56to-0.82)
Other nutritional deficiency	0.00(0.00to0.00)	-	9.29(5.45to15.84)	4.12(2.24to6.03)
**Nicaragua**				
Nutritional deficiencies	332.87(297.11to368.63)	0.05(-0.14to0.24)	115.23(94.19to136.27)	-6.29(-6.79to-5.79)
Protein-energy malnutrition	306.71(272.38to341.04)	0.16(-0.05to0.38)	24.55(14.84to34.26)	-3.12(-4.18to-2.05)
Iodine deficiency	26.16(16.14to36.19)	-1.01(-1.06to-0.95)	20.36(11.52to29.21)	-8.15(-10.52to-5.71)
Vitamin A deficiency	1979.59(1892.38to2066.79)	-6.47(-8.07to-4.84)	3.04(1.51to6.12)	-0.55(-0.66to-0.45)
Dietary iron deficiency	-	-	63.12(47.55to78.69)	-7.42(-7.63to-7.22)
Other nutritional deficiency	-	-	4.15(2.28to7.56)	3.06(1.37to4.78)
**Niger**				
Nutritional deficiencies	561.52(515.08to607.97)	0.45(0.30to0.61)	404.16(364.76to443.57)	-3.29(-3.73to-2.84)
Protein-energy malnutrition	359.72(322.54to396.89)	-0.04(-0.20to0.11)	37.07(25.14to49.00)	-4.93(-5.21to-4.64)
Iodine deficiency	201.81(173.96to229.65)	1.31(0.63to1.99)	72.77(56.05to89.49)	0.56(0.09to1.04)
Vitamin A deficiency	19722.48(19447.23to19997.74)	-0.26(-0.37to-0.15)	4.30(3.03to6.10)	-1.25(-1.35to-1.15)
Dietary iron deficiency	-	-	279.97(247.18to312.77)	-4.06(-4.63to-3.49)
Other nutritional deficiency	-	-	10.05(8.00to12.63)	-3.58(-3.97to-3.20)
**Nigeria**				
Nutritional deficiencies	558.00(511.70to604.30)	0.23(-0.60to1.07)	433.13(392.34to473.92)	2.39(1.64to3.14)
Protein-energy malnutrition	460.64(418.57to502.71)	0.36(-0.62to1.35)	17.30(16.86to17.75)	-3.44(-4.06to-2.81)
Iodine deficiency	97.36(78.02to116.70)	-0.42(-0.51to-0.34)	22.97(13.57to32.36)	-3.79(-4.09to-3.49)
Vitamin A deficiency	6044.35(5891.97to6196.73)	-2.61(-4.46to-0.72)	6.15(5.69to6.64)	0.90(0.51to1.29)

Dietary iron deficiency	0.00(0.00to0.00)	-	378.83(340.69to416.98)	4.19(3.33to5.06)
Other nutritional deficiency	0.00(0.00to0.00)	-	7.88(7.68to8.09)	-1.02(-1.97to-0.07)
**Niue**				
Nutritional deficiencies	1045.17(981.81to1108.54)	0.46(0.30to0.62)	398.35(359.23to437.47)	-1.48(-1.92to-1.05)
Protein-energy malnutrition	1040.59(977.36to1103.81)	0.47(0.32to0.63)	34.13(22.68to45.58)	-0.36(-0.49to-0.22)
Iodine deficiency	4.58(0.00to5.39E+17)	-1.85(-2.23to-1.46)	0.82(0.00to2.29E+40)	-7.85(-11.48to-4.08)
Vitamin A deficiency	3496.27(3380.37to3612.16)	-3.93(-4.41to-3.45)	1.54(0.00to4.22E+29)	-0.87(-0.96to-0.77)
Dietary iron deficiency	-	-	350.12(313.44to386.79)	-1.70(-2.14to-1.25)
Other nutritional deficiency	-	-	11.75(0.00to5.41E+11)	4.09(1.85to6.38)
**North Macedonia**				
Nutritional deficiencies	854.19(796.91to911.47)	0.06(-0.03to0.14)	208.39(180.10to236.69)	-0.06(-0.46to0.34)
Protein-energy malnutrition	840.46(783.64to897.28)	0.06(-0.02to0.14)	33.04(21.78to44.31)	-0.95(-1.53to-0.35)
Iodine deficiency	13.73(7.43to25.38)	-0.91(-1.05to-0.78)	3.45(1.01to11.75)	-1.08(-1.22to-0.95)
Vitamin A deficiency	12623.04(12402.83to12843.25)	-0.33(-0.92to0.26)	0.00(0.00to0.00)	-
Dietary iron deficiency	-	-	156.35(131.84to180.85)	-0.09(-0.43to0.26)
Other nutritional deficiency	-	-	15.55(8.73to27.69)	3.74(1.51to6.02)
**Northern Mariana Islands**				
Nutritional deficiencies	1212.48(1144.23to1280.72)	1.62(1.59to1.65)	369.66(331.98to407.35)	0.85(0.57to1.13)
Protein-energy malnutrition	1208.24(1140.11to1276.37)	1.69(1.65to1.72)	41.03(28.48to53.59)	1.54(1.49to1.60)
Iodine deficiency	4.24(0.00to13627.49)	-1.59(-1.87to-1.31)	0.75(0.00to157562417.00)	-1.78(-2.14to-1.43)
Vitamin A deficiency	2874.12(2769.04to2979.20)	-3.30(-3.55to-3.04)	0.00(0.00to0.00)	-
Dietary iron deficiency	-	-	314.29(279.55to349.04)	0.46(0.21to0.72)
Other nutritional deficiency	-	-	13.58(0.15to1235.34)	4.77(2.72to6.86)
**Norway**				
Nutritional deficiencies	1392.04(1318.91to1465.16)	1.66(1.21to2.11)	136.05(113.18to158.91)	1.08(0.75to1.40)
Protein-energy malnutrition	1325.23(1253.88to1396.58)	1.76(1.28to2.25)	57.54(42.67to72.41)	1.97(1.51to2.43)
Iodine deficiency	66.81(50.79to82.83)	-0.06(-0.20to0.08)	14.62(9.93to21.52)	-0.13(-0.21to-0.05)
Vitamin A deficiency	310.12(275.61to344.64)	-1.47(-1.56to-1.39)	0.00(0.00to0.00)	-
Dietary iron deficiency	0.00(0.00to0.00)	-	37.55(25.54to49.56)	-1.43(-1.59to-1.26)
Other nutritional deficiency	0.00(0.00to0.00)	-	26.33(16.28to36.39)	5.16(3.27to7.09)
**Oman**				
Nutritional deficiencies	794.68(739.42to849.93)	0.46(0.39to0.52)	213.98(185.31to242.65)	-0.32(-1.35to0.72)
Protein-energy malnutrition	770.64(716.23to825.05)	0.52(0.46to0.57)	14.42(9.32to22.31)	2.77(2.45to3.09)
Iodine deficiency	24.04(14.43to33.65)	-1.20(-1.36to-1.04)	6.39(3.32to12.31)	-1.67(-1.76to-1.57)
Vitamin A deficiency	1206.45(1138.37to1274.53)	-6.54(-6.82to-6.26)	7.37(4.00to13.57)	0.06(-0.05to0.17)
Dietary iron deficiency	-	-	166.15(140.89to191.42)	-0.11(-1.54to1.34)

Other nutritional deficiency	-	-	19.65(13.52to28.55)	-2.43(-3.14to-1.71)
**Pakistan**				
Nutritional deficiencies	1603.01(1524.53to1681.48)	0.97(-0.11to2.06)	1186.26(1118.75to1253.77)	-0.62(-0.76to-0.49)
Protein-energy malnutrition	960.66(899.91to1021.41)	-0.68(-1.00to-0.36)	50.54(36.61to64.47)	-3.52(-4.15to-2.88)
Iodine deficiency	642.34(592.67to692.02)	4.23(1.73to6.80)	191.27(164.17to218.38)	0.50(0.07to0.93)
Vitamin A deficiency	3081.15(2972.35to3189.94)	-4.31(-4.51to-4.12)	7.34(6.78to7.96)	0.13(-0.38to0.65)
Dietary iron deficiency	0.00(0.00to0.00)	-	789.33(734.27to844.40)	-0.03(-0.26to0.19)
Other nutritional deficiency	0.00(0.00to0.00)	-	147.77(123.94to171.59)	-3.13(-3.87to-2.39)
**Palau**				
Nutritional deficiencies	933.27(873.39to993.14)	0.21(0.12to0.30)	392.39(353.57to431.22)	-0.55(-0.83to-0.27)
Protein-energy malnutrition	928.76(869.03to988.50)	0.22(0.13to0.31)	27.38(17.13to37.64)	0.08(-0.03to0.19)
Iodine deficiency	4.50(0.00to1171064.07)	-1.34(-1.65to-1.04)	0.80(0.00to5.54E+12)	-1.45(-1.73to-1.16)
Vitamin A deficiency	3477.40(3361.82to3592.98)	-2.48(-2.97to-1.98)	1.54(0.00to2776779037.00)	-0.68(-0.70to-0.66)
Dietary iron deficiency	-	-	352.14(315.36to388.92)	-0.70(-0.98to-0.42)
Other nutritional deficiency	-	-	10.53(0.00to36673.39)	3.91(1.71to6.14)
**Palestine**				
Nutritional deficiencies	833.88(777.29to890.48)	0.82(0.78to0.87)	193.26(166.01to220.51)	-1.13(-1.84to-0.42)
Protein-energy malnutrition	809.04(753.29to864.79)	0.89(0.84to0.95)	19.50(14.05to27.05)	1.45(1.28to1.62)
Iodine deficiency	24.84(15.07to34.61)	-0.94(-1.08to-0.80)	6.67(3.81to11.68)	-14.93(-19.28to-10.36)
Vitamin A deficiency	3783.56(3663.00to3904.13)	-4.45(-4.94to-3.96)	4.66(2.39to9.10)	-0.48(-0.64to-0.31)
Dietary iron deficiency	-	-	153.46(129.18to177.74)	-0.55(-1.73to0.64)
Other nutritional deficiency	-	-	8.97(5.53to14.53)	3.05(1.43to4.70)
**Panama**				
Nutritional deficiencies	707.23(655.11to759.35)	-0.23(-0.32to-0.15)	414.63(374.72to454.54)	-1.51(-1.60to-1.42)
Protein-energy malnutrition	695.43(643.75to747.12)	-0.21(-0.29to-0.13)	34.32(22.84to45.81)	-3.78(-4.15to-3.41)
Iodine deficiency	11.80(7.41to18.78)	-1.20(-1.31to-1.09)	3.31(1.37to7.96)	-10.83(-13.91to-7.64)
Vitamin A deficiency	2706.42(2604.45to2808.38)	-2.58(-2.93to-2.23)	3.28(1.36to7.92)	-0.14(-0.31to0.02)
Dietary iron deficiency	-	-	368.41(330.79to406.03)	-1.14(-1.28to-1.00)
Other nutritional deficiency	-	-	5.30(2.65to10.61)	-3.87(-5.20to-2.52)
**Papua New Guinea**				
Nutritional deficiencies	705.98(653.90to758.06)	-1.51(-1.84to-1.18)	535.92(490.54to581.29)	-0.88(-1.29to-0.47)
Protein-energy malnutrition	700.24(648.37to752.11)	-1.52(-1.86to-1.17)	31.97(20.89to43.05)	-0.90(-1.05to-0.76)
Iodine deficiency	5.74(3.74to8.81)	-1.12(-2.20to-0.02)	2.99(1.65to5.41)	-1.86(-2.60to-1.11)
Vitamin A deficiency	9011.95(8825.89to9198.02)	-0.04(-1.00to0.93)	1.95(0.94to4.07)	-0.31(-0.39to-0.23)

Dietary iron deficiency	-	-	492.11(448.63to535.59)	-0.91(-1.38to-0.44)
Other nutritional deficiency	-	-	6.90(4.67to10.19)	1.92(0.22to3.66)
**Paraguay**				
Nutritional deficiencies	808.89(753.15to864.64)	1.53(0.92to2.15)	424.27(383.90to464.64)	0.14(-0.22to0.50)
Protein-energy malnutrition	800.85(745.39to856.32)	1.56(0.94to2.19)	34.44(22.94to45.94)	1.26(0.74to1.79)
Iodine deficiency	8.04(5.27to12.28)	-0.98(-1.08to-0.89)	1.78(0.73to4.38)	-7.04(-10.50to-3.45)
Vitamin A deficiency	5515.63(5370.06to5661.19)	-1.41(-1.81to-1.00)	4.13(2.29to7.45)	-0.06(-0.33to0.21)
Dietary iron deficiency	-	-	376.77(338.72to414.81)	0.07(-0.29to0.42)
Other nutritional deficiency	-	-	7.16(4.57to11.21)	3.99(2.36to5.64)
**Peru**				
Nutritional deficiencies	447.13(405.69to488.58)	-3.06(-3.25to-2.87)	241.17(210.74to271.61)	-5.62(-6.04to-5.20)
Protein-energy malnutrition	441.74(400.55to482.94)	-3.09(-3.28to-2.90)	32.57(21.38to43.75)	-6.54(-7.89to-5.16)
Iodine deficiency	5.39(4.26to6.82)	-0.42(-0.63to-0.20)	1.09(0.65to1.84)	-0.46(-0.73to-0.19)
Vitamin A deficiency	5567.81(5421.56to5714.06)	-2.44(-2.57to-2.32)	3.63(2.72to4.83)	-0.64(-0.84to-0.44)
Dietary iron deficiency	-	-	194.16(166.85to221.47)	-5.59(-5.97to-5.21)
Other nutritional deficiency	-	-	9.72(8.16to11.59)	-4.30(-6.05to-2.51)
**Philippines**				
Nutritional deficiencies	1521.24(1444.79to1597.68)	-0.09(-0.57to0.39)	298.19(264.34to332.03)	-3.89(-4.39to-3.39)
Protein-energy malnutrition	1302.61(1231.87to1373.35)	0.32(-0.25to0.90)	35.63(23.93to47.33)	-0.14(-0.33to0.05)
Iodine deficiency	218.63(189.65to247.61)	-2.08(-2.20to-1.95)	58.18(43.23to73.14)	-5.59(-6.75to-4.42)
Vitamin A deficiency	4986.61(4848.20to5125.01)	0.54(-0.62to1.70)	2.73(2.45to3.04)	-0.37(-0.52to-0.21)
Dietary iron deficiency	0.00(0.00to0.00)	-	192.66(165.46to219.87)	-4.26(-5.22to-3.30)
Other nutritional deficiency	0.00(0.00to0.00)	-	8.98(8.60to9.38)	2.51(1.09to3.94)
**Poland**				
Nutritional deficiencies	644.30(594.55to694.06)	0.98(0.70to1.26)	192.40(165.21to219.58)	-1.00(-1.11to-0.89)
Protein-energy malnutrition	630.62(581.40to679.84)	1.02(0.74to1.31)	22.91(13.53to32.29)	0.74(0.45to1.03)
Iodine deficiency	13.68(11.79to15.88)	-0.70(-0.96to-0.45)	3.38(2.51to4.56)	-0.71(-1.03to-0.39)
Vitamin A deficiency	8696.53(8513.75to8879.31)	-2.44(-2.66to-2.22)	0.00(0.00to0.00)	-
Dietary iron deficiency	0.00(0.00to0.00)	-	155.78(131.32to180.24)	-1.44(-1.53to-1.36)
Other nutritional deficiency	0.00(0.00to0.00)	-	10.33(8.70to12.26)	3.50(1.80to5.23)
**Portugal**				
Nutritional deficiencies	1000.41(938.41to1062.40)	0.99(0.76to1.21)	124.21(102.37to146.06)	-0.12(-0.36to0.13)
Protein-energy malnutrition	912.71(853.49to971.92)	1.03(0.81to1.25)	39.94(27.55to52.32)	1.17(0.96to1.39)
Iodine deficiency	87.70(69.34to106.05)	0.53(0.24to0.82)	20.80(11.86to29.74)	0.68(0.35to1.01)
Vitamin A deficiency	782.67(727.84to837.50)	-1.98(-2.05to-1.91)	0.00(0.00to0.00)	-
Dietary iron deficiency	-	-	45.10(31.94to58.26)	-2.29(-2.73to-1.85)

Other nutritional deficiency	-	-	18.38(14.31to23.60)	4.89(2.58to7.26)
**Puerto Rico**				
Nutritional deficiencies	793.88(738.65to849.10)	1.03(0.93to1.13)	262.74(230.97to294.51)	-1.23(-1.52to-0.94)
Protein-energy malnutrition	784.56(729.66to839.46)	1.10(0.99to1.20)	29.63(18.96to40.30)	0.32(0.11to0.53)
Iodine deficiency	9.32(5.13to16.91)	-2.81(-2.95to-2.67)	4.05(1.64to10.00)	-3.01(-3.38to-2.64)
Vitamin A deficiency	1478.46(1403.09to1553.82)	-2.83(-3.18to-2.48)	0.00(0.00to0.00)	-
Dietary iron deficiency	-	-	217.76(188.84to246.69)	-1.63(-2.00to-1.26)
Other nutritional deficiency	-	-	11.29(6.57to19.41)	3.60(1.60to5.65)
**Qatar**				
Nutritional deficiencies	1054.36(990.72to1118.00)	3.92(3.48to4.36)	205.35(177.26to233.43)	-3.15(-3.67to-2.62)
Protein-energy malnutrition	1033.02(970.02to1096.02)	4.06(3.62to4.51)	26.20(16.17to36.23)	3.69(3.40to3.99)
Iodine deficiency	21.34(12.29to30.39)	-1.14(-1.20to-1.07)	5.49(1.96to15.38)	-1.52(-1.58to-1.46)
Vitamin A deficiency	808.29(752.57to864.02)	-4.42(-4.60to-4.24)	0.00(0.00to0.00)	-
Dietary iron deficiency	-	-	161.55(136.64to186.46)	-4.27(-4.80to-3.73)
Other nutritional deficiency	-	-	12.11(6.05to24.23)	6.42(4.56to8.31)
**Republic of Korea**				
Nutritional deficiencies	483.33(440.24to526.42)	-5.81(-6.15to-5.47)	250.28(219.27to281.28)	-4.77(-5.17to-4.37)
Protein-energy malnutrition	466.74(424.39to509.08)	-5.96(-6.31to-5.61)	11.30(9.82to12.99)	-6.40(-6.75to-6.05)
Iodine deficiency	16.59(14.78to18.62)	-1.11(-1.15to-1.08)	3.74(2.93to4.77)	-1.42(-1.54to-1.29)
Vitamin A deficiency	702.34(650.40to754.29)	-2.99(-3.19to-2.78)	0.00(0.00to0.00)	-
Dietary iron deficiency	-	-	229.73(200.03to259.44)	-4.76(-5.18to-4.33)
Other nutritional deficiency	-	-	5.50(4.50to6.72)	-3.87(-5.34to-2.37)
**Republic of Moldova**				
Nutritional deficiencies	340.09(303.94to376.23)	1.71(1.53to1.89)	318.35(283.38to353.32)	-1.28(-1.45to-1.11)
Protein-energy malnutrition	334.54(298.69to370.39)	1.75(1.55to1.96)	14.96(11.71to19.12)	1.03(0.36to1.71)
Iodine deficiency	5.55(3.99to7.72)	-0.02(-0.79to0.76)	3.22(1.23to8.44)	-0.62(-1.18to-0.06)
Vitamin A deficiency	2584.21(2484.58to2683.85)	-3.61(-4.15to-3.06)	0.00(0.00to0.00)	-
Dietary iron deficiency	-	-	293.00(259.45to326.55)	-1.42(-1.59to-1.25)
Other nutritional deficiency	-	-	7.17(3.76to13.68)	3.57(1.86to5.31)
**Romania**				
Nutritional deficiencies	519.99(475.30to564.69)	0.84(0.41to1.27)	184.08(157.49to210.67)	-1.94(-2.21to-1.66)
Protein-energy malnutrition	501.78(457.88to545.69)	1.00(0.58to1.41)	16.08(13.15to19.65)	1.15(0.69to1.62)
Iodine deficiency	18.21(15.08to21.99)	-2.43(-3.02to-1.84)	5.07(3.55to7.25)	-2.51(-2.98to-2.03)
Vitamin A deficiency	10360.56(10161.06to10560.06)	-2.83(-2.95to-2.71)	0.00(0.00to0.00)	-
Dietary iron deficiency	-	-	156.36(131.85to180.86)	-2.32(-2.59to-2.05)
Other nutritional deficiency	-	-	6.57(4.80to9.00)	3.41(2.07to4.78)

**Russian Federation**				
Nutritional deficiencies	420.69(380.49to460.89)	0.28(0.26to0.29)	177.97(151.82to204.12)	-2.44(-3.03to-1.85)
Protein-energy malnutrition	410.10(370.41to449.79)	0.29(0.27to0.30)	20.56(11.67to29.44)	-0.23(-0.53to0.08)
Iodine deficiency	10.59(9.71to11.55)	-0.07(-0.33to0.19)	7.26(6.54to8.06)	-1.30(-2.04to-0.56)
Vitamin A deficiency	223.31(194.02to252.59)	-2.99(-3.28to-2.69)	0.00(0.00to0.00)	-
Dietary iron deficiency	0.00(0.00to0.00)	-	139.79(116.62to162.97)	-2.85(-3.45to-2.25)
Other nutritional deficiency	0.00(0.00to0.00)	-	10.36(9.49to11.31)	1.46(-0.12to3.07)
**Rwanda**				
Nutritional deficiencies	706.05(653.97to758.13)	-1.05(-1.18to-0.93)	294.97(261.31to328.63)	-7.00(-7.21to-6.79)
Protein-energy malnutrition	509.95(465.68to554.21)	-1.95(-2.20to-1.71)	96.97(77.67to116.27)	-12.32(-12.75to-11.89)
Iodine deficiency	196.11(168.66to223.56)	1.67(0.77to2.58)	52.88(38.63to67.14)	-2.33(-3.18to-1.46)
Vitamin A deficiency	11697.40(11485.42to11909.38)	-3.88(-4.03to-3.73)	2.57(1.48to4.46)	-0.95(-1.03to-0.87)
Dietary iron deficiency	-	-	124.52(102.65to146.39)	-0.79(-2.09to0.53)
Other nutritional deficiency	-	-	18.03(14.63to22.21)	-9.22(-9.94to-8.50)
**Saint Kitts and Nevis**				
Nutritional deficiencies	775.26(720.68to829.83)	-0.87(-0.93to-0.81)	411.85(372.07to451.63)	-1.74(-1.86to-1.63)
Protein-energy malnutrition	764.00(709.83to818.18)	-0.82(-0.88to-0.75)	48.53(34.87to62.18)	-5.05(-5.44to-4.66)
Iodine deficiency	11.25(0.23to560.99)	-3.10(-3.19to-3.02)	5.16(0.02to1656.85)	-3.31(-3.39to-3.23)
Vitamin A deficiency	2181.68(2090.14to2273.23)	-3.05(-3.27to-2.82)	2.91(0.00to6324.27)	0.42(0.20to0.64)
Dietary iron deficiency	-	-	340.71(304.53to376.89)	-1.10(-1.12to-1.08)
Other nutritional deficiency	-	-	14.54(0.47to452.89)	-1.04(-2.57to0.52)
**Saint Lucia**				
Nutritional deficiencies	610.36(561.94to658.79)	-1.03(-1.30to-0.76)	511.14(466.83to555.46)	-0.31(-0.38to-0.23)
Protein-energy malnutrition	601.53(553.46to649.61)	-1.02(-1.30to-0.75)	40.51(28.04to52.98)	-1.87(-2.09to-1.66)
Iodine deficiency	8.83(0.69to113.18)	-1.62(-1.79to-1.45)	4.09(0.10to173.67)	-1.94(-2.09to-1.80)
Vitamin A deficiency	3363.75(3250.08to3477.43)	-2.22(-2.40to-2.04)	2.81(0.03to258.89)	0.43(0.35to0.51)
Dietary iron deficiency	-	-	446.17(404.77to487.57)	-0.19(-0.25to-0.13)
Other nutritional deficiency	-	-	17.56(2.88to107.18)	1.18(-0.43to2.82)
**Saint Vincent and the Grenadines**				
Nutritional deficiencies	628.26(579.13to677.38)	-0.39(-0.54to-0.24)	547.74(501.87to593.62)	-1.26(-1.33to-1.19)
Protein-energy malnutrition	611.73(563.25to660.21)	-0.26(-0.42to-0.09)	61.43(46.06to76.79)	-3.38(-4.00to-2.76)
Iodine deficiency	16.52(1.50to181.62)	-3.46(-3.74to-3.18)	8.11(0.27to248.23)	-3.87(-4.01to-3.73)
Vitamin A deficiency	4219.63(4092.31to4346.95)	-3.28(-3.75to-2.82)	2.89(0.01to891.39)	0.20(0.02to0.37)
Dietary iron deficiency	-	-	462.41(420.26to504.56)	-0.93(-0.99to-0.86)
Other nutritional deficiency	-	-	12.90(0.86to194.45)	1.25(-0.36to2.89)

**Samoa**				
Nutritional deficiencies	868.06(810.31to925.81)	0.11(0.04to0.17)	314.82(280.04to349.60)	0.40(0.18to0.63)
Protein-energy malnutrition	864.33(806.71to921.95)	0.11(0.05to0.18)	62.28(46.81to77.75)	-0.16(-0.23to-0.09)
Iodine deficiency	3.73(0.09to155.06)	-1.18(-1.24to-1.11)	0.66(0.00to4688.96)	-1.31(-1.43to-1.19)
Vitamin A deficiency	8917.17(8732.08to9102.25)	-1.58(-2.01to-1.15)	1.72(0.01to417.16)	-0.91(-0.94to-0.87)
Dietary iron deficiency	-	-	228.32(198.70to257.93)	0.30(0.13to0.46)
Other nutritional deficiency	-	-	21.85(12.69to31.01)	3.69(1.45to5.99)
**San Marino**				
Nutritional deficiencies	918.19(858.80to977.58)	0.80(0.73to0.88)	98.35(78.91to117.79)	0.46(0.17to0.76)
Protein-energy malnutrition	855.80(798.46to913.13)	0.86(0.78to0.93)	37.39(25.41to49.38)	1.03(0.96to1.10)
Iodine deficiency	62.39(46.91to77.87)	0.03(-0.06to0.11)	14.15(0.10to1911.87)	0.03(-0.02to0.08)
Vitamin A deficiency	355.57(318.62to392.53)	-0.99(-1.07to-0.90)	0.00(0.00to0.00)	-
Dietary iron deficiency	-	-	29.54(18.89to40.19)	-1.81(-2.07to-1.54)
Other nutritional deficiency	-	-	17.26(0.20to1466.52)	4.51(2.32to6.74)
**Sao Tome and Principe**				
Nutritional deficiencies	454.87(413.07to496.67)	-1.43(-1.83to-1.03)	441.69(400.49to482.88)	3.23(2.34to4.12)
Protein-energy malnutrition	389.86(351.16to428.56)	-1.50(-2.01to-1.00)	36.34(24.52to48.15)	-4.36(-5.92to-2.76)
Iodine deficiency	65.01(49.21to80.81)	-1.03(-1.22to-0.84)	24.70(14.96to34.44)	-1.05(-1.27to-0.83)
Vitamin A deficiency	5510.99(5365.49to5656.49)	-3.97(-4.34to-3.61)	4.18(0.14to128.70)	-1.30(-1.53to-1.08)
Dietary iron deficiency	-	-	364.59(327.17to402.02)	5.81(4.45to7.18)
Other nutritional deficiency	-	-	11.87(1.55to90.74)	-3.39(-4.61to-2.17)
**Saudi Arabia**				
Nutritional deficiencies	814.08(758.16to870.01)	0.39(0.11to0.68)	90.58(71.93to109.24)	1.06(0.14to1.99)
Protein-energy malnutrition	803.57(748.01to859.14)	0.42(0.13to0.71)	25.67(15.74to35.60)	-0.39(-0.69to-0.09)
Iodine deficiency	10.51(8.95to12.35)	-1.24(-1.36to-1.12)	2.31(1.64to3.26)	-2.06(-2.21to-1.91)
Vitamin A deficiency	208.68(180.37to237.00)	-3.92(-5.53to-2.29)	0.00(0.00to0.00)	-
Dietary iron deficiency	-	-	52.65(38.42to66.87)	1.46(0.30to2.64)
Other nutritional deficiency	-	-	9.96(8.44to11.75)	3.53(1.37to5.73)
**Senegal**				
Nutritional deficiencies	531.17(486.00to576.35)	-0.90(-1.00to-0.79)	620.00(571.19to668.80)	-0.65(-0.94to-0.35)
Protein-energy malnutrition	413.54(373.69to453.40)	-0.12(-0.47to0.23)	24.58(14.86to34.30)	-3.47(-4.61to-2.32)
Iodine deficiency	117.63(96.37to138.89)	-3.10(-3.74to-2.46)	41.50(28.87to54.13)	-3.36(-4.13to-2.57)
Vitamin A deficiency	6400.85(6244.04to6557.66)	-1.64(-2.09to-1.19)	4.30(2.87to6.43)	-1.21(-1.42to-1.01)
Dietary iron deficiency	-	-	542.23(496.59to587.87)	-0.11(-0.53to0.32)
Other nutritional deficiency	-	-	7.39(5.44to10.05)	-1.96(-2.83to-1.08)
**Serbia**				

Nutritional deficiencies	538.14(492.67to583.61)	1.32(0.99to1.65)	180.72(154.37to207.07)	-1.63(-2.04to-1.22)
Protein-energy malnutrition	529.59(484.48to574.69)	1.41(1.08to1.75)	15.29(11.32to20.64)	1.45(1.09to1.80)
Iodine deficiency	8.55(5.73to12.78)	-2.88(-3.06to-2.70)	1.93(0.83to4.50)	-3.11(-3.36to-2.85)
Vitamin A deficiency	14188.64(13955.17to14422.11)	-2.90(-3.06to-2.73)	0.00(0.00to0.00)	-
Dietary iron deficiency	-	-	156.22(131.72to180.72)	-1.98(-2.45to-1.51)
Other nutritional deficiency	-	-	7.28(4.71to11.25)	2.78(1.28to4.31)
**Seychelles**				
Nutritional deficiencies	1474.14(1398.89to1549.40)	0.41(0.30to0.52)	296.94(263.17to330.72)	-2.58(-2.63to-2.53)
Protein-energy malnutrition	1437.18(1362.87to1511.48)	0.49(0.38to0.60)	37.38(25.40to49.37)	-0.29(-0.38to-0.19)
Iodine deficiency	36.97(25.05to48.88)	-1.77(-1.97to-1.56)	6.58(0.11to396.68)	-9.70(-12.24to-7.09)
Vitamin A deficiency	1318.15(1246.99to1389.31)	-3.44(-4.91to-1.95)	1.17(0.00to19845.34)	-0.39(-0.53to-0.25)
Dietary iron deficiency	-	-	236.70(206.55to266.86)	-2.84(-2.95to-2.73)
Other nutritional deficiency	-	-	15.11(1.01to226.04)	3.60(1.48to5.76)
**Sierra Leone**				
Nutritional deficiencies	513.83(469.40to558.26)	-0.94(-1.18to-0.70)	356.08(319.10to393.07)	-1.53(-1.83to-1.24)
Protein-energy malnutrition	414.50(374.59to454.40)	-0.47(-0.74to-0.19)	60.63(45.37to75.90)	-1.75(-2.79to-0.70)
Iodine deficiency	99.33(79.80to118.87)	-2.44(-2.64to-2.24)	39.53(27.20to51.85)	-2.00(-2.36to-1.63)
Vitamin A deficiency	9429.17(9238.85to9619.50)	-2.06(-2.54to-1.59)	3.95(2.27to6.87)	-0.22(-0.74to0.30)
Dietary iron deficiency	-	-	233.93(203.96to263.91)	-1.44(-1.55to-1.34)
Other nutritional deficiency	-	-	18.04(13.92to23.38)	-0.82(-2.04to0.42)
**Singapore**				
Nutritional deficiencies	414.83(374.91to454.75)	0.35(0.32to0.38)	184.62(157.99to211.26)	-2.76(-2.81to-2.71)
Protein-energy malnutrition	398.32(359.20to437.43)	0.39(0.36to0.43)	7.68(4.71to12.54)	0.53(0.45to0.61)
Iodine deficiency	16.51(11.82to23.06)	-0.59(-0.67to-0.51)	3.74(1.86to7.55)	-0.71(-0.74to-0.68)
Vitamin A deficiency	752.86(699.08to806.63)	-3.34(-3.51to-3.17)	0.00(0.00to0.00)	-
Dietary iron deficiency	-	-	169.50(143.98to195.01)	-3.00(-3.04to-2.95)
Other nutritional deficiency	-	-	3.70(1.83to7.49)	3.50(1.55to5.48)
**Slovakia**				
Nutritional deficiencies	519.79(475.11to564.48)	0.38(-0.03to0.79)	148.76(124.85to172.66)	-1.25(-1.30to-1.19)
Protein-energy malnutrition	507.00(462.87to551.13)	0.44(0.03to0.85)	17.45(12.31to24.74)	0.51(0.15to0.87)
Iodine deficiency	12.80(8.51to19.23)	-1.38(-1.67to-1.08)	3.16(1.39to7.18)	-1.70(-2.04to-1.36)
Vitamin A deficiency	8695.97(8513.19to8878.74)	-2.35(-2.51to-2.20)	0.00(0.00to0.00)	-
Dietary iron deficiency	-	-	119.93(98.46to141.39)	-1.64(-1.72to-1.57)
Other nutritional deficiency	-	-	8.21(4.94to13.66)	3.55(1.73to5.41)
**Slovenia**				
Nutritional deficiencies	515.07(470.58to559.55)	0.13(-0.31to0.57)	119.55(98.12to140.98)	-1.27(-1.65to-0.88)

Protein-energy malnutrition	502.34(458.41to546.27)	0.18(-0.27to0.62)	17.34(9.46to31.79)	0.27(-0.12to0.67)
Iodine deficiency	12.73(6.27to25.83)	-1.29(-1.61to-0.98)	3.14(0.76to13.05)	-1.45(-1.85to-1.05)
Vitamin A deficiency	7305.03(7137.51to7472.55)	-2.07(-2.25to-1.89)	0.00(0.00to0.00)	-
Dietary iron deficiency	-	-	90.86(72.18to109.54)	-1.76(-2.25to-1.27)
Other nutritional deficiency	-	-	8.21(3.40to19.82)	3.19(1.39to5.02)
**Solomon Islands**				
Nutritional deficiencies	738.74(685.47to792.01)	-0.93(-1.22to-0.63)	557.14(510.87to603.40)	0.24(-0.26to0.74)
Protein-energy malnutrition	733.30(680.22to786.38)	-0.93(-1.23to-0.63)	83.59(65.67to101.51)	-1.34(-1.64to-1.04)
Iodine deficiency	5.44(0.98to30.24)	-0.25(-0.59to0.09)	3.31(0.37to29.84)	-0.01(-0.48to0.46)
Vitamin A deficiency	15263.77(15021.62to15505.92)	-1.39(-1.76to-1.02)	1.87(0.10to34.87)	-0.32(-0.57to-0.07)
Dietary iron deficiency	-	-	461.44(419.34to503.55)	0.60(0.04to1.17)
Other nutritional deficiency	-	-	6.92(1.51to31.66)	2.39(0.60to4.22)
**Somalia**				
Nutritional deficiencies	1631.92(1552.75to1711.10)	-1.70(-1.93to-1.48)	1330.95(1259.44to1402.45)	-2.17(-2.25to-2.09)
Protein-energy malnutrition	454.20(412.43to495.98)	-5.15(-5.78to-4.51)	322.02(286.85to357.19)	-3.81(-4.00to-3.62)
Iodine deficiency	1177.72(1110.46to1244.98)	0.00(-0.11to0.11)	402.48(363.16to441.80)	0.07(-0.05to0.19)
Vitamin A deficiency	51411.19(50966.78to51855.60)	-0.47(-0.56to-0.38)	3.49(2.33to5.22)	-0.39(-0.55to-0.23)
Dietary iron deficiency	-	-	566.82(520.15to613.48)	-2.10(-2.48to-1.73)
Other nutritional deficiency	-	-	36.14(24.36to47.93)	-3.88(-4.20to-3.56)
**South Africa**				
Nutritional deficiencies	339.19(303.10to375.29)	-2.40(-2.94to-1.86)	378.74(340.60to416.89)	-2.64(-3.80to-1.45)
Protein-energy malnutrition	295.85(262.14to329.56)	-2.61(-3.24to-1.98)	33.30(21.99to44.61)	-7.65(-11.56to-3.57)
Iodine deficiency	43.34(30.44to56.25)	-0.83(-0.95to-0.72)	9.67(8.46to11.07)	-3.63(-4.78to-2.47)
Vitamin A deficiency	2687.58(2585.97to2789.19)	-2.76(-3.34to-2.17)	4.27(3.48to5.22)	-0.20(-0.65to0.26)
Dietary iron deficiency	0.00(0.00to0.00)	-	325.11(289.77to360.45)	-0.85(-1.11to-0.59)
Other nutritional deficiency	0.00(0.00to0.00)	-	6.40(5.42to7.55)	-6.40(-8.21to-4.56)
**South Sudan**				
Nutritional deficiencies	643.41(593.70to693.13)	-1.93(-2.35to-1.50)	465.64(423.35to507.94)	-4.12(-4.57to-3.67)
Protein-energy malnutrition	525.92(480.97to570.87)	-2.43(-2.93to-1.92)	144.97(121.37to168.57)	-6.27(-7.27to-5.25)
Iodine deficiency	117.49(96.25to138.74)	0.95(0.92to0.98)	37.26(25.29to49.22)	0.81(0.76to0.87)
Vitamin A deficiency	15773.77(15527.60to16019.93)	-2.43(-2.50to-2.36)	5.44(3.41to8.67)	-0.26(-0.43to-0.10)
Dietary iron deficiency	-	-	256.67(225.27to288.07)	-1.82(-2.05to-1.59)
Other nutritional deficiency	-	-	21.31(12.26to30.36)	-6.52(-7.41to-5.62)
**Spain**				
Nutritional deficiencies	1107.77(1042.54to1173.01)	1.40(1.01to1.79)	127.16(105.06to149.26)	-1.25(-1.67to-0.83)
Protein-energy	1043.77(980.44to1107.09)	1.50(1.08to1.92)	39.48(27.16to51.79)	1.46(1.08to1.84)

malnutrition				
Iodine deficiency	64.01(48.33to79.69)	-0.11(-0.16to-0.05)	14.54(12.68to16.66)	-0.13(-0.15to-0.10)
Vitamin A deficiency	655.10(604.93to705.27)	-2.02(-2.12to-1.92)	0.00(0.00to0.00)	-
Dietary iron deficiency	-	-	55.11(40.56to69.67)	-3.95(-4.48to-3.42)
Other nutritional deficiency	-	-	18.03(15.95to20.37)	4.93(3.00to6.89)
**Sri Lanka**				
Nutritional deficiencies	1266.62(1196.87to1336.38)	0.01(-0.08to0.10)	463.31(421.12to505.50)	-3.56(-3.76to-3.36)
Protein-energy malnutrition	1165.35(1098.44to1232.26)	0.31(0.20to0.41)	36.21(24.41to48.00)	-2.32(-2.72to-1.91)
Iodine deficiency	101.28(81.55to121.00)	-2.17(-2.21to-2.12)	20.30(11.47to29.13)	-7.70(-9.12to-6.26)
Vitamin A deficiency	4011.10(3886.97to4135.24)	-5.68(-6.32to-5.04)	1.24(0.66to2.31)	-0.67(-0.83to-0.51)
Dietary iron deficiency	-	-	389.22(350.56to427.89)	-3.54(-3.67to-3.41)
Other nutritional deficiency	-	-	16.34(13.77to19.39)	3.06(0.93to5.23)
**Sudan**				
Nutritional deficiencies	842.78(785.88to899.68)	0.86(-0.69to2.44)	382.50(344.17to420.83)	-1.35(-1.38to-1.33)
Protein-energy malnutrition	793.89(738.67to849.12)	2.05(-0.04to4.18)	35.81(24.08to47.54)	-4.72(-5.06to-4.38)
Iodine deficiency	48.89(35.19to62.59)	-9.69(-12.33to-6.96)	55.65(41.02to70.27)	-4.05(-4.55to-3.54)
Vitamin A deficiency	5720.76(5572.52to5869.01)	-4.66(-5.00to-4.32)	4.20(3.30to5.35)	-0.59(-0.82to-0.36)
Dietary iron deficiency	-	-	275.34(242.81to307.86)	0.11(0.02to0.21)
Other nutritional deficiency	-	-	11.50(9.94to13.32)	2.66(0.36to5.00)
**Suriname**				
Nutritional deficiencies	555.29(509.11to601.48)	0.19(-0.39to0.76)	447.39(405.94to488.85)	-0.77(-1.12to-0.43)
Protein-energy malnutrition	540.25(494.69to585.80)	0.36(-0.23to0.96)	33.06(21.79to44.33)	-1.67(-2.43to-0.90)
Iodine deficiency	15.05(4.99to45.40)	-3.54(-3.93to-3.15)	7.16(1.44to35.50)	-4.21(-4.57to-3.85)
Vitamin A deficiency	4361.32(4231.88to4490.76)	-3.60(-4.33to-2.86)	4.35(0.56to33.93)	0.09(0.00to0.18)
Dietary iron deficiency	-	-	394.61(355.67to433.54)	-0.65(-1.05to-0.26)
Other nutritional deficiency	-	-	8.21(1.84to36.62)	1.79(0.44to3.16)
**Sweden**				
Nutritional deficiencies	1213.49(1145.22to1281.77)	0.73(0.70to0.76)	122.01(100.36to143.66)	0.43(0.16to0.70)
Protein-energy malnutrition	1143.78(1077.49to1210.07)	0.80(0.76to0.83)	50.40(36.49to64.32)	0.94(0.87to1.01)
Iodine deficiency	69.71(53.35to86.08)	-0.23(-0.31to-0.16)	15.37(11.57to20.41)	-0.27(-0.34to-0.21)
Vitamin A deficiency	379.30(341.12to417.47)	-1.38(-1.42to-1.34)	0.00(0.00to0.00)	-
Dietary iron deficiency	0.00(0.00to0.00)	-	33.19(21.90to44.48)	-2.00(-2.78to-1.21)
Other nutritional deficiency	0.00(0.00to0.00)	-	23.05(13.64to32.46)	4.36(2.20to6.57)
**Switzerland**				
Nutritional deficiencies	980.01(918.65to1041.37)	0.71(0.56to0.87)	100.68(81.01to120.34)	0.17(-0.11to0.45)
Protein-energy	933.21(873.34to993.09)	0.75(0.59to0.92)	41.23(28.64to53.81)	0.86(0.73to1.00)

malnutrition				
Iodine deficiency	46.80(33.39to60.21)	-0.06(-0.15to0.03)	10.12(6.99to14.66)	0.23(0.05to0.42)
Vitamin A deficiency	272.97(240.58to305.35)	-0.96(-1.16to-0.77)	0.00(0.00to0.00)	
Dietary iron deficiency	-	-	30.33(19.53to41.12)	-2.40(-2.50to-2.31)
Other nutritional deficiency	-	-	19.00(14.50to24.90)	4.05(2.09to6.04)
**Syrian Arab Republic**				
Nutritional deficiencies	775.56(720.98to830.15)	0.40(-0.32to1.12)	323.80(288.53to359.07)	-2.35(-3.48to-1.20)
Protein-energy malnutrition	726.75(673.91to779.59)	0.58(-0.18to1.33)	30.42(19.61to41.23)	-0.72(-1.33to-0.11)
Iodine deficiency	48.81(35.12to62.51)	-1.99(-2.29to-1.68)	45.84(32.57to59.11)	-3.66(-3.86to-3.46)
Vitamin A deficiency	2977.67(2870.72to3084.62)	-4.60(-5.02to-4.18)	0.00(0.00to0.00)	-
Dietary iron deficiency	-	-	231.15(201.35to260.95)	-2.52(-4.33to-0.67)
Other nutritional deficiency	-	-	16.39(13.40to20.04)	0.34(-1.13to1.84)
**Taiwan**				
Nutritional deficiencies	1056.82(993.10to1120.54)	0.83(0.33to1.34)	213.21(184.59to241.83)	-0.85(-1.18to-0.52)
Protein-energy malnutrition	1047.16(983.74to1110.59)	0.87(0.36to1.38)	44.50(31.42to57.57)	0.62(0.04to1.19)
Iodine deficiency	9.65(7.74to12.04)	-1.90(-2.00to-1.79)	1.64(0.96to2.81)	-1.89(-2.01to-1.77)
Vitamin A deficiency	691.09(639.56to742.61)	-4.82(-5.31to-4.33)	0.00(0.00to0.00)	-
Dietary iron deficiency	-	-	146.64(122.91to170.38)	-1.67(-2.15to-1.19)
Other nutritional deficiency	-	-	20.43(11.57to29.29)	4.36(2.44to6.32)
**Tajikistan**				
Nutritional deficiencies	331.39(295.71to367.07)	0.54(-0.06to1.14)	439.93(398.82to481.04)	-2.38(-2.47to-2.28)
Protein-energy malnutrition	300.17(266.21to334.13)	1.21(0.52to1.92)	10.55(7.68to14.50)	-4.13(-4.74to-3.51)
Iodine deficiency	31.23(20.27to42.18)	-3.00(-3.19to-2.81)	31.68(20.65to42.71)	-5.40(-5.76to-5.05)
Vitamin A deficiency	6052.25(5899.77to6204.73)	-3.11(-4.28to-1.93)	3.91(2.32to6.59)	-0.70(-0.97to-0.43)
Dietary iron deficiency	-	-	387.54(348.95to426.12)	-1.34(-1.38to-1.30)
Other nutritional deficiency	-	-	6.25(4.14to9.45)	-9.66(-10.79to-8.52)
**Thailand**				
Nutritional deficiencies	884.60(826.31to942.90)	0.84(0.40to1.28)	130.51(108.12to152.90)	-3.62(-4.02to-3.22)
Protein-energy malnutrition	831.92(775.39to888.45)	0.96(0.58to1.34)	18.08(16.55to19.75)	-2.68(-3.03to-2.33)
Iodine deficiency	52.68(38.45to66.91)	-0.43(-1.57to0.72)	18.42(16.88to20.11)	-7.01(-8.55to-5.44)
Vitamin A deficiency	1304.00(1233.22to1374.77)	-4.44(-5.19to-3.69)	2.22(1.93to2.55)	0.32(0.12to0.51)
Dietary iron deficiency	-	-	83.95(65.99to101.91)	-3.77(-4.08to-3.47)
Other nutritional deficiency	-	-	7.84(7.12to8.62)	3.08(0.98to5.21)
**Timor Leste**				
Nutritional deficiencies	1032.52(969.54to1095.50)	-0.49(-0.57to-0.42)	294.30(260.68to327.92)	-6.81(-7.52to-6.08)
Protein-energy malnutrition	988.66(927.03to1050.29)	-0.28(-0.41to-0.15)	53.12(38.84to67.41)	-12.16(-13.25to-11.06)

Iodine deficiency	43.85(30.87to56.83)	-4.00(-5.01to-2.97)	16.35(8.15to32.78)	-16.27(-19.24to-13.19)
Vitamin A deficiency	4416.64(4286.39to4546.90)	-10.51(-11.11to-9.91)	1.57(0.17to14.83)	-1.44(-1.52to-1.36)
Dietary iron deficiency	-	-	211.14(182.66to239.62)	-3.79(-4.08to-3.50)
Other nutritional deficiency	-	-	12.12(5.40to27.19)	-1.40(-2.59to-0.18)
**Togo**				
Nutritional deficiencies	608.47(560.12to656.82)	-1.05(-1.30to-0.80)	433.07(392.28to473.86)	-1.67(-3.24to-0.07)
Protein-energy malnutrition	461.66(419.55to503.77)	-0.26(-0.34to-0.19)	52.70(38.47to66.93)	-3.87(-4.24to-3.50)
Iodine deficiency	146.81(123.06to170.56)	-3.00(-4.00to-1.98)	53.27(38.97to67.58)	-3.60(-4.70to-2.49)
Vitamin A deficiency	7271.48(7104.35to7438.62)	-0.92(-1.47to-0.37)	4.42(2.58to7.58)	-0.60(-0.74to-0.46)
Dietary iron deficiency	-	-	307.91(273.52to342.31)	-0.42(-2.66to1.88)
Other nutritional deficiency	-	-	14.76(10.99to19.81)	-2.43(-2.97to-1.88)
**Tokelau**				
Nutritional deficiencies	919.21(859.79to978.64)	-0.05(-0.08to-0.01)	469.25(426.79to511.71)	-1.20(-1.42to-0.98)
Protein-energy malnutrition	914.30(855.03to973.57)	-0.02(-0.06to0.01)	38.21(26.09to50.32)	-2.07(-2.23to-1.92)
Iodine deficiency	4.91(0.00to4.71E+18)	-2.72(-3.12to-2.32)	0.88(0.00to2.77E+42)	-3.20(-3.53to-2.86)
Vitamin A deficiency	5369.05(5225.43to5512.66)	-3.95(-4.37to-3.53)	1.69(0.00to7.56E+30)	-1.28(-1.34to-1.22)
Dietary iron deficiency	-	-	418.07(378.00to458.15)	-1.19(-1.43to-0.96)
Other nutritional deficiency	-	-	10.40(0.00to2.37E+13)	3.48(1.35to5.65)
**Tonga**				
Nutritional deficiencies	894.47(835.85to953.09)	-0.25(-0.28to-0.22)	522.70(477.89to567.51)	0.50(0.15to0.85)
Protein-energy malnutrition	889.28(830.83to947.72)	-0.24(-0.27to-0.21)	36.32(24.51to48.13)	-1.12(-1.44to-0.81)
Iodine deficiency	5.20(0.06to464.37)	-1.99(-2.48to-1.50)	0.94(0.00to36593.91)	-2.25(-2.70to-1.80)
Vitamin A deficiency	6403.77(6246.92to6560.61)	-1.80(-2.32to-1.28)	1.28(0.00to10879.99)	-1.19(-1.36to-1.01)
Dietary iron deficiency	-	-	472.02(429.44to514.60)	0.58(0.20to0.96)
Other nutritional deficiency	-	-	12.14(0.64to229.45)	3.57(1.41to5.77)
**Trinidad and Tobago**				
Nutritional deficiencies	587.69(540.18to635.21)	-1.40(-1.52to-1.28)	475.18(432.46to517.91)	-2.09(-2.16to-2.01)
Protein-energy malnutrition	567.21(520.53to613.89)	-1.16(-1.31to-1.02)	26.37(16.30to36.43)	-3.62(-3.94to-3.30)
Iodine deficiency	20.48(11.61to29.35)	-5.97(-6.25to-5.69)	9.01(3.54to22.93)	-7.01(-7.46to-6.55)
Vitamin A deficiency	2223.25(2130.83to2315.66)	-4.21(-4.55to-3.86)	2.69(0.49to14.87)	0.29(0.19to0.39)
Dietary iron deficiency	-	-	428.11(387.55to468.66)	-1.90(-1.97to-1.82)
Other nutritional deficiency	-	-	9.01(3.54to22.93)	0.68(-1.17to2.56)
**Tunisia**				
Nutritional deficiencies	759.72(705.70to813.75)	0.98(0.79to1.17)	161.15(136.27to186.03)	-0.89(-1.04to-0.73)
Protein-energy	741.57(688.19to794.94)	1.06(0.85to1.27)	20.14(11.35to28.94)	0.70(0.47to0.92)

malnutrition				
Iodine deficiency	18.16(14.57to22.61)	-1.41(-1.88to-0.94)	9.31(6.85to12.65)	-2.61(-2.77to-2.45)
Vitamin A deficiency	1567.39(1489.79to1644.98)	-5.55(-6.02to-5.09)	3.05(1.79to5.22)	0.03(-0.14to0.21)
Dietary iron deficiency	-	-	119.43(98.01to140.85)	-1.22(-1.41to-1.02)
Other nutritional deficiency	-	-	9.21(6.77to12.54)	4.11(2.29to5.96)
**Turkey**				
Nutritional deficiencies	920.69(861.21to980.16)	5.10(4.08to6.14)	301.78(267.74to335.83)	-3.33(-3.62to-3.04)
Protein-energy malnutrition	876.16(818.15to934.18)	5.85(4.74to6.96)	18.48(17.05to20.03)	0.45(-0.78to1.69)
Iodine deficiency	44.52(31.44to57.60)	-1.84(-2.03to-1.65)	30.24(19.46to41.02)	-3.89(-4.13to-3.66)
Vitamin A deficiency	3999.33(3875.38to4123.28)	-5.65(-5.75to-5.56)	0.00(0.00to0.00)	-
Dietary iron deficiency	-	-	244.97(214.29to275.64)	-3.56(-3.86to-3.26)
Other nutritional deficiency	-	-	8.10(7.17to9.15)	7.89(6.14to9.68)
**Turkmenistan**				
Nutritional deficiencies	308.40(273.98to342.82)	0.79(0.62to0.97)	408.43(368.82to448.04)	-0.81(-1.82to0.22)
Protein-energy malnutrition	287.45(254.22to320.68)	0.94(0.74to1.14)	10.89(6.99to16.97)	0.63(-0.42to1.69)
Iodine deficiency	20.95(11.98to29.92)	-0.76(-0.86to-0.67)	7.79(4.61to13.16)	-1.25(-1.33to-1.17)
Vitamin A deficiency	1922.19(1836.25to2008.12)	-2.86(-3.73to-1.99)	4.41(2.20to8.85)	-0.68(-0.81to-0.54)
Dietary iron deficiency	-	-	378.01(339.90to416.12)	-0.86(-1.96to0.25)
Other nutritional deficiency	-	-	7.33(4.27to12.58)	0.29(-0.70to1.30)
**Tuvalu**				
Nutritional deficiencies	835.46(778.81to892.12)	0.70(0.49to0.91)	632.16(582.88to681.44)	-0.56(-0.81to-0.31)
Protein-energy malnutrition	829.81(773.35to886.27)	0.73(0.52to0.94)	41.15(28.57to53.72)	-1.73(-2.03to-1.43)
Iodine deficiency	5.66(0.00to2143035.34)	-2.10(-2.58to-1.62)	2.28(0.00to1378713962.00)	-5.51(-5.75to-5.26)
Vitamin A deficiency	7776.52(7603.68to7949.37)	-2.50(-2.85to-2.15)	1.79(0.00to1.47E+10)	-1.33(-1.47to-1.20)
Dietary iron deficiency	-	-	577.46(530.36to624.56)	-0.50(-0.77to-0.22)
Other nutritional deficiency	-	-	9.48(0.00to193059.06)	4.18(2.01to6.40)
**Uganda**				
Nutritional deficiencies	477.92(435.07to520.77)	0.18(0.07to0.29)	253.04(221.86to284.21)	-4.24(-4.51to-3.96)
Protein-energy malnutrition	410.07(370.38to449.76)	0.60(0.48to0.73)	74.74(57.80to91.69)	-7.14(-7.76to-6.53)
Iodine deficiency	67.85(51.71to83.99)	-1.88(-1.95to-1.82)	28.08(17.69to38.46)	-3.46(-3.67to-3.26)
Vitamin A deficiency	8985.20(8799.41to9170.99)	-4.89(-5.09to-4.69)	3.04(2.27to4.08)	-0.75(-0.89to-0.61)
Dietary iron deficiency	-	-	133.79(111.12to156.46)	-1.86(-2.05to-1.67)
Other nutritional deficiency	-	-	13.39(11.64to15.40)	-7.16(-7.51to-6.80)
**Ukraine**				
Nutritional deficiencies	398.87(359.72to438.01)	2.01(1.08to2.94)	98.07(78.66to117.48)	-3.64(-3.99to-3.29)
Protein-energy malnutrition	380.86(342.61to419.11)	2.23(1.23to3.24)	17.34(15.34to19.60)	-1.40(-2.43to-0.35)

Iodine deficiency	18.01(15.97to20.31)	-1.52(-1.90to-1.15)	22.87(13.49to32.24)	-2.82(-3.16to-2.47)
Vitamin A deficiency	1938.97(1852.66to2025.27)	-2.97(-3.53to-2.42)	0.00(0.00to0.00)	-
Dietary iron deficiency	0.00(0.00to0.00)	-	47.17(33.71to60.63)	-4.72(-5.61to-3.81)
Other nutritional deficiency	0.00(0.00to0.00)	-	10.70(9.15to12.50)	-3.23(-4.22to-2.23)
**United Arab Emirates**				
Nutritional deficiencies	744.93(691.44to798.43)	0.36(0.32to0.39)	387.74(349.15to426.34)	-0.25(-0.75to0.26)
Protein-energy malnutrition	698.49(646.69to750.29)	0.45(0.41to0.49)	11.38(7.82to16.57)	0.80(0.73to0.88)
Iodine deficiency	46.45(33.09to59.80)	-0.77(-0.87to-0.67)	18.39(13.68to24.71)	-0.81(-0.97to-0.65)
Vitamin A deficiency	1118.74(1053.18to1184.29)	-5.23(-5.41to-5.05)	0.00(0.00to0.00)	-
Dietary iron deficiency	-	-	352.74(315.93to389.55)	-0.29(-0.84to0.26)
Other nutritional deficiency	-	-	5.24(3.01to9.11)	4.16(2.17to6.18)
**United Kingdom**				
Nutritional deficiencies	1489.15(1413.52to1564.79)	1.18(0.88to1.48)	163.45(138.39to188.51)	0.31(0.05to0.57)
Protein-energy malnutrition	1422.39(1348.47to1496.31)	1.22(0.91to1.53)	62.84(47.30to78.38)	1.46(1.16to1.75)
Iodine deficiency	66.77(50.75to82.78)	0.25(0.15to0.34)	14.56(13.04to16.27)	0.22(0.12to0.32)
Vitamin A deficiency	441.62(400.43to482.81)	-1.21(-1.27to-1.15)	0.00(0.00to0.00)	-
Dietary iron deficiency	0.00(0.00to0.00)	-	56.83(42.05to71.61)	-2.37(-2.45to-2.29)
Other nutritional deficiency	0.00(0.00to0.00)	-	29.22(18.63to39.81)	4.71(2.77to6.69)
**United Republic of Tanzania**				
Nutritional deficiencies	361.46(324.19to398.72)	-0.62(-0.86to-0.38)	435.49(394.59to476.39)	-2.48(-2.96to-2.00)
Protein-energy malnutrition	327.07(291.63to362.52)	-0.42(-0.71to-0.14)	85.84(67.68to103.99)	-2.11(-2.89to-1.33)
Iodine deficiency	34.38(22.89to45.88)	-2.11(-2.28to-1.94)	14.56(13.01to16.29)	-4.50(-4.69to-4.30)
Vitamin A deficiency	12092.13(11876.60to12307.66)	-3.09(-3.42to-2.75)	5.77(4.82to6.90)	1.07(0.00to2.16)
Dietary iron deficiency	-	-	313.55(278.85to348.26)	-2.36(-2.73to-1.99)
Other nutritional deficiency	-	-	15.77(14.16to17.58)	-4.65(-5.83to-3.46)
**United States of America**				
Nutritional deficiencies	700.76(648.88to752.65)	-0.69(-0.92to-0.46)	138.91(115.81to162.01)	0.71(-0.25to1.68)
Protein-energy malnutrition	684.25(632.98to735.51)	-0.71(-0.94to-0.47)	19.44(18.62to20.29)	-0.76(-1.06to-0.46)
Iodine deficiency	16.52(15.77to17.31)	0.00(-0.09to0.10)	3.63(3.29to4.01)	-0.07(-0.10to-0.03)
Vitamin A deficiency	582.23(534.93to629.52)	-1.38(-1.65to-1.12)	0.00(0.00to0.00)	-
Dietary iron deficiency	0.00(0.00to0.00)	-	108.01(87.64to128.38)	0.93(-0.37to2.24)
Other nutritional deficiency	0.00(0.00to0.00)	-	7.83(7.32to8.38)	2.44(0.64to4.27)
**United States Virgin Islands**				
Nutritional deficiencies	733.13(680.06to786.20)	-0.48(-0.60to-0.35)	374.92(336.96to412.87)	-1.66(-2.14to-1.18)
Protein-energy	724.86(672.09to777.63)	-0.42(-0.54to-0.30)	31.61(20.59to42.63)	-1.98(-2.04to-1.92)

malnutrition				
Iodine deficiency	8.27(0.19to368.36)	-4.40(-4.59to-4.21)	3.43(0.01to1248.92)	-5.29(-5.61to-4.96)
Vitamin A deficiency	1332.59(1261.04to1404.14)	-4.58(-4.78to-4.38)	0.00(0.00to0.00)	-
Dietary iron deficiency	-	-	329.32(293.75to364.89)	-1.70(-2.27to-1.13)
Other nutritional deficiency	-	-	10.56(0.37to303.97)	2.37(0.54to4.25)
**Uruguay**				
Nutritional deficiencies	559.74(513.37to606.11)	1.19(0.36to2.02)	149.96(125.96to173.97)	-1.08(-1.41to-0.74)
Protein-energy malnutrition	551.77(505.73to597.81)	1.22(0.37to2.07)	23.41(13.93to32.90)	0.89(0.34to1.44)
Iodine deficiency	7.97(4.23to15.02)	-0.57(-0.59to-0.55)	2.15(0.63to7.28)	-0.67(-0.78to-0.57)
Vitamin A deficiency	3699.88(3580.66to3819.10)	0.01(-0.86to0.89)	0.00(0.00to0.00)	-
Dietary iron deficiency	-	-	115.23(94.19to136.27)	-1.66(-1.91to-1.41)
Other nutritional deficiency	-	-	9.18(5.09to16.56)	4.11(1.76to6.53)
**Uzbekistan**				
Nutritional deficiencies	296.64(262.88to330.40)	0.10(-0.20to0.39)	900.79(841.96to959.61)	-1.78(-1.97to-1.58)
Protein-energy malnutrition	267.42(235.37to299.48)	0.57(0.21to0.93)	12.13(10.38to14.17)	0.22(0.00to0.44)
Iodine deficiency	29.22(18.62to39.81)	-2.86(-3.03to-2.69)	26.04(16.04to36.04)	-4.94(-5.29to-4.59)
Vitamin A deficiency	2549.37(2450.41to2648.34)	-2.80(-3.35to-2.24)	3.70(2.79to4.91)	-0.66(-0.85to-0.46)
Dietary iron deficiency	-	-	848.72(791.62to905.82)	-1.54(-1.77to-1.32)
Other nutritional deficiency	-	-	10.20(8.61to12.08)	-7.94(-8.32to-7.55)
**Vanuatu**				
Nutritional deficiencies	773.01(718.52to827.51)	-0.34(-0.80to0.12)	903.73(844.81to962.65)	1.49(1.11to1.87)
Protein-energy malnutrition	728.77(675.86to781.68)	-0.34(-0.86to0.17)	52.19(38.03to66.35)	-1.55(-2.43to-0.66)
Iodine deficiency	44.25(31.21to57.29)	-0.34(-0.64to-0.04)	19.93(5.25to75.64)	-0.61(-0.96to-0.25)
Vitamin A deficiency	13545.46(13317.35to13773.57)	-2.01(-2.36to-1.66)	1.39(0.01to217.27)	-0.39(-0.61to-0.17)
Dietary iron deficiency	-	-	821.96(765.77to878.15)	1.80(1.33to2.28)
Other nutritional deficiency	-	-	8.26(1.04to65.57)	3.16(1.47to4.88)
**Venezuela**				
Nutritional deficiencies	535.89(490.52to581.26)	0.96(0.43to1.48)	138.30(115.25to161.35)	-3.67(-3.99to-3.35)
Protein-energy malnutrition	513.64(469.22to558.06)	1.02(0.46to1.59)	23.40(13.92to32.88)	-4.30(-5.03to-3.55)
Iodine deficiency	22.25(13.01to31.50)	-0.37(-0.64to-0.11)	14.65(12.52to17.15)	-6.97(-9.02to-4.87)
Vitamin A deficiency	4154.43(4028.10to4280.76)	-0.12(-2.51to2.33)	2.97(2.09to4.21)	-0.25(-0.36to-0.15)
Dietary iron deficiency	-	-	92.94(74.04to111.83)	-3.01(-3.21to-2.81)
Other nutritional deficiency	-	-	4.34(3.25to5.80)	-8.47(-9.67to-7.25)
**Viet Nam**				
Nutritional deficiencies	1424.87(1350.88to1498.85)	1.25(1.08to1.42)	214.44(185.74to243.14)	-4.55(-4.84to-4.26)
Protein-energy malnutrition	1345.90(1274.00to1417.81)	1.85(1.68to2.02)	33.05(21.78to44.32)	2.84(2.72to2.96)

Iodine deficiency	78.96(61.55to96.38)	-3.88(-4.02to-3.74)	28.33(17.90to38.76)	-10.37(-12.37to-8.32)
Vitamin A deficiency	514.92(470.44to559.40)	-3.96(-6.13to-1.75)	0.78(0.54to1.12)	-0.47(-0.72to-0.22)
Dietary iron deficiency	-	-	136.56(113.66to159.47)	-5.12(-5.22to-5.03)
Other nutritional deficiency	-	-	15.72(14.50to17.04)	5.89(3.91to7.91)
**Yemen**				
Nutritional deficiencies	721.63(668.98to774.28)	2.37(1.59to3.15)	1210.12(1141.93to1278.30)	0.70(0.59to0.82)
Protein-energy malnutrition	657.12(606.87to707.36)	2.71(1.87to3.55)	47.73(34.19to61.27)	-4.99(-5.31to-4.67)
Iodine deficiency	64.52(48.77to80.26)	-1.43(-1.53to-1.34)	94.61(75.55to113.68)	-3.15(-3.20to-3.09)
Vitamin A deficiency	9650.00(9457.46to9842.54)	-6.34(-6.67to-6.02)	6.86(5.50to8.55)	-0.33(-0.48to-0.19)
Dietary iron deficiency	-	-	1051.85(988.29to1115.42)	1.81(1.58to2.04)
Other nutritional deficiency	-	-	9.06(7.48to10.98)	1.30(0.54to2.06)
**Zambia**				
Nutritional deficiencies	525.56(480.63to570.49)	-0.36(-0.60to-0.12)	401.96(362.66to441.25)	-2.96(-3.07to-2.86)
Protein-energy malnutrition	448.03(406.54to489.51)	0.29(0.06to0.53)	133.13(110.51to155.74)	-4.74(-4.96to-4.51)
Iodine deficiency	77.53(60.27to94.79)	-2.92(-3.28to-2.55)	28.83(18.31to39.35)	-3.73(-4.26to-3.21)
Vitamin A deficiency	7628.48(7457.29to7799.66)	-4.31(-4.59to-4.04)	7.07(5.33to9.37)	-1.21(-1.31to-1.10)
Dietary iron deficiency	-	-	211.65(183.14to240.16)	0.13(0.02to0.24)
Other nutritional deficiency	-	-	21.28(12.24to30.32)	-7.28(-7.68to-6.88)
**Zimbabwe**				
Nutritional deficiencies	789.51(734.44to844.58)	0.97(0.89to1.04)	555.54(509.34to601.74)	4.44(3.64to5.25)
Protein-energy malnutrition	628.73(579.58to677.87)	0.99(0.95to1.04)	162.48(137.50to187.46)	8.61(7.79to9.43)
Iodine deficiency	160.78(135.93to185.63)	0.86(0.62to1.10)	47.60(34.08to61.13)	1.17(0.92to1.42)
Vitamin A deficiency	17783.68(17522.30to18045.05)	1.31(0.98to1.65)	6.32(4.86to8.21)	-0.51(-0.83to-0.18)
Dietary iron deficiency	-	-	327.04(291.60to362.49)	3.03(1.53to4.56)
Other nutritional deficiency	-	-	12.10(11.45to12.78)	5.97(4.66to7.29)

Note: The "-" and "0.00(0.00to0.00)" in the supplemental file indicate "no data" and "data available but no deficiency occurred" respectively.
